# Structural insights into bacterial dimethylsulfoniopropionate import by BCCT-family transporters

**DOI:** 10.1038/s44318-026-00798-w

**Published:** 2026-05-08

**Authors:** Yu-Zhong Zhang, Wen-Jing Zhu, Kang Li, Hai-Tao Ding, Motoyuki Hattori, Shuaimeng Liu, Chang Ge, Qi-Long Qin, Zhao-Jie Teng, Ning-Hua Liu, Hai-Yan Cao, Chun-Yang Li, Xiu-Lan Chen, Qing-Tao Shen, Jonathan D Todd, Lu-Ning Liu, Peng Wang

**Affiliations:** 1 MOE Key Laboratory of Evolution and Marine Biodiversity, State Key Laboratory of Marine Food Processing and Safety Control, College of Marine Life Sciences & Frontiers Science Center for Deep Ocean Multispheres and Earth System, Ocean University of China, Qingdao, China; 2 Marine Biotechnology Research Center, State Key Laboratory of Microbial Technology, Shandong University, Qingdao, China; 3 Laboratory for Marine Biology and Biotechnology, Qingdao Marine Science and Technology Center & Laoshan Laboratory, Qingdao, China; 4 Antarctic Great Wall Ecology National Observation and Research Station, Polar Research Institute of China, Ministry of Natural Resources, Shanghai, China; 5 State Key Laboratory of Genetics and Development of Complex Phenotypes, Collaborative Innovation Center of Genetics and Development, Department of Physiology and Neurobiology, School of Life Sciences, Fudan University, Shanghai, China; 6 School of Life Sciences, Department of Chemical Biology, Southern University of Science and Technology, Shenzhen, China; 7 School of Biological Sciences, University of East Anglia, Norwich, UK; 8 Quadram Institute Bioscience, Norwich Research Park, Norwich, UK; 9 Institute of Systems, Molecular and Integrative Biology, University of Liverpool, Liverpool, UK

**Keywords:** Evolution & Ecology, Microbiology, Virology & Host Pathogen Interaction, Structural Biology

## Abstract

Dimethylsulfoniopropionate (DMSP) is a ubiquitous marine organosulfur compound central to microbial stress responses, chemotaxis, and nutrient cycling. Its catabolism produces dimethylsulfide (DMS), a climate-active gas, and plays a key role in the global sulfur cycle. However, the molecular basis of DMSP import, underpinning its microbial metabolism, remains poorly understood. Here, we identify and characterize the BCCT-family transporter DddT from *Psychrobacter* sp. D2, a marine gamma-proteobacterium that utilizes DMSP as a carbon source. DddT is essential for DMSP uptake and functions as a Na^+^-coupled symporter driven by the transmembrane sodium gradient. Using cryo-electron microscopy, we determined DddT structures in multiple conformational states, revealing its Na^+^-dependent transport mechanism involving two sodium ions, one coordinated by a previously uncharacterized binding site. Sequence analysis shows that DddT-like proteins with conserved sodium-binding features are widespread in marine bacteria, suggesting this Na^+^-coupled transport mechanism represents a broadly conserved feature of the BCCT family. Our findings provide mechanistic insights into sodium-driven substrate uptake and marine sulfur cycling.

## Synopsis

DddT is a Na^+^-coupled symporter mediating DMSP uptake in marine bacteria. Cryo-EM structures reveal a Na^+^-dependent mechanism driving DMSP transmembrane transport.

- Identified DddT as a BCCT-family Na^+^-coupled symporter mediating DMSP uptake in *Psychrobacter* sp. D2.
- Determined cryo-EM structures of DddT in multiple conformational states, revealing a novel Na^+^-binding site and the Na^+^-dependent transport mechanism of DddT.
- Found that DddT-like proteins with conserved sodium-binding features are widespread among marine bacteria.

## Abstract (web summary)

DddT is a Na^+^-coupled symporter for climate-active DMSP in marine bacteria.

## Graphical abstract

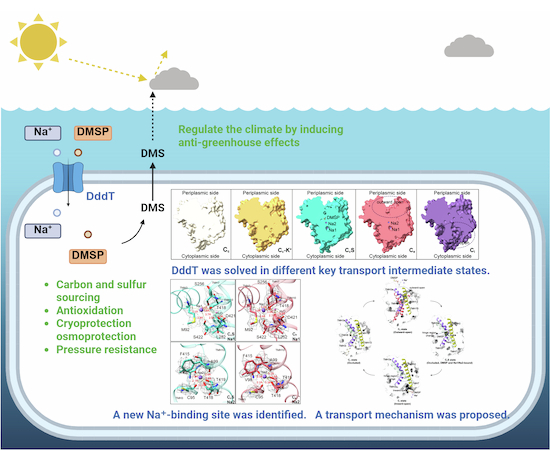

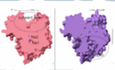

## Introduction

Dimethylsulfoniopropionate (DMSP) is a ubiquitous organosulfur compound in the Earth’s marine environment that plays an important role in the global sulfur cycling (Curson et al, 2011; Teng et al, 2021). Over eight billion tons of DMSP are produced annually by diverse marine algae, some bacteria, corals, and plants (Galí et al, 2015; Zhang et al, 2019), constituting up to 10% of the fixed carbon in certain oceanic regions (Archer et al, 2001; Simó et al, 2002). These organisms produce and accumulate DMSP, which has diverse roles in osmoprotection, cryoprotection, oxidative stress protection, and hydrostatic pressure mitigation (Cosquer et al, 1999; Stefels, 2000; Sunda et al, 2002; Zheng et al, 2020). As a result of cell senescence, mortality, or viral lysis, DMSP is released into the environment, where it reaches nano-micromolar concentrations. In bulk seawater, DMSP concentrations are typically in the range of 10–200 nM; however, substantially higher concentrations, often reaching the micromolar range, can be found in localized microenvironments such as phytoplankton phycospheres and decaying algal particles (Gao et al, 2020; Güell-Bujons et al, 2024; Hopkins et al, 2023; Kiene et al, 2019; Zhang et al, 2019). This environmental DMSP is imported, often concentrated to millimolar concentrations (Kiene et al, 2000), and utilized for its antistress properties or as a major nutrient by marine microorganisms (Zhang et al, 2019). In the latter scenario, microbial DMSP catabolism is a major source of reduced carbon and sulfur for assimilation (Wirth et al, 2020) and climate-active gases, including methanethiol via DMSP demethylation and over 300 million tons of DMS annually via DMSP cleavage (Curson et al, 2011). Approximately 10% of produced dimethylsulfide (DMS) is released into the atmosphere (Curson et al, 2011; Kiene et al, 2000), where its oxidation products act as cloud condensation nuclei (Mungall et al, 2016; Veres et al, 2020) to influence the Earth’s albedo and climate (Hopkins et al, 2023; Veres et al, 2020) and are returned to land in rain and snowfall to complete the sulfur cycle. While many DMSP producers can catabolize DMSP, the majority of DMSP catabolism is thought to be driven by bacteria that import DMSP from the environment (Bullock et al, 2017; Reisch et al, 2011; Shaw et al, 2022).

Recent studies have shed light on the biodiversity and molecular mechanisms of DMSP production and catabolism in bacteria and algae (Carrión et al, 2023; Curson et al, 2011; Shaw et al, 2022). However, there are few mechanistic studies on DMSP import, which is intrinsically necessary for most of the global DMSP catabolism. Indeed, all primary DMSP cleavage and demethylation enzymes have *K*_m_ values of 0.5 to 2 mM (Yoch et al, 1997), and, thus, require intracellular concentration of DMSP to mM levels (Reisch et al, 2008; Sun et al, 2016; Yoch, 2002) that far surpass those in seawater (Belviso et al, 1993; Taylor and Gilchrist, 1991). Two distinct protein families of transporters are known to import DMSP: the betaine-carnitine-choline transporter (BCCT) family and the ATP-binding cassette (ABC) transporter system (Sun et al, 2012), the genes of which are often linked to DMSP lyase genes in bacteria that assimilate DMSP (Curson et al, 2010; Todd et al, 2010; Todd et al, 2007). Representative transporters of the BCCT family, such as *Marinomonas* sp. MWYL1, *Halomonas* sp. HTNK1 and *Vibrio parahaemolyticus* RIMD2210633 and the ABC family from *Ruegeria pomeroyi* DSS-3, *Burkholderia ambifaria*, and *Bacillus subtilis* have been shown to import DMSP in various genetic studies (Broy et al, 2015; Cosquer et al, 1999; Li et al, 2023; Todd et al, 2010; Todd et al, 2007). This study focuses on the BCCT family transporter DddT in organisms such as *Marinomonas*, *Halomonas*, *Psychrobacter*, and *Pseudoalteromonas*, which cleave DMSP to generate DMS and assimilate carbon from DMSP, whose *dddT* genes cluster with the DMSP lyase *dddD* or *dddX* and ancillary DMSP catabolic genes, there have been no gene mutagenic studies on *dddT* (Curson et al, 2010; Curson et al, 2011; Li et al, 2021; Todd et al, 2010; Todd et al, 2007). There is also an absence of detailed biochemical, structural, and dynamic simulation analysis for DddT. The physiology and molecular mechanisms of DddT-dependent DMSP transport have not been well characterized.

BCCT family transporters are common in diverse prokaryotes and eukaryotes, and often require ion gradients for function, aiding survival in environments with high osmotic pressure (Ziegler et al, 2010). The glycine betaine transporter BetP from *Corynebacterium glutamicum* is the best-characterized sodium symporter in the BCCT family (Krämer and Morbach, 2004; Ressl et al, 2009). Although extensive mechanistic studies have proposed that the transport mechanism of BetP involves two sodium ion-binding sites, Na1 and Na2, the location of Na1 has not yet been directly visualized in any structure to date (Khafizov et al, 2012; Perez et al, 2014; Perez et al, 2012). There is significant amino acid diversity within the BCCT family of transporters, and subsequent variations exist in their mechanisms and substrate range; for example, BetT is a proton-coupled transporter (Chen and Beattie, 2008), BetP is a sodium-coupled betaine transporter (Khafizov et al, 2012), and CaiT is a Na^+^-independent substrate/product antiporter (Kalayil et al, 2013; Schulze et al, 2010). Given this variation in the action of the BCCT-family transporters, there is a critical need to gain a mechanistic understanding of DMSP transport by DddT.

Here, we investigated the roles of DddT in the import and catabolism of DMSP in *Psychrobacter* sp. D2, a marine gamma-proteobacterium isolated from Antarctic samples, cleaves and utilizes DMSP as a carbon source (Li et al, 2021). The ability of *P*. sp. D2 DddT to import DMSP and its requirement for the sodium gradient D2 DddT protein was further characterized. Three-dimensional DddT structures were resolved using cryo-electron microscopy in different states of DMSP transport, including the closed, DMSP-bound closed, inward-open, and outward-open conformations. Analyses of these structures highlighted a transport mechanism involving two sodium ions, one coordinated by a previously uncharacterized binding site. Furthermore, bioinformatic analysis was conducted to examine the ubiquity of the conserved sodium-binding sites among diverse DddT-like proteins within organisms and environments. This work implies that DddT-like proteins and their associated processes, as identified in this study, are widespread and play a significant role in the Earth’s marine environment.

## Results and discussion

### Function of DddT from *P*. sp. D2

**Figure 1:**
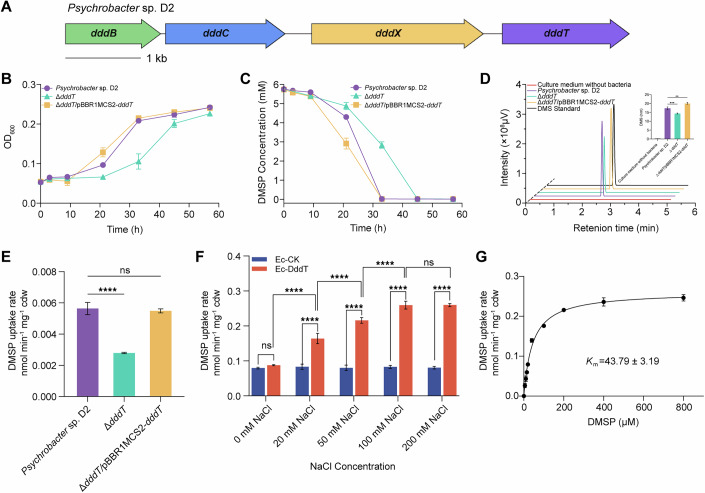
Role of *dddT* in *P*. sp. D2 DMSP transportation. (**A**) DMSP catabolic operon *dddBCXT* in *P*. sp. D2. (**B**, **C**) Growth curves (**B**) and DMSP utilization (**C**) of wild-type *P*. sp. D2 strain, Δ*dddT* mutant, and the complemented mutant (Δ*dddT*/pBBR1MCS2-*dddT*). All cultures were cultivated with 6 mM DMSP as the sole carbon source. (**D**) DMS production of wild-type *P*. sp. D2 strain, Δ*dddT* mutant, and the complemented mutant after 21 h culture in the medium with 6 mM DMSP as the sole carbon source. (**E**) DMSP uptake rates of *P*. sp. D2, Δ*dddT* mutants, and the complemented mutant. cdw is short for cell dry weight. (**F**) DMSP uptake rates of *E. coli* DH5α cells with cloned *dddT* or empty vector at varying sodium levels. (**G**) DMSP uptake rates of *E. coli* DH5α cells with cloned *dddT* or empty vector under saturated sodium ion conditions (200 mM NaCl) and different DMSP concentrations. Data information: (**B**–**G**) Data are presented as the mean ± SD of triplicate determinations. (**D**) Statistical analysis was performed using one-way ANOVA (*P*. sp. D2 vs. Δ*dddT, p* = 0.0001; *P*. sp. D2 vs. Δ*dddT*/pBBR1MCS2-*dddT*, *P* = 0.0015). (**E**) Statistical analysis was performed using one-way ANOVA (*P*. sp. D2 vs. Δ*dddT*, *P* < 0.0001; *P*. sp. D2 vs. Δ*dddT*/pBBR1MCS2-*dddT*, *P* = 0.6772). (**F**) Statistical analysis was performed using two-way ANOVA (****P < 0.0001; 0 mM NaCl (Ec-CK) vs. 0 mM NaCl (Ec-DddT), *P* = 0.9731; 100 mM NaCl (Ec-DddT) vs. 200 mM NaCl (Ec-DddT), *P* > 0.9999). Source data are available online for this figure.

The *dddT* gene is the last of the four *dddBCXT* operons (Fig. 1A) in *P*. sp. D2, whose transcription was induced by DMSP (Li et al, 2021). This operon has been suggested to allow *P*. sp. D2 to import (via DddT), cleave (via DddX) DMSP, and assimilate 3-hydroxypropionyl-CoA (via DddBC) as the carbon source (Appendix Fig. S1) (Li et al, 2021). When DMSP was provided as the sole carbon source, *P*. sp. D2 grew more slowly than in nutrient-rich 2216E medium, but faster than on other typical single-carbon sources such as succinate or glucose (Appendix Fig. S2).

DddT is a BCCT-family protein that shares 42% amino acid sequence identity with *Halomonas* sp. HTNK1 DddT, which was previously shown to import DMSP when expressed in *E. coli* (Todd et al, 2010) (Appendix Fig. S3A). The *P*. sp. D2 *dddT-*deletion mutant (Δ*dddT*), in which 1584 bp internal to this gene was deleted via homologous recombination (Appendix Fig. S4), was examined to be capable of importing DMSP, utilizing DMSP as the sole carbon source, and cleaving DMSP compared to the wild-type strain and Δ*dddT* genetically complemented with cloned *dddT (*Δ*dddT*/pBBR1MCS2-*dddT*). The Δ*dddT* mutant grew remarkably slower than the wild-type strain, and its growth could be restored to levels similar to those of the wild-type strain with the complemented strain (Δ*dddT*/pBBR1MCS2-*dddT*) (Fig. 1B). Following extended incubation, the Δ*dddT* mutant achieved comparable final optical densities as the wild-type strain. Consistently, extracellular DMSP levels were similar and depleted more rapidly (Fig. 1C) in both the wild-type and Δ*dddT*/pBBR1MCS2-*dddT* strains. These two strains also accumulated twice the amount of DMSP (Fig. 1E) compared to Δ*dddT* during these growth experiments. Furthermore, the wild-type and Δ*dddT*/pBBR1MCS2-*dddT* strains produced higher levels of DMS than the Δ*dddT* strain during the exponential growth phase (21 h) (Fig. 1D). These results indicate that the DddT protein in *P*. sp. D2 is essential for optimal growth when DMSP is the only available carbon source, as well as for the breakdown of DMSP. However, these findings also suggest the presence of alternative DMSP transport mechanisms, as other transporters may compensate for the absence of DddT, allowing the Δ*dddT* mutant to eventually utilize DMSP, albeit less efficiently.

To further study the activity of DddT, *P*. sp. D2 *dddT* was cloned into the pET-22b vector, with its promoter replaced by the constitutive promoter BBa-J23111, a moderate-strength promoter widely used for stable gene expression in *E. coli*. *E. coli* cells harboring cloned *dddT* (Ec-DddT) displayed significantly higher transport activity than those harboring the empty plasmid (Ec-CK) but only in the presence of NaCl (Fig. 1F). Moreover, DddT-mediated DMSP import was enhanced as the NaCl concentration was increased to 100 mM; beyond this concentration, the DMSP import rate remained constant, suggesting that it likely reached its maximum (Fig. 1F). These data indicate that DddT is a sodium-ion-gradient-dependent transport protein that acts as a sodium-coupled symporter, similar to most BCCT-family transporters (Ziegler et al, 2010). Under saturated sodium ion conditions, the apparent Michaelis constant (*K*_m_) of DddT for DMSP transport was 43.79 ± 3.19 μM (Fig. 1G).

Although DddT exhibits a relatively high apparent *K*_m_ value compared with bulk seawater DMSP concentrations, localized microenvironments such as phytoplankton phycospheres and decaying algal particles can reach much higher DMSP levels, representing one of the common habitats for *Psychrobacter* species (Gao et al, 2020; Güell-Bujons et al, 2024; Heuchert et al, 2004; Hopkins et al, 2023; Katsuhiro et al, 2024; Kiene et al, 2019; Zhang et al, 2019). Moreover, in heterologous *E. coli* uptake assays, DddT displayed a *V*_max_ of ~0.25 nmol min^−1^ mg^−1^ cdw, substantially lower than that of BetP for glycine betaine (~83 nmol min^−1^ mg^−1^ cdw) (Perez et al, 2014). Uptake rates normalized per mg bacterial dry weight can be remarkably influenced by differences in expression, folding efficiency, and membrane insertion in the heterologous system. Therefore, the numerical difference may not reflect a biologically relevant disparity.

Taken together, despite the relatively low observed transport rate and high apparent *K*_m_, DddT functions as an important DMSP transporter in *P*. sp. D2, enabling efficient utilization of DMSP under relevant environmental conditions, while alternative transport mechanisms may partially compensate in its absence.

### Overall structure of DddT

To study the molecular mechanism of DddT-mediated DMSP transport and its dependence on sodium ion gradients, we expressed and purified recombinant *P*. sp. D2 DddT proteins from *E. coli* C43 (DE3) for structural analysis. The DddT protein, which contains 527 amino acids and has a theoretical molecular weight of 57.7 kDa, exhibited an apparent molecular weight of approximately 39 kDa on SDS-PAGE (Appendix Fig. S3B). Furthermore, gel filtration chromatography analysis revealed that DddT exists as a trimer in solution, consistent with typical members of the BCCT family (Ressl et al, 2009; Schulze et al, 2010) (Appendix Fig. S3C).

**Figure 2:**
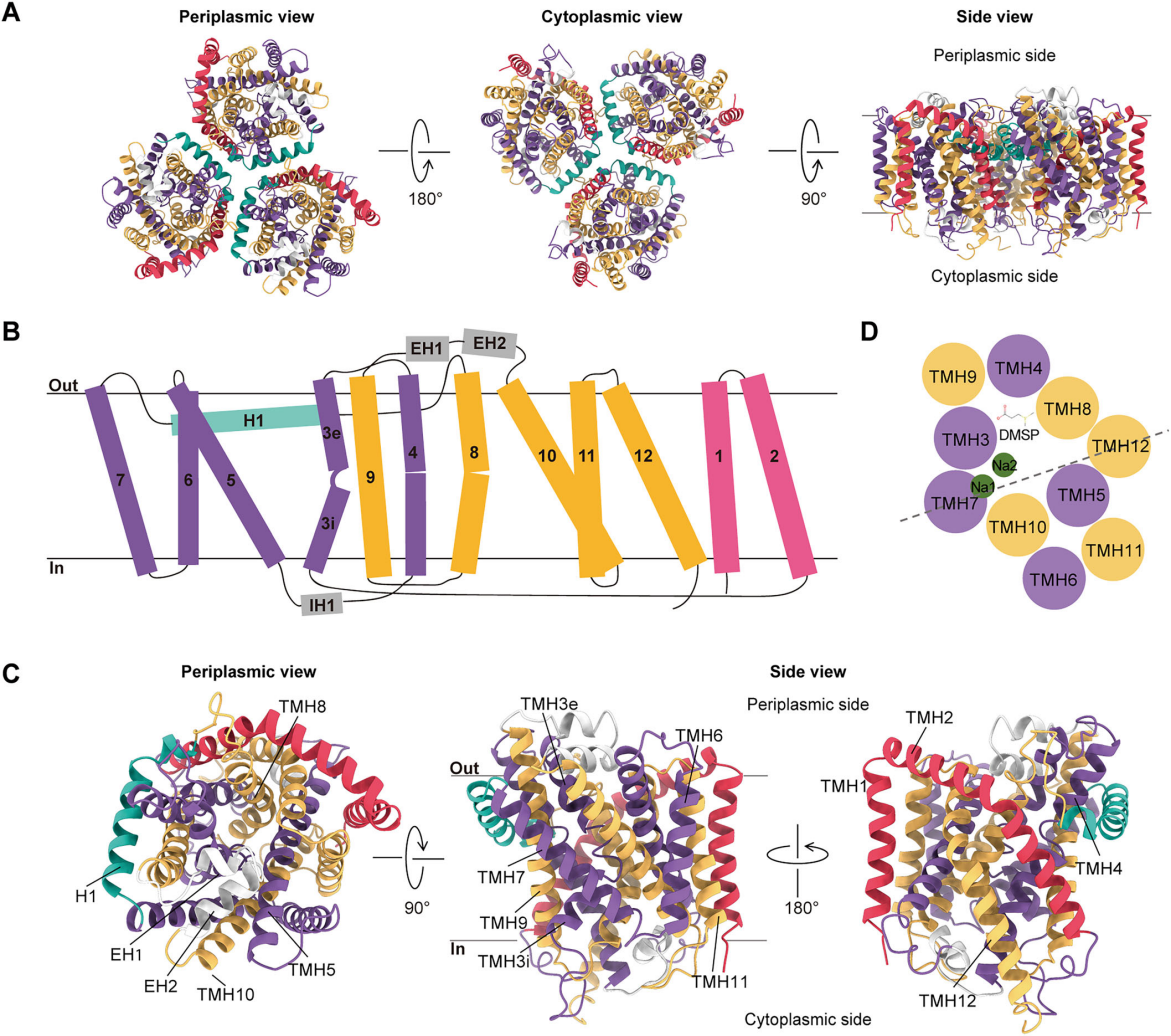
Overall structure of DddT. (**A**) DddT trimer. Left, view from the periplasmic side; middle, view from the cytoplasmic side; right, side view in the membrane plane. (**B**) Schematic view of the topology of DddT. Each DddT monomer is composed of 12 transmembrane α-helices. TMH1 and TMH2 are represented in red, while TMH3-TMH7 and TM8-TMH12 are represented in purple and yellow, respectively. Cytoplasmic α-helix (IH1 and IH2) and periplasmic α-helix (EH2) are gray, and Helix 1 (H1) is blue. (**C**) DddT monomer with its distinct helices labeled. (**D**) Schematics of helix packing in LeuT-like of DddT structure, viewed from the periplasm side and at a slice through the membrane plane roughly midway across the membrane. Repeat 1 (TMH3-TMH7) and repeat 2 (TMH8-TMH12) are colored in purple and yellow, respectively. The approximate locations of ligands and sodium-binding sites are indicated by the chemical structure of DMSP and green circles, respectively.

Next, the DddT structure was resolved to 2.8 Å resolution by cryo-electron microscopy single-particle analysis (Fig. 2A; Appendix Figs. S5 and S6; Appendix Table S1), revealing that DddT adopts a closed substrate-free state (C_c_) in a buffer containing 20 mM Tris-HCl (pH 8.0), 200 mM NaCl, and 0.02% n-dodecyl-β-D-maltopyranoside (DDM). For cryo-EM data processing, C3 symmetry was not imposed during the initial refinement. The map was first reconstructed in C1, and the three protomers were compared, revealing no notable conformational differences. Based on this observation, C3 symmetry was subsequently applied to improve map quality and final resolution. The DddT protein comprises three monomers, each of which adopts a cylindrical shape with 12 transmembrane (TM) α-helices (TMH1-TMH12), a bent α-helix (H1) situated on the cytoplasmic membrane surface, two short α-helices (EH1 and EH2) on the periplasmic side, and a short α-helix (IH1) on the cytoplasmic side, exhibiting C3 symmetry with an axis perpendicular to the membrane plane (Fig. 2A,C). TMH1, TMH6, and TMH11 are nearly perpendicular to the membrane, whereas TMH5, TMH7, TMH10, and TMH12 are strongly tilted (Fig. 2B). TMH3, with a local unwinding segment in the middle of the membrane, was divided into intracellular-facing (TMH3i) and extracellular-facing (TMH3e) helices (Fig. 2B). The cytoplasmic loop connecting both TMH4 and TMH5 contains a short α-helix (IH1) and a periplasmic loop that links TMH9 and TMH10, incorporating two short α-helices (EH1 and EH2) (Fig. 2B,C).

DddT exhibits a LeuT-like folding pattern (Alamo et al, 2022), resembling other BCCT-family transporters, such as BetP and CaiT (Khafizov et al, 2012; Perez et al, 2012; Ressl et al, 2009; Schulze et al, 2010; Tang et al, 2010), and other sodium symporters, such as LeuT (Krishnamurthy and Gouaux, 2012; Yamashita et al, 2005), DAT (Wang et al, 2015a), SERT (Coleman et al, 2016), MhsT (Malinauskaite et al, 2014), and GlyT1 (Shahsavar et al, 2021) from the Neurotransmitter-Sodium Symporter family, SiaT (Wahlgren et al, 2018), and vSGLT (Faham et al, 2008) from the Sodium-Solute Symporter family, and Mhp1 (Weyand et al, 2008) from the Nucleobase-Cation Symporter-1 family. This fold features five TM helices in topological repeats connected by a pseudo-dyad axis parallel to the membrane surface (Alamo et al, 2022). In DddT, TMH3-TMH7 and TMH8-TMH12 were linked by a pseudo-dyad double-symmetric axis on the membrane plane, with TMH3-TMH7 as the first segment and TMH8-TMH12 as the second segment (Fig. 2D; Appendix Fig. S7A). TMH3 and TMH4 formed a V-shaped helix pair, whereas TMH8 and TMH9 formed an inverted V-shape, tightly nested to create a four-helix bundle (Appendix Fig. S7B). The central structure of DddT is composed of this cluster, which is encircled and reinforced by TMH5-TMH7 and TMH10-TMH12.

### DddT conformations in distinct transport intermediate states

**Figure 3:**
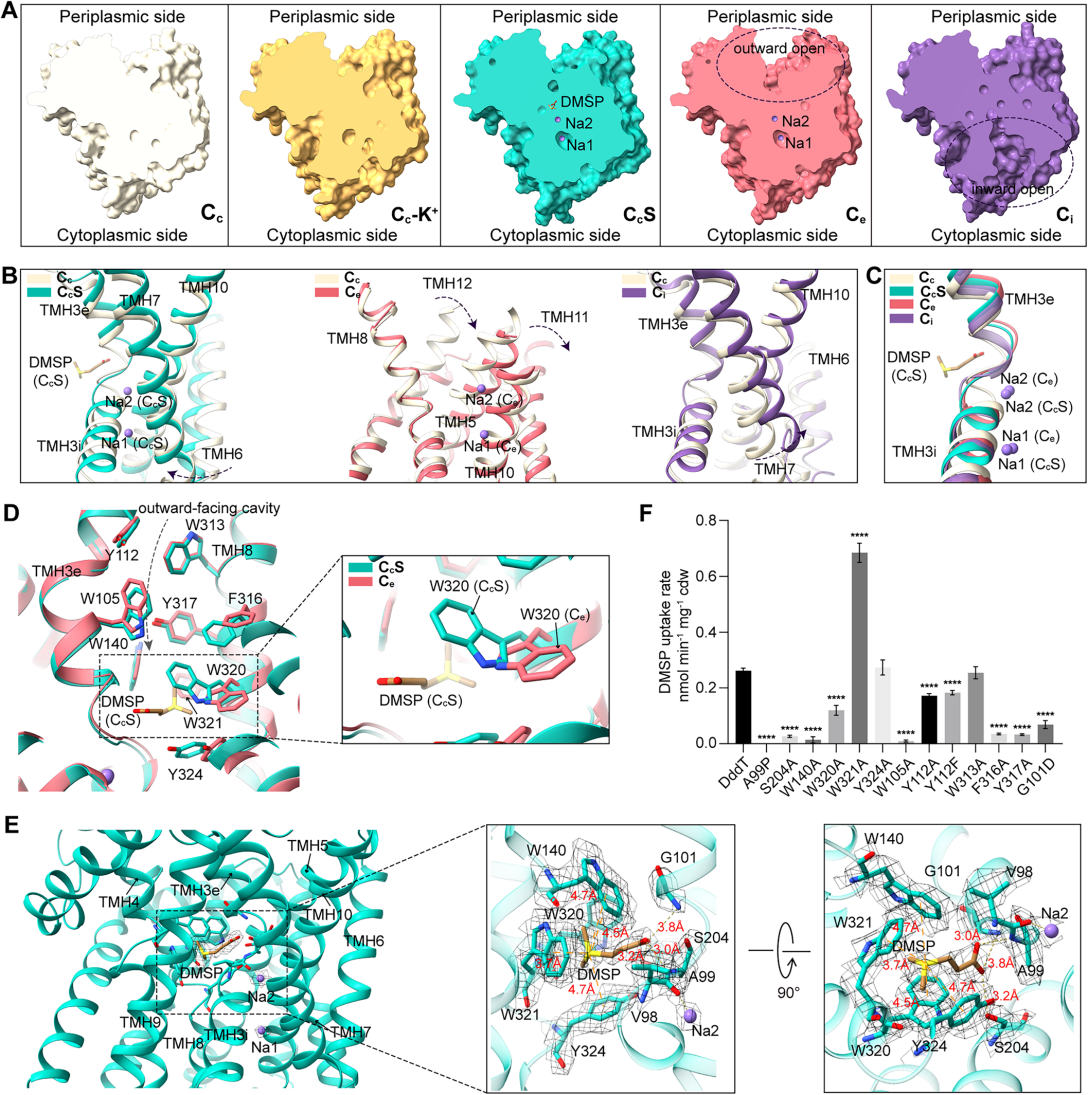
Conformations of DddT in different transport intermediate states and the DMSP binding site. (**A**) Structures of DddT in different intermediate states. Cartoon views of the five DddT conformational states (C_c_, C_c_-K^+^, C_c_S, C_e_, and C_i_) are colored in beige, orange, blue, red, and purple, respectively. Sodium ions are shown as purple spheres. (**B**) Comparison of the conformation of TMH3, TMH6, TMH7, and TMH10 between the C_c_ and C_c_S states, of TMH5, TMH8, TMH10, TMH11, and TMH12 between the C_c_ and C_e_ states, and of TMH3, TMH6, TMH7, and TMH10 between the C_c_ and C_i_ states. (**C**) Comparison of the hinge regions that connect TMH3i and TMH3e within the four states of DddT. (**D**) Comparison of the DMSP binding sites between the C_e_ state (red) and the C_c_S state (blue) of DddT. The extracellular cavity is indicated by a dashed arrow. Residues involved in substrate binding on the extracellular cavity are shown as sticks. (**E**) The DMSP binding site of the C_c_S state. Hydrogen bonds are represented by yellow dashed lines. The distances between DMSP and the aromatic box residues are shown as orange dashed lines. The gray mesh shows the local cryo-EM density around DMSP, Na^+^ (Na2), and the residues involved in sodium binding, contoured at 7 root-mean-square-deviation (RMSD). (**F**) The DMSP uptake rates of DddT and site-directed mutants in predicted DMSP binding residues. The transport capabilities of these mutants were assessed and normalized against expression levels of DddT and its mutants based on western blot analysis. Data are presented as the mean ± SD of triplicate determinations. Statistical analysis was performed using one-way ANOVA (****P < 0.0001; DddT vs. Y324A, *P* = 0.9699; DddT vs W313A, *P* = 0.9997). Source data are available online for this figure.

To investigate the dynamic transport process of DddT, we manipulated buffer compositions, introduced substrates, and introduced amino acid substitutions, allowing capture of four additional DddT structures beyond the C_c_ state (Fig. 3A; Appendix Table S1). Inclusion of DMSP in NaCl buffer enabled capture of DddT in a closed, substrate-bound conformation (C_c_S) (Fig. 3A; Appendix Figs. S8 and S9; Appendix Table S1). When NaCl was replaced with KCl in the buffer, even in the presence of DMSP, DddT adopted the C_c_ conformation without detectable DMSP binding (C_c_-K^+^) (Fig. 3A; Appendix Figs. S10 and S11; Appendix Table S1). Introduction of a G101D mutation in the transport channel in the presence of NaCl allowed capture of the outward-facing, substrate-free state (C_e_) (Fig. 3A; Appendix Figs. S13 and S14; Appendix Table S1), whereas substitution of NaCl with KCl in the same mutant background resulted in the inward-facing state (C_i_) (Fig. 3A; Appendix Figs. S15 and S16; Appendix Table S1).

In the C_c_S state of DddT, a bundle of five helices formed by TMH3, TMH4, TMH5, TMH8, and TMH9 enclosed a distinct non-protein density, which was predicted to correspond to DMSP. In addition, two extra densities located between TMH3, TMH7, and TMH10 were tentatively assigned as sodium ions (Na^+^) (Fig. 3E). There is a change in the transmembrane helix orientation when DMSP and Na^+^ are bound, TMH3, TM6, TMH7 and TMH10 exhibited slight angular deviations relative to the C_c_ conformation, whereas TMH3, TMH7 and TMH10 were near the intracellular region and exhibited some degree of closure (Fig. 3B). Furthermore, a notable conformational transformation was observed in the hinge region connecting TMH3i and TMH3e, forming a pathway for DMSP translocation through TMH3 (Fig. 3C).

In the C_e_ state, the overall structure was characterized by an outward-facing cavity (Fig. 3A), and notable densities, also tentatively assigned as sodium ions, were observed in TMH3, TMH7, and TMH10. These densities were aligned with the positions of the two Na^+^ ions observed in the closed C_c_S state (Fig. 3A). A notable deviation of ~30° was observed in TMH11 and TMH12 near the periplasmic side, while TMH5 in its central region, together with TMH8 and TMH10, exhibited a slight angular shift, resulting in an outward opening of the substrate-binding cavity (Fig. 3B). Furthermore, the hinge region connecting TMH3i and TMH3e in the C_e_ state adopted a conformation similar to that observed in the C_c_S state but distinct from that in the C_c_ state (Fig. 3C). This suggests that the conformational change in the hinge regions is associated with the binding of the two Na^+^ ions rather than with DMSP binding.

**Figure EV1:**
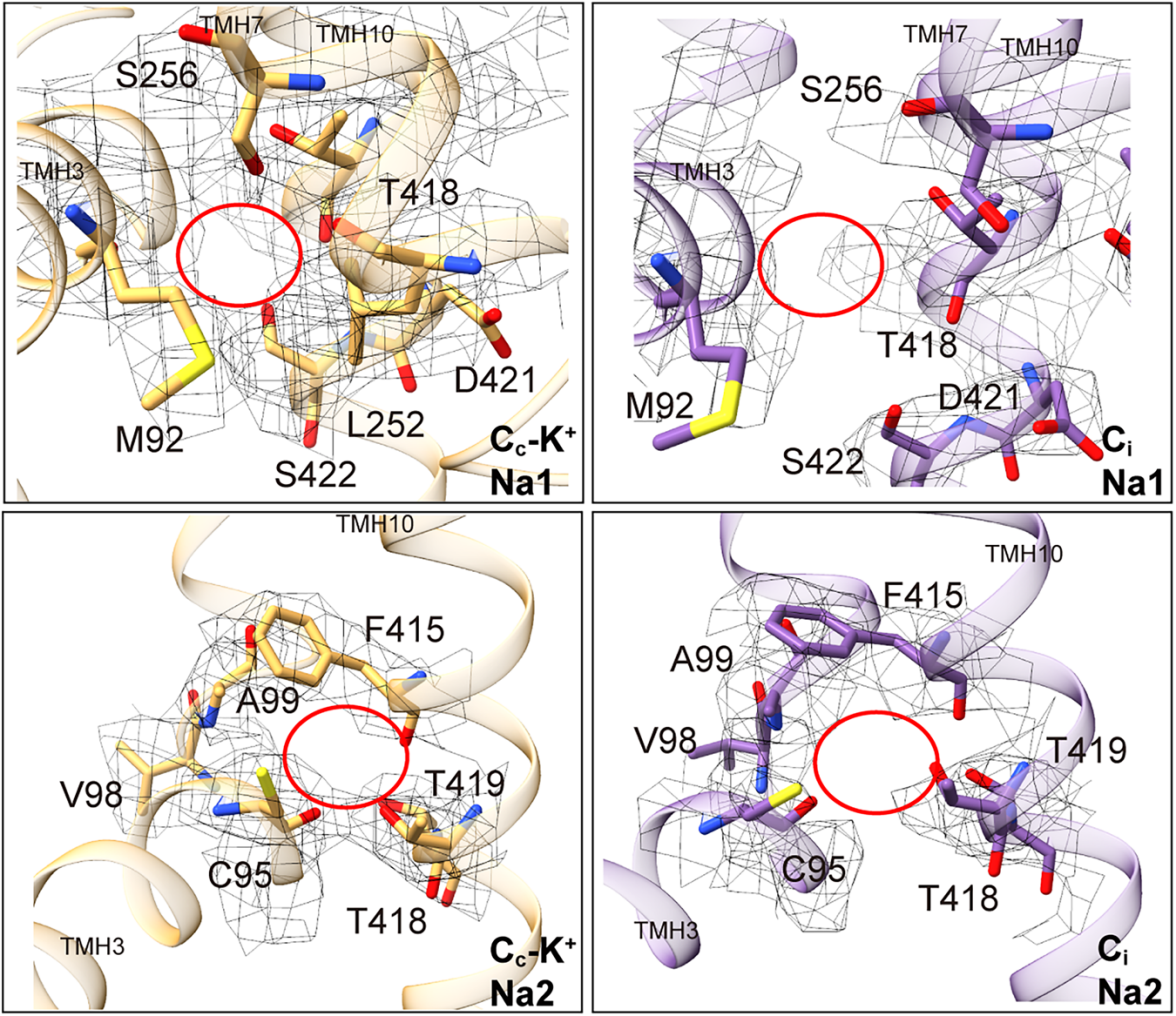
Loss of cryo-EM densities at the putative Na1 and Na2 sites in the C_c_-K^+^ and C_i_ structures determined in Na^+^-free buffer. The gray mesh shows the local cryo-EM density of the residues involved in sodium binding, contoured at 4 RMSD. The densities corresponding to Na1 and Na2, which are clearly observed in the C_e_ and C_c_S states, are absent under Na^+^-free conditions.

In the C_i_ state, the overall structure displayed an inward-facing cavity, and due to the replacement of NaCl with KCl in the buffer, the sodium ion-binding sites corresponding to those in the C_e_ and C_c_S states showed no detectable electron density (Fig. EV1). The distances among TMH3, TMH6, TMH7, and TMH10 increased markedly compared with those in the C_c_ state, contributing to the opening of the inward-facing cavity (Fig. 3B). In comparison with the Na^+^-bound C_e_ state, this observation suggests that Na^+^ binding and release regulate the structural transition of DddT between outward-open and inward-open conformations. Moreover, the hinge regions connecting TMH3i and TMH3e in the C_i_ state adopt an intermediate conformation between those observed in the C_c_ state and the C_c_S/C_e_ states (Fig. 3C). This further supports that Na^+^ binding and release play a key role in driving conformational changes in the hinge region.

**Figure EV2:**
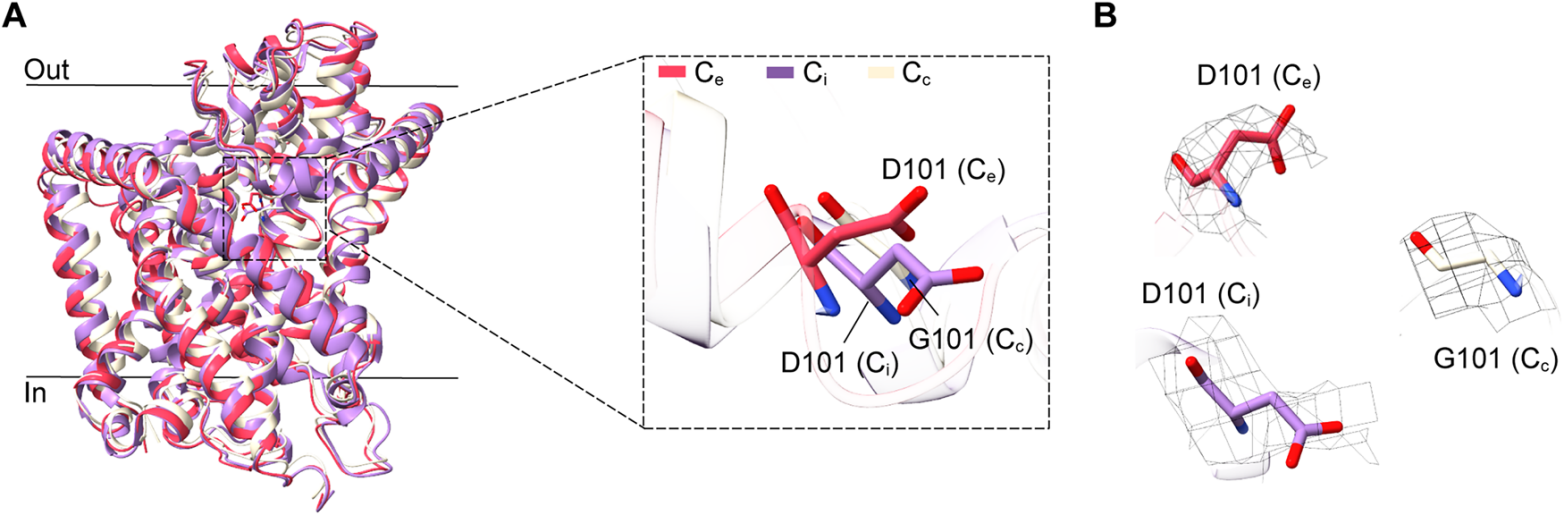
Structural comparison of residue 101 in DddT among the C_c_, C_e_, and C_i_ states. (**A**) Structural differences of residue 101 between the C_c_, C_e_, and C_i_ states. The C_c_, C_e_, and C_i_ states are colored in beige, red, and purple, respectively. (**B**) Cryo-EM densities surrounding Asp101 (C_e_), Asp101 (C_i_), and Gly101 (C_c_), contoured at 6 RMSD, 4 RMSD, and 8 RMSD, respectively.

In the C_c_-K^+^ state, although DMSP was added, no electron density corresponding to DMSP was observed, consistent with the C_c_ state in the presence of NaCl without DMSP (Appendix Fig. S12). Owing to the limitations of single-particle cryo-EM, we cannot completely exclude a minor population of DMSP-bound particles in the KCl sample. However, particle classification and reconstruction indicate that a huge number of particles adopt a DMSP-free conformation. In addition, only this population could be aligned and refined to high resolution, suggesting that the remaining particles likely represent conformationally unstable states. This contrasts sharply with the NaCl condition with DMSP, under which only the DMSP-bound conformation could be aligned and refined to high resolution. Taken together with data of the C_c_, C_c_S, C_i_, and C_e_ states, these findings suggest that stable DMSP binding requires Na^+^, whereas stable Na^+^ binding in turn depends on either DMSP or mutations in TMH3 (e.g., G101) that perturb the hinge region (Figs. 3A and EV2). Thus, DMSP binding, Na^+^ binding, and hinge conformation change are tightly coupled.

### DMSP binding sites in DddT

As mentioned above, DMSP densities were identified in the structures of the closed C_c_S state of DddT. DMSP is proposed to enter the binding site via the outward-facing cavity of the C_e_ state. Structural analysis of this cavity revealed that aromatic amino acid residues, including Trp105 and Tyr112 on TMH3, as well as Trp313, Phe316, and Tyr317 on TMH8, are likely key residues involved in the DMSP entry pathway (Fig. 3D). These residues are presumed to direct DMSP towards a potential aromatic binding pocket formed by Trp320, Trp321, Trp140, and Tyr324 (Fig. 3D,E). In the C_c_S state, DMSP is bound within this pocket, where Arg320, Trp321, Trp140, and Tyr324 form cation–π interactions with the sulfonium (S^+^) group of DMSP, while the side chain of Ser204 and the backbone amide nitrogens of Gly101 and Ala99 form hydrogen bonds with the carboxyl group of DMSP (Fig. 3E). To investigate in depth the roles of these amino acids in DMSP binding, substitution mutations were made in these residues and the transport capabilities of these mutants were assessed, normalized against expression levels of DddT and its mutants based on western blot analysis (Appendix Fig. S17). The results indicated a reduction in transport activity for most mutants compared to wild-type DddT. The A99P, S204A, W140A, W320A, Y112A, Y112F, W105A, F316A, Y317A, and G101D mutants exhibited a significant loss of activity, confirming the critical role of these amino acid residues in DMSP transport (Fig. 3F). Notably, the W321A mutant exhibited enhanced transport activity, suggesting that Trp321 may not play a critical role in DMSP binding and transport. The mechanistic basis for this phenotype merits further investigation.

Compared to the C_e_ state, the C_c_S state exhibits a pronounced conformational change at Trp320 within the DMSP-binding pocket (Fig. 3D), indicating that Trp320 not only constitutes a critical part of the functional binding site for DMSP but also may contribute to promoting its movement during the transport process.

### Na^+^-binding sites in DddT

**Figure 4:**
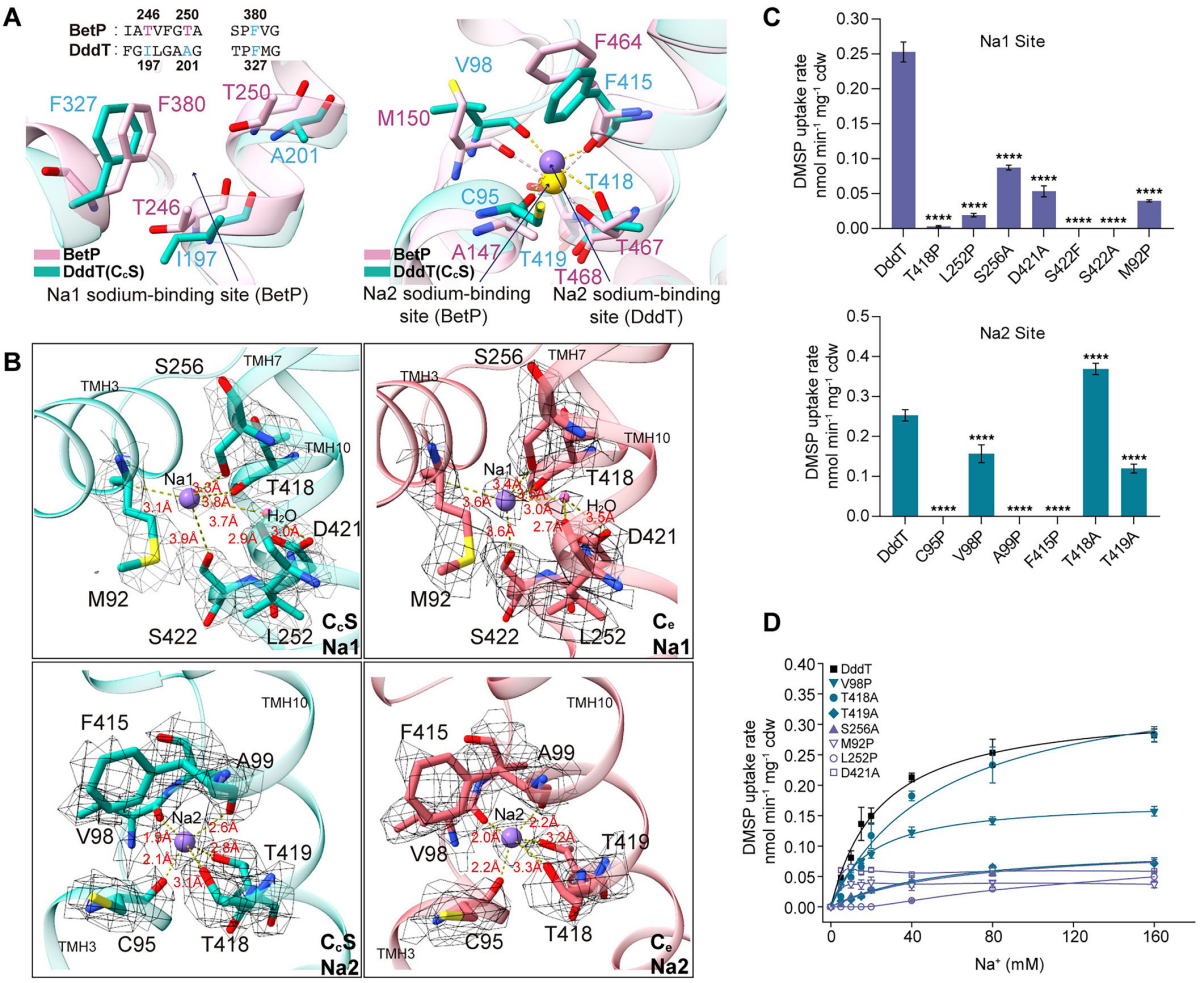
The sodium-binding sites in DddT. (**A**) Structure comparison of BetP and DddT around the BetP Na1 and Na2 sodium-binding sites. The residues of the C_c_S state of DddT and BetP (PDB ID: 4AIN) are colored blue and pink, respectively. The sodium ions in Na2 sodium-binding sites of DddT (C_c_S) and BetP are shown as purple and yellow spheres, respectively. (**B**) Comparison of the Na^+^-binding sites in the C_c_S (blue) and C_e_ (red) states of DddT. Sodium ions and H_2_O molecules are shown as purple and pink spheres, respectively. Residues involved in sodium binding are shown as sticks. The gray mesh shows the local cryo-EM density of Na^+^ (Na1, Na2), H_2_O molecules, and the residues involved in sodium binding, contoured at 8 RMSD. (**C**) DMSP uptake rates of DddT and various site-directed mutant derivatives under saturated sodium ion and DMSP conditions. The transport capabilities of these mutants were assessed and normalized against expression levels of DddT and its mutants based on western blot analysis. Data are presented as the mean ± SD of triplicate determinations. Statistical analysis was performed using one-way ANOVA (****P < 0.0001). (**D**) DMSP uptake rates of DddT and various site-directed mutant derivatives under saturated DMSP and different sodium ion concentrations. The transport capabilities of these mutants were assessed and normalized against expression levels of DddT and its mutants based on western blot analysis. Data are presented as the mean ± SD of triplicate determinations. The kinetic parameters of sodium ion binding of DddT mutants are summarized in Appendix Table S2. Source data are available online for this figure.

As described above, electron density consistent with putative Na⁺ ions was observed in the C_c_S and C_e_ states. Removal of Na^+^ from the buffer abolished this density in the C_c_–K^+^ and C_i_ states at the corresponding position, further strengthening its assignment as Na^+^. While the putative Na2 site in DddT is similar to that found in BetP (Fig. 4A), DddT lacks the BetP Na1 site, and the key residues T246 and T250 necessary for sodium binding are absent (Khafizov et al, 2012; Perez et al, 2014; Perez et al, 2012). Residues Ile197 and Ala201 of DddT, which correspond to the Na1 site of BetP, are hydrophobic and are unable to bind sodium ions (Fig. 4A). In addition, there were no other suitable locations for sodium binding near the BetP Na1 equivalent region in DddT. Instead, we found that DddT has a putative Na1 site closer to the cytoplasmic side. Structural analysis of the C_c_S and C_e_ states suggests that Na1 binding is coordinated by the backbone carbonyl oxygen of Met92 (TMH3); the backbone carbonyl oxygen of Leu252, via a water molecule, and the side chain of Ser256 (TMH7); and the backbone carbonyl oxygen of Thr418, the side chain of Asp421 through a water-mediated interaction, and the side chain of Ser422 (TMH10). Whereas Na2 coordination is presumed to involve the backbone carbonyl oxygens of Cys95 and Val98 (TMH3) and Phe415, together with the side chains of Thr418 and Thr419 (TMH10) (Fig. 4B). Based on the observed coordination environment, Na2 coordination appears relatively stable, whereas Na1 shows longer coordination distances and correspondingly less stable binding. Such variability in Na^+^ coordination is not unusual and has been observed in other transporters. For example, in the BetP transporter, the proposed Na1 site displays a coordination pattern distinct from that of the stable Na2 site (Perez et al, 2014). This instability may be functionally relevant, as discussed in detail below in another section.

To further confirm the Na1 and Na2-binding sites and their functional significance during the transport process, substitution mutants of the residues involved in coordination were generated, and their impact on DddT DMSP transport capability was investigated. Alanine is our primary choice for mutagenesis of these residues. However, for amino acids whose backbone amide oxygen atoms participate in metal ion coordination, alanine substitutions may not significantly disrupt the ion-binding environment. Therefore, we selectively introduced proline mutations, as proline’s rigid backbone is more likely to interfere with backbone-mediated interactions, providing additional biochemical insight. It should be noted, however, that proline substitutions are relatively drastic and have intrinsic limitations. Such substitutions may cause structural effects beyond modification of the peptide bond amide group; therefore, the validation results should be regarded as reference evidence only.

For the mutants involved in Na1-binding and Na2-binding, the transport capabilities were normalized to DddT expression levels (Appendix Fig. S17). For the Na1-binding site, assays conducted under various sodium gradients revealed that the M92P, L252P, S256A, T418P, D421A, and S422A mutations, which are associated with Na1 binding, led to marked changes in DddT transport activity (Fig. 4C,D; Appendix Table S2). For the Na2-binding site, mutations of residues involved in coordinating this site, including C95P, A99P, and F415P, completely abolished DddT transport activity (Fig. 4D). Other mutations at the Na2 site, specifically V98P and T419A, retained residual transport activity but led to a notable reduction in transport activity under varying sodium gradient conditions (Fig. 4C,D; Appendix Table S2). Interestingly, the T418A mutant exhibited a higher *V*_max_ relative to wild-type DddT under conditions of a high sodium gradient, whereas it showed reduced transport activity under low sodium gradient conditions (Fig. 4D; Appendix Table S2). This variation suggests that the T418A mutation increases the dependence on a high sodium gradient while compromising transport efficiency when sodium availability is limited. Collectively, these findings highlight the critical roles of the Na1 and Na2-binding sites in enabling efficient DMSP transport.

### The role of the two Na^+^ ions

Na1 was observed in the C_c_S and C_e_ states, but not in the C_c_, C_c_-K^+^, or C_i_ states (Fig. 3A). Structurally, the Na1 site, strategically located between TMH3, TMH7, and TMH10, appears to help stabilize these three helices, which can move apart to form the inward-facing cavity in the C_i_ state. Since the key residues involved in Na2 binding are also located on these three helices, binding at Na1 and Na2 may affect each other. As described above, Na1 exhibits less stable binding. The fact that Na1 can still be identified in our cryo-EM structures is probably due to the high Na^+^ concentration present on both the periplasmic and cytoplasmic sides in our sample preparation, which promotes Na1 occupancy. During the actual transport cycle, however, the cytoplasmic Na^+^ concentration is much lower, likely leading to Na1 dissociation and consequently affecting Na2 binding. For Na2, as analyzed above, the binding of DMSP and Na^+^ as well as the conformational changes of the hinge region are coupled, and alteration of one component inevitably influences the other two. From the structural details, Na2 and DMSP bind to opposite sides of the TMH3 hinge region. Na2 appears to be directly involved in this coupling. The peptide bond between Val98 and Ala99 in the TMH3 hinge participates in the binding of both DMSP and Na2. The carbonyl oxygen of this peptide bond coordinates Na2, while the amide nitrogen is likely involved in DMSP binding (Fig. 3E). In the C_c_ state, significant conformational rearrangements in the TMH3 hinge region led to a flip of this peptide bond so that the carbonyl oxygen no longer faces the Na2 site and the amide nitrogen no longer faces the DMSP site (Fig. 3C,E). Based on these observations, we propose a cooperative function of Na1 and Na2. The relatively weak coordination of Na1 is crucial for DddT to exploit the Na^+^ gradient. Moreover, because both ions are coordinated by TMH3, TMH7, and TMH10, their interplay enables Na2 to effectively harness this energy. In turn, Na2 directly contributes to the coupled process of DMSP binding and hinge conformational changes, thereby enabling substrate transport.

To further validate that both Na1 and Na2 are essential for DddT-catalyzed DMSP transport, we performed solid-supported membrane (SSM) electrophysiology assays. The results revealed that DddT mediates DMSP uptake with a stoichiometry of approximately two Na⁺ ions per DMSP molecule (Appendix Fig. S18), which supports our analyses identifying two distinct Na⁺-binding sites.

### The DMSP transport mechanism

**Figure 5:**
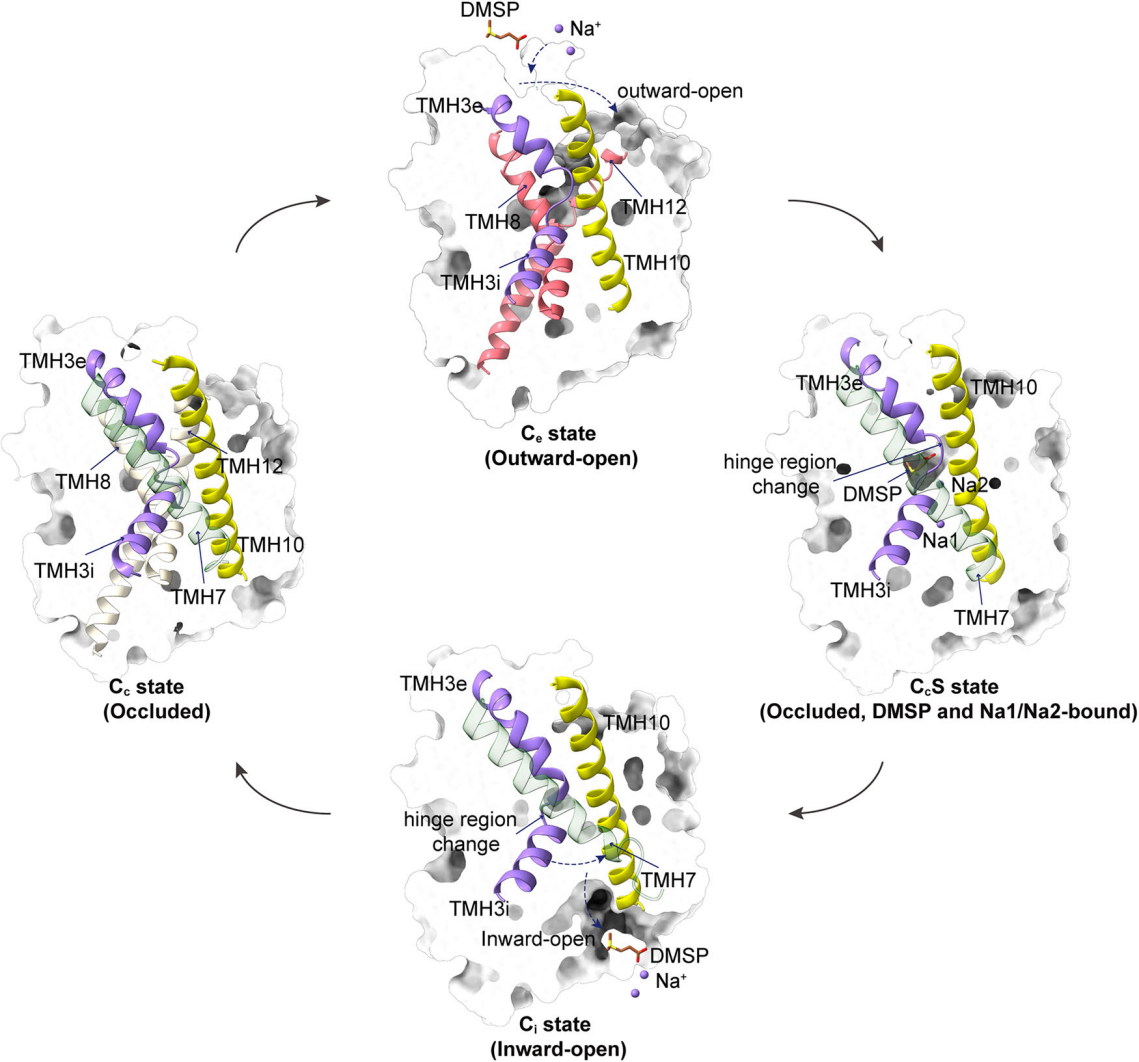
Proposed cartoon diagram of the transport mechanism of DddT. TMH3 and TMH10 in the C_c_, C_e_, C_c_S, and C_i_ states are highlighted in purple and yellow, respectively. TMH7 in the C_c_, C_c_S, and C_i_ states is shown in green. TMH8 and TMH12 in the C_c_ state are colored beige, and TMH8 and TMH12 in the C_e_ state are shown in red. Sodium ions are shown as purple spheres. All DddT conformational states were determined by cryo-EM in this study.

Based on structural analysis and biochemical validation, we proposed the DMSP transport process and mechanism of DddT (Fig. 5). When DMSP is present in an environment with an appropriate sodium gradient, DddT transitions to the C_e_ state, allowing for the simultaneous entry of DMSP and sodium ions. The entry of DMSP and sodium ions is facilitated by the widening of the angle between TMH8 and TMH10. Subsequently, sodium ions rapidly bind to the Na1 and Na2 sites, the DMSP molecule binds to the TMH3 hinge region, and the TMH3 hinge undergoes a conformational change, leading DddT to transition into the C_c_S state. Due to the Na^+^ gradient across the membrane and the relatively low intracellular Na^+^ concentration, the sodium ion at the Na1 site dissociates, resulting in instability of the TMH3, TMH7, and TMH10 helices and further destabilizing Na2 binding. Because Na2, DMSP, and the TMH3 hinge region are coupled, dissociation of Na2 triggers the release of DMSP and conformational changes in the TMH3 hinge. During this process, DMSP passes through the TMH3 hinge region, and the release of Na^+^ ultimately drives the transition of DddT into the C_i_ state. Concurrently, a cytoplasm-facing cavity gradually forms to allow DMSP entry into the cytoplasm. Once DMSP and sodium ions have entered the cytoplasm, in the absence of mutations that stabilize the inward-facing conformations, wild-type DddT reverts to a state ready for the next transport cycle. While DddT, with its newly identified Na1 site, mediates a transport process that differs from BetP, the Na2 site plays a conserved and central functional role in both systems.

### A broad implication of the transport mechanism akin to DddT

**Figure 6:**
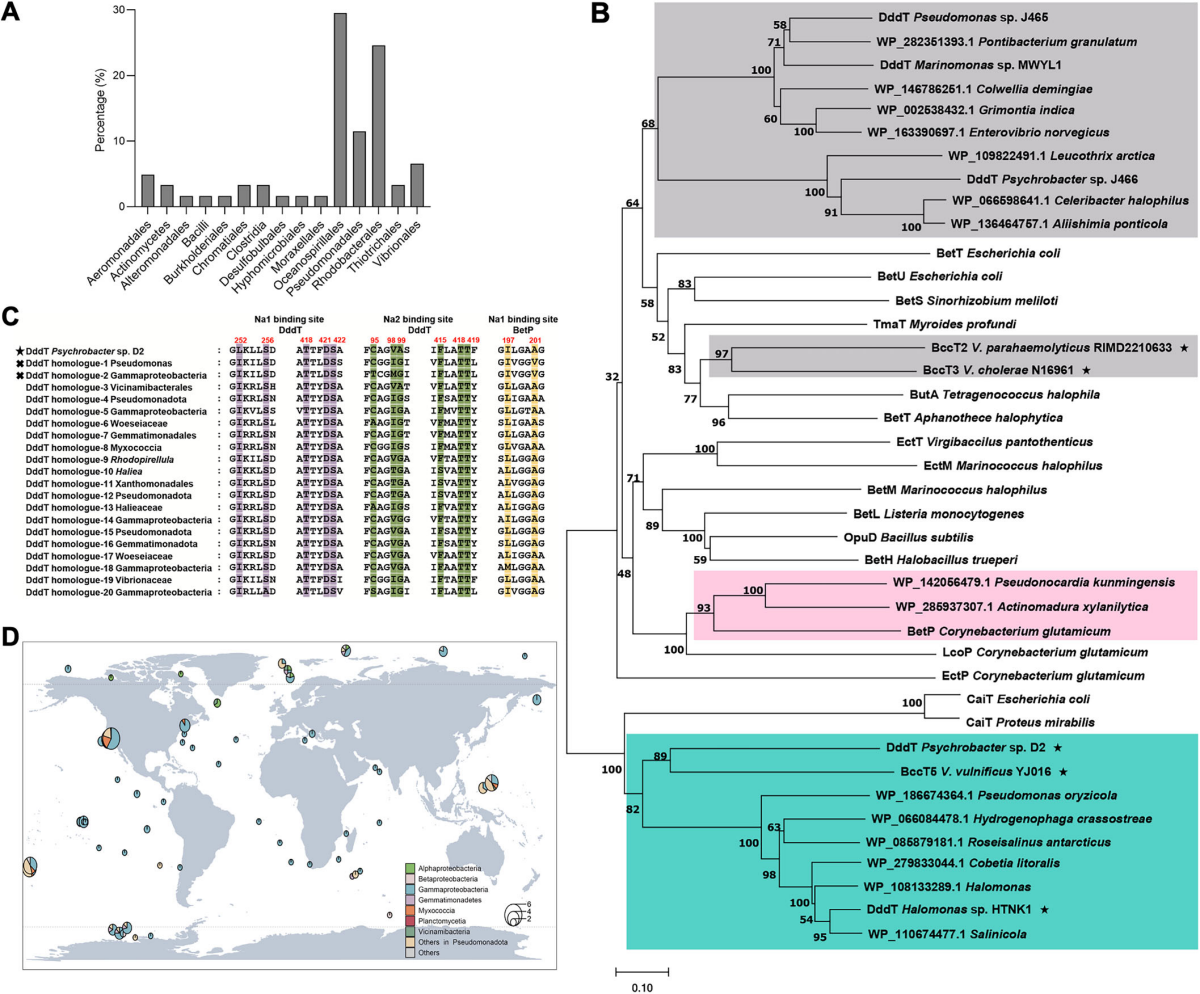
The broad significance of the DddT transport mechanism. (**A**) The distribution of identified DddT homologues whose genes clustered with the DMSP lyase genes *dddD/dddX* in bacteria. (**B**) Phylogenetic analysis towards experimentally confirmed functional DddTs (Gregory et al, 2021; Todd et al, 2010), identified DddT homologues clustered with the DMSP cleavage enzymes DddD/DddX(Curson et al, 2010), and other BCCT family transporters (Ziegler et al, 2010). DddT homologues grouped with BetP are highlighted with gray and pink backgrounds. The DddT homologues with two hydrophilic residues at the BetP Na1-binding site are shown in pink, while those with one hydrophilic residue are shown in gray. DddT homologues grouped with *P*. sp. *D2* DddT, which have no hydrophilic residues at the BetP Na1-binding site, are highlighted with a green background. The experimentally confirmed functional DddTs are marked with ★. (**C**) Multi-sequence alignment of *P*. sp. D2 DddT and 20 randomly selected DddT homologues identified from metagenomic analysis. Key residues around the sodium-binding site are marked out. The experimentally confirmed functional DddTs are marked with ★, while the DddT homologues not involved in DMSP transport are marked with **×**. The 20 sequences were randomly sampled from each major clade after taxonomic classification to ensure balanced representation. (**D**) The geographic and species distribution of the 411 DddT homologues from metagenome data. Source data are available online for this figure.

As one of the primary transporters of DMSP, DddT is distributed in numerous DMSP-metabolizing bacterial strains (Sun et al, 2012). Therefore, we first analyzed whether DddT from different bacterial strains employs the same transport mechanism. The BCCT-family transporters exhibit a high sequence similarity, and it is difficult to distinguish between different transporters for various substrates based solely on sequence similarity analysis. Therefore, we conducted gene cluster analysis. Using the representative bacterial genomes obtained from GenBank, 61 *dddT* genes were clustered with the DMSP lyase genes *dddD/dddX*. The corresponding DddT proteins were mainly distributed among gamma-proteobacteria, with Oceanospirillales and Pseudomonadales comprising a large proportion, followed by Alphaproteobacteria, with most belonging to the Rhodobacterales (Fig. 6A). This distribution pattern suggests a prominent role of DddT in various bacterial groups, particularly those associated with marine and aquatic environments.

**Figure EV3:**
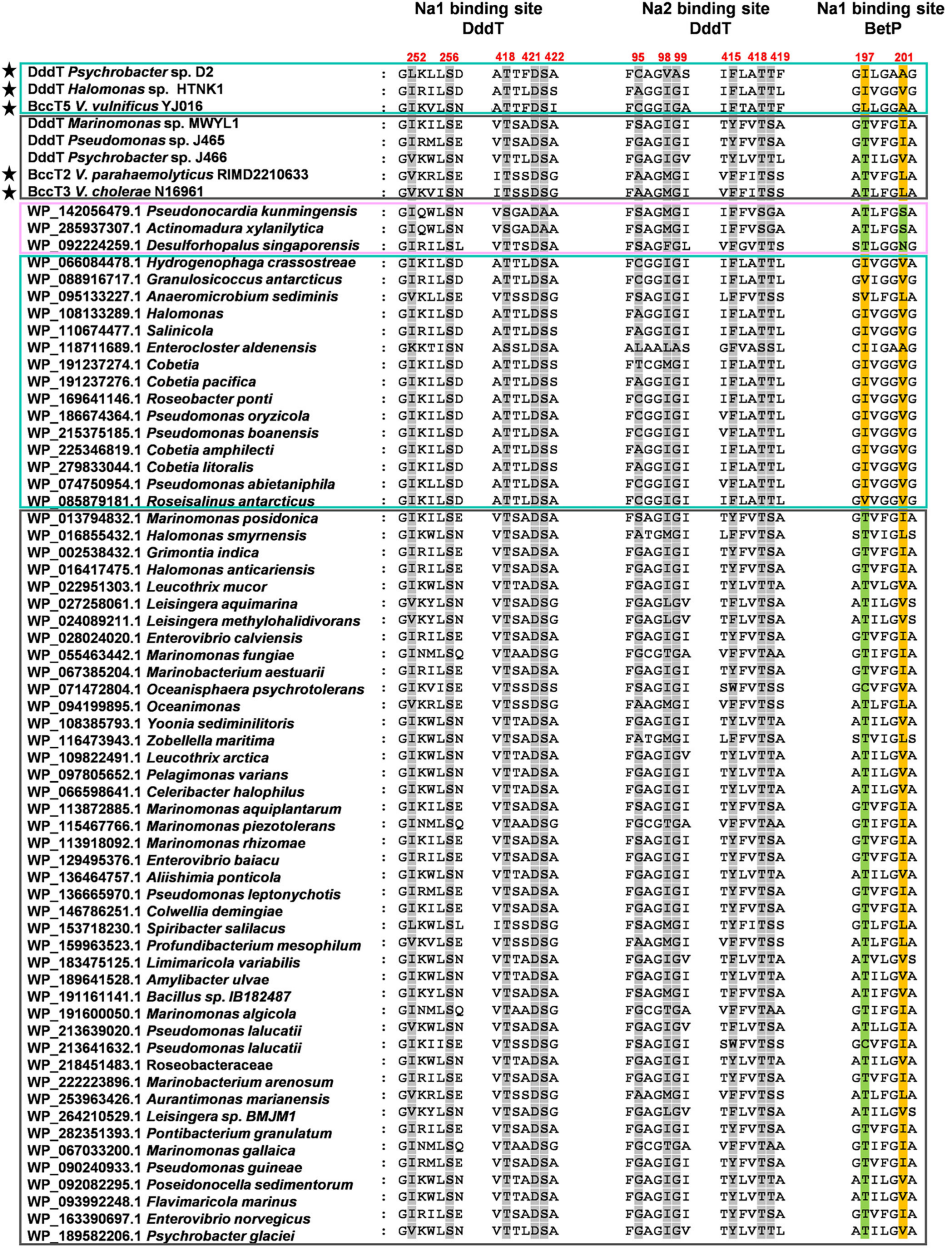
Multi-sequence alignment of the experimentally ratified DddT proteins and 61 identified DddT homologues whose genes are linked to the DMSP lyase genes DddD/DddX. Key residues around the sodium-binding site are marked out. Based on the hydrophilicity and hydrophobicity of key residues at the Na1-binding site of BetP, sequences are divided into three categories. In the gray box, there is one hydrophilic residue and one hydrophobic residue. In the blue box, both residues are hydrophilic, and in the pink box, both residues are hydrophobic. The functional DddTs are marked with ★. Hydrophilic residues are colored green, and hydrophobic residues are colored orange. Source data are available online for this figure.

Systematic phylogenetic analysis revealed that these DddT proteins are grouped into distinct phylogenetic branches within the BCCT family. Some of the DddT proteins were closer to the branch where BetP was located, whereas those grouped with DddT from *P*. sp. D2 were closer to the Na^+^-independent substrate/product antiporter CaiT (Fig. 6B). This suggests that although these DddT proteins likely transport DMSP, there may be variations in their transport process. This was supported by a comparative analysis of the Na^+^-binding sites of these DddT proteins. We found that, in addition to the typical Na2-binding site, there were differences in the Na1-binding site depending on the clustering. The corresponding residues at the BetP Na1-binding site of the DddT proteins clustered with DddT from *P*. sp. D2 were all hydrophobic residues and unable to bind Na, whereas the residues at the DddT Na1 site were relatively conserved (Fig. EV3). This indicated that they probably adopted similar Na^+^-binding sites as DddT from *P*. sp. D2. In contrast, the corresponding residues at the BetP Na1-binding site of DddT proteins closer to the branch where BetP was located had at least one hydrophilic residue. This suggests that they may contain a similar Na1-binding site and possess a transport mechanism similar to that of BetP.

**Figure EV4:**
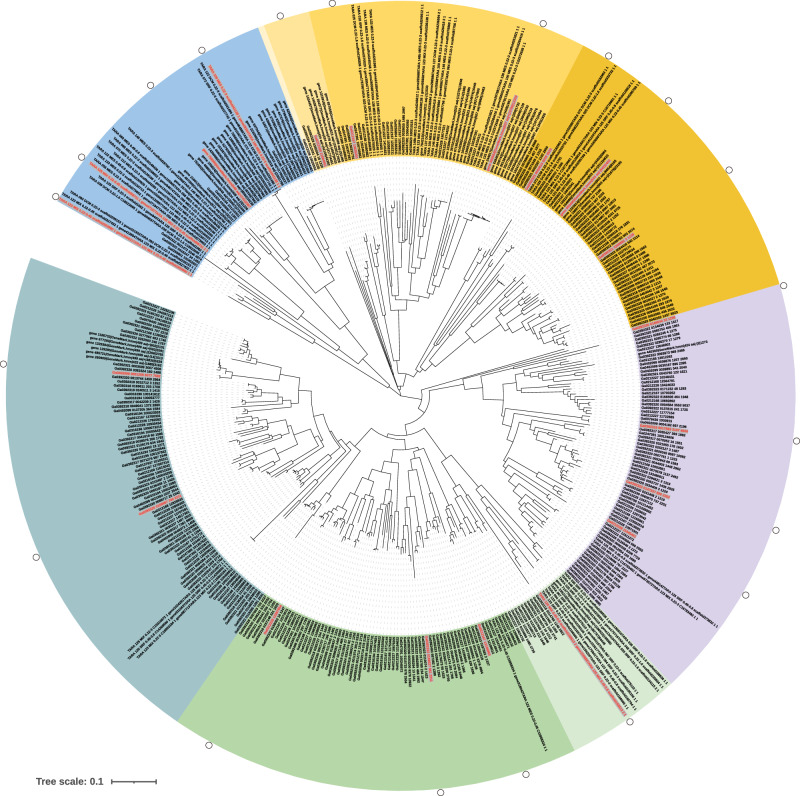
Phylogenetic analysis of selected 411 DddT homologues from metagenome data. Branches with different colors represent distinct clades in the phylogenetic tree. The sequences highlighted by shading and shown in red within the circle correspond to 20 randomly selected sequences from different branches of the phylogenetic tree, which were used for the multiple sequence alignment in Fig. 6C. Source data are available online for this figure.

Subsequently, we examined DddT-like BCCT-family transporters predicted from metagenomic data obtained from sediment, polar ocean, and Tara Ocean samples. Using the sequence length as a criterion, we identified 645 sequences. Among these, 411 sequences shared similarities with DddT from *P*. sp. D2, which displayed hydrophobic residues at positions corresponding to the BetP Na1-binding site (Figs. 6C and EV4). DddT homologues are widely dispersed among the metagenomic samples in the oceans (Fig. 6D). To assess their functionality, two of these homologues were selected for DMSP transport activity analysis. However, none of them demonstrated DMSP transport activity (Appendix Fig. S19), indicating the presence of numerous BCCT-family transporters that do not transport DMSP but may employ a transport mechanism akin to DddT from *P*. sp. D2. These findings suggest that, in BCCT-family transporters, different substrates can induce variations in the transport process, while similar mechanisms can accommodate the transport of diverse substrates. These observations highlight the complexity and versatility of transport processes within the BCCT family, suggesting that the relationship between substrate specificity and transport mechanism is more nuanced than previously thought.

In summary, this study elucidates the structural basis for bacterial absorption of the important sulfur-containing organic compound DMSP via DddT. These findings deepen our understanding of DMSP transport and offer new insights into the mechanisms of the BCCT-family transporters, emphasizing the specific role of sodium.

## Methods

**Table:** Reagents and tools table

Reagent/resource	Reference or source	Identifier or catalog number
**Experimental models**		
*Psychrobacter* sp. D2	Zhang Laboratory	NCBI: JACDXZ000000000
Δ*dddT*	This study	
Δ*dddT*/pBBRMCS2-*dddT*	This study	
*E. coli* WM3064	Dehio and Meyer, 1997	
*E. coli* DH5α	Vazyme Biotech Company, China	Cat # C502-02
*E. coli* C43 (DE3)	WEIDI Biotechnology Company, China	Cat # EC1040
**Recombinant DNA**		
pK18*mobsacB*-Ery	Wang et al, 2015b	
pK18*mobsacB*-Ery- *dddT*	This study	
pET-22b-*dddT*	This study	
pET-22b-Bba-*dddT*	This study	
pBBR1MCS2	Kovach et al, 1995	
pBBR1MCS2-*dddT*	This study	
**Antibodies**		
Anti-His Tag Monoclonal antibody	Solarbio, China	Cat # K200060M
Anti-GAPDH rabbit polyclonal antibody	Sangon Biotech, China	Cat # D110016
HRP-labeled Goat Anti- mouse IgG(H + L)	Beyotime, China	Cat # A0350
HRP-labeled Goat Anti-Rabbit IgG(H + L)	Beyotime, China	Cat # A0352
**Oligonucleotides and other sequence-based reagents**		
PCR primers	This study	Appendix Table S3
**Chemicals, enzymes, and other reagents**		
FastPfu DNA Polymerase	TransGenBiotech, China	Cat # AP221-01
In-Fusion Snap Assembly Maser Mix	TaKaRa, Japan	Cat # 638947
Pierce BCA Protein Assay Kit	Thermo Fisher, USA	Cat # 23227
**Software**		
cryoSPARC	Punjani et al, 2017	
UCSF Chimera	Pettersen et al, 2004	
COOT	Casañal et al, 2020	
Phenix	Liebschner et al, 2019	
MolProbity 4	Williams et al, 2018	
ChimeraX	Pettersen et al, 2021	
**Other**		

### Bacterial strains and growth conditions

*P*. sp. D2 and its mutants and supplemental strains were cultured in marine broth 2216E medium or the basal medium (Appendix Table S4) supplemented with 6 mM DMSP, glucose, or succinate as the carbon source at 20 °C. *E. coli* strains DH5α and C43 (DE3) were cultured in lysogeny broth (LB) medium, whereas *E. coli* strain WM3064 (Dehio and Meyer, 1997) was supplemented with 0.3 mM diaminopimelic acid. All the *E. coli* strains were cultured at 37 °C.

### Gene knockout

The *dddT* gene of *P*. sp. D2 was knocked out using the suicide plasmid, pK18*mobsacB*-Ery (Wang et al, 2015b), through homologous recombination. The upstream and downstream homology arm sequences of *dddT* were PCR-amplified from *P*. sp. D2 genomic DNA using two pairs of primers, *dddT*-Up and *dddT*-Down, and the *dddT*-Up-Down homology arm fragment was obtained using the In-Fusion HD Plus Cloning Kit (Takara, USA) via gene splicing by overlap extension PCR (SOE PCR). The *dddT*-Up-Down homology arm fragment was seamlessly connected with linearized pK18*mobsacB*-Ery to construct a marker-free knockout plasmid of the *dddT* gene, which was named pK18*mobsacB*-Ery-*dddT*. The plasmid pK18*mobsacB*-Ery-*dddT* was transformed into *E. coli* WM3064 as the donor strain, whereas *P*. sp. D2 was used as the recipient strain for intergeneric conjugation.

For screening and validation of the first homologous recombination mutant, surviving colonies were selected on marine 2216E agar plates containing 25 μg/mL erythromycin and validated by PCR amplification with primers *dddT*-SF/*dddT*-SR to obtain colonies for which the pK18*mobsacB*-Ery-*dddT* plasmid was inserted into the genome of *P*. sp. D2. The mutant bacteria that underwent the first homologous recombination were cultured in Marine Broth 2216E medium and plated on Marine 2216E agar plates containing 10% (w/v) sucrose. The colonies grown on the plate were selected and screened using PCR verification with *dddT*-LF/*dddT*-LR primers to identify the monoclonal strains that had undergone the second homologous recombination. The final *dddT* mutant underwent a second homologous recombination and was sensitive to erythromycin.

Using the genomic DNA of *P*. sp. D2 as a template, *dddT* and its promoter were amplified using *dddT*-pBBR-F/*dddT*-pBBR-R primers. The PCR fragment was seamlessly ligated to linearized pBBR1MCS2 (Kovach et al, 1995) using the In-Fusion HD Plus Cloning Kit. After transformation into *Escherichia coli* DH5α, a single clone was selected and validated by sequencing to produce the pBBR1MCS2-*dddT* plasmid. The pBBR1MCS2-*dddT* plasmid was transformed into *E. coli* WM3064 and mobilized into the Δ*dddT* mutant by intergeneric conjugation. The screening plate was marine 2216E agar plates containing 80 μg/mL kanamycin, and PCR was used to verify the correctness of the complemented mutant. The same method was used to mobilize the empty vector pBBR1MCS2 into Δ*dddT*. Primers used in this study are listed in Appendix Table S3.

### DMSP utilization assay

Cells were grown in Marine Broth 2216E medium, harvested by centrifugation, and washed three times with sterile artificial seawater. The washed cells were diluted to the same density of OD_600_ ≈ 2.0, and 1% (v/v) cells were inoculated into the basal medium with 6 mM DMSP. The seeded cells were cultured at 20 °C in the dark, and samples were collected at various times. The growth of the bacteria was determined by measuring the OD_600_ using a spectrophotometer V-550 (Jasco Corporation, Japan), and the utilization of DMSP was determined by liquid chromatography after filtering with a 0.22-μm filter. For the DMS production assay, the seeded cells were cultured at 20 °C in a sealed headspace vial for 21 h, and the DMS production was detected using a gas chromatograph (GC-2030, Shimadzu, Japan) equipped with a flame photometric detector(Liu et al, 2018).

### Plasmid construction for activity assay

The genomic DNA of *P*. sp. D2 was used as a template to amplify *dddT* using 22b-Bba-*dddT*-F/22b-Bba-*dddT*-R primers. The constitutive promoter BBa-J23111 was cloned upstream of *dddT*, resulting in a fragment containing *dddT* with the BBa-J23111 promoter of *E. coli*. This fragment was then inserted into the NdeI/XhoI restriction sites of pET-22b to generate the *dddT* constitutive expression plasmid pET-22b-Bba-*dddT*. The plasmids pET-22b-Bba and pET-22b-Bba-*dddT* were transferred into *E. coli* DH5α, resulting in the strains Ec-CK and Ec-DddT.

### Transport assays

The *P*. sp. D2 and Δ*dddT* strains were grown at 20 °C in the marine broth 2216E medium to an OD_600_ of 0.8, whereas the complemented strain (Δ*dddT*/pBBR1MCS2-*dddT*) was cultivated in the same medium supplemented with 50 μg/mL kanamycin. Cells were diluted to the same density for uptake measurements. After adding 5 mM DMSP for 2 h at 20 °C in the dark, the cells were harvested by centrifugation, washed three times with sterile artificial seawater, and resuspended in the same volume to determine intracellular uptake of DMSP.

Ec-DddT and its mutants were cultivated at 37 °C in Luria-Bertani (LB) medium (50 μg/mL ampicillin) to an OD_600_ of 1.0. Cultured cells were harvested by centrifugation at 8000 rpm for 2 min. The cell pellets were washed three times with 25 mM KPi buffer (pH 7.5), each time resuspending the cells thoroughly and centrifuging to remove the supernatant. After the final wash, the cells were resuspended in the same KPi buffer supplemented with 20 mM glucose. The cell suspensions of both wild-type Ec-DddT and its mutants were adjusted to the same final volume and normalized to an OD_600_ of 1.0.

There were some differences in the measurement of transport activity between saturated and gradient NaCl conditions. To determine DMSP transport activity under saturated NaCl and DMSP conditions, NaCl was added to the cell suspension to a final concentration of 200 mM. For assays under gradient NaCl concentrations with saturated DMSP, varying NaCl concentrations were supplemented. Based on the NaCl concentration added, an appropriate amount of KCl was supplemented to maintain the system osmolarity at 800 mOsm/kg. Subsequent steps for the measurement of transport activity were identical. The mixtures were gently mixed and incubated at 37 °C for 3 min. DMSP was then added to a final concentration of 250 mM, followed by incubation at 37 °C. DMSP uptake was terminated 10 min after addition by collecting cells via centrifugation. Cells were washed three times with 200 mM KPi buffer to remove extracellular DMSP and finally resuspended in an equal volume of 200 mM KPi buffer for activity determination.

All samples were then assayed on a gas chromatograph (GC-2030, Shimadzu, Japan) equipped with a flame photometric detector for DMS production (Liu et al, 2018), from which DMSP uptake was calculated according to the standard curve.

### Western blot detection of protein levels

Protein levels in cell extracts were analyzed using western blot. The DddT and its mutants, with C-terminal His-tag expressed on the constitutive expression plasmid pET-22b-Bba, were detected by anti-His antibodies. Ec-DddT and its mutants were cultivated at 37 °C in LB medium (50 μg/mL ampicillin) to an OD_600_ of 1.0. The cells were harvested and washed three times in 25 mM KPi buffer (pH 7.5), resuspended, and diluted in the same buffer to achieve an OD_600_ of approximately 1.0. The cells were then disrupted by a high-pressure crusher. The cell lysate was centrifuged at 20,000×*g* for 10 min, and the total protein content in the supernatant was quantified using the BCA assay. The proteins (10 μg) were separated through SDS-PAGE. Subsequently, the separated proteins were electrotransferred onto a PVDF membrane (Invitrogen, Carlsbad, CA). The membrane was blocked with QuickBlock solution (Beyotime, China) at 25 °C for 30 min and incubated with a mouse-derived anti-His monoclonal IgG1 antibody (Solarbio, China) at a 1:1000 dilution, maintained under gentle rocking at 25 °C for 2-4 h. The membrane was washed three times in TBST (Tris-Buffered Saline with Tween-20) before being incubated with HRP-conjugated goat anti-mouse IgG (Beyotime, China) secondary antibody at a 1:2000 dilution in TBST at 25 °C for 1 h. After three additional TBST washes, protein signals were visualized using BeyoECL Plus (Beyotime, China). Glyceraldehyde 3-phosphate dehydrogenase (GAPDH) was detected with an anti-GAPDH monoclonal antibody IgG1 (Solarbio, China) as the control. The expression levels of DddT and its mutants in the cells were normalized by Western blot using Image-Pro Plus software.

### Gene cloning, point mutation, protein expression, and purification

The *dddT* gene was amplified using PCR from the genomic DNA of *P*. sp D2 and inserted into the NdeI/XhoI restriction sites of the pET-22b vector containing a C-terminal His tag. The G101D point mutation in DddT was introduced using polymerase chain reaction-based methods. The constructed vector was transformed into *E. coli* C43 (DE3) cells for protein expression. Cells were grown at 37 °C in LB medium supplemented with 100 μg/mL ampicillin to OD_600_ of 0.8-1.0 and induction was initiated with 0.5 mM isopropyl-β-D-thiogalactopyranoside (IPTG) at 18 °C for 16 h. Cells were harvested at 4 °C by centrifugation and lysed in lysis buffer (40 mM Tris-HCl, 200 mM NaCl, 10% glycerol, 2 mM EDTA, 2 mM DTT, 0.1 mM PMSF, PH 8.0). Membranes were isolated from disrupted cells and solubilized with 2% n-dodecyl-β-D-maltopyranoside (DDM) in membrane solubilization buffer (40 mM Tris-HCl, 200 mM NaCl, 10% glycerol, 0.5 mM DTT, 0.1 mM PMSF, pH 8.0). The proteins were then purified by affinity chromatography on a Ni^2+^-NTA column (Qiagen, Germany), washed with buffer containing 50 mM imidazole, 40 mM Tris-HCl (pH 8.0), 200 mM NaCl, 5% glycerol, 0.5 mM DTT, and 0.05% DDM, and eluted with buffer containing 300 mM imidazole, 40 mM Tris-HCl (pH 8.0), 200 mM NaCl, 5% glycerol, 0.5 mM DTT, and 0.05% DDM. Purified DddT was fractionated by gel filtration on a Superdex-200 column (GE Healthcare, USA) in buffer containing 20 mM Tris-HCl (pH 8.0), 200 mM NaCl, and 0.02% DDM.

### Na^+^/DMSP stoichiometry assay using solid-supported membrane (SSM) electrophysiology

Proteoliposomes were prepared by dissolving *E. coli* total lipid extract (12.5 mg/mL) in chloroform, drying under nitrogen, lyophilizing for 6 h, and resuspending in the appropriate B buffer (30 mM HEPES, 2 mM MgCl₂, 50 mM KCl, 50 mM NaCl, pH 7.5) supplemented with 40 mM DDM. Lipids were solubilized overnight at 4 °C with gentle agitation. The purified protein was exchanged into the same B buffer, then mixed with purified protein to achieve final concentrations of 4 mg/mL lipid and 0.8 mg/mL protein. Empty liposomes were prepared in parallel using identical lipid and buffer compositions without protein. Detergent was removed using Bio-Beads SM-2 overnight at 4 °C, yielding vesicle suspension.

Three independent proteoliposome batches were generated corresponding to B buffers B1, B2, and B3, containing 100, 200, and 50 μM DMSP, respectively. Empty liposomes were prepared in parallel in the same buffers. Proteoliposomes were sonicated using a bath sonicator (JP-010, Skymen, China) for 10 s, repeated three times, prior to use. Electrophysiological recordings were performed using a SURFE^2^R N1 solid-supported membrane-based electrophysiology system (Nanion Technologies). N1 Single Sensors (Nanion Technologies; catalog no. 2-03-35002-000) were incubated with 50 μL of 0.5 mM 1-octadecanethiol in isopropanol for 30 min at room temperature. Sensors were washed twice with isopropanol, rinsed three times with distilled water, and air-dried. Subsequently, 1.5 μL of 7.5 μg/μL 1,2-diphytanoyl-sn-glycero-3-phosphocholine in n-decane was applied, followed by the addition of 50 μL of B buffer. Proteoliposomes (10 μL) were then applied to the sensors, which were centrifuged at 2000× *g* for 30 min at 25 °C to promote adsorption of the proteoliposomes onto the sensor surface. Transport currents were recorded using the B + BAB protocol, in which each sensor was perfused with the corresponding B buffer prior to each measurement, followed by rapid application of the paired A buffer (30 mM HEPES, 2 mM MgCl₂, 100 mM NaCl, and 50 or 200 μM DMSP), and subsequently re-perfused with the corresponding B buffer before the next measurement. Measurements were performed in triplicate.

Peak currents were normalized to the signals recorded from empty-liposome sensors under identical buffer-switch conditions to correct for non-specific capacitive contributions. Current amplitudes were plotted against the calculated chemical potential ratio Δμ(DMSP)/Δμ(Na^+^), yielding three data points corresponding to −1, −2, and +2 for B1/A1, B2/A2, and B3/A3 pairs, respectively. Linear regression of normalized peak currents versus chemical potential ratio was used to determine the apparent Na^+^/DMSP coupling stoichiometry.

### Cryo-EM sample preparation and data collection

The purified DddT protein samples were concentrated at 4 °C to 5 mg/mL in 20 mM Tris-HCl (pH 8.0), 200 mM NaCl/KCl, and 0.02% DDM. A 4.0-μL aliquot of the samples was applied onto a freshly glow-discharged holey carbon grid (Quantifoil Au R2/1, 200 mesh) with a continuous carbon support. The grids were plunge frozen in liquid ethane using an FEI Vitrobot Mark IV (ThermoFisher Scientific) at 4 °C and >90% humidity with 2 s blot time and −1 blot force. The grids were loaded into a 300 kV Titan Krios G3i microscope (Thermo Fisher) equipped with a K3 BioQuantum direct electron detector (Gatan, USA) for data acquisition. A total of 5747 movies for C_c_ was collected at a total dose for a stack of ~64 e^−^ Å^−2^ in a defocus range of −1.6 to 2.2 μm. A total of 4845 movies for C_c_S, 6804 movies for C_c_-K^+^, 4587 movies for C_e_, and 6,174 movies for C_i_ were collected at a total dose for a stack of ~60 e^−^ Å^−2^ in a defocus range of −0.8 to 1.6 μm. For C_c_, a super-resolution mode was used at a nominal magnification of ×81,000 corresponding to a pixel size of 0.53 Å. For C_c_S, C_c_-K^+^, C_e_, and C_i_, a super-resolution mode was used at a nominal magnification of ×130,000 corresponding to a pixel size of 0.97 Å.

### Cryo-EM image processing

Data processing was performed using cryoSPARC Software (Punjani et al, 2017). After patch-motion and CTF correction were performed, particles were picked using the Blob picking algorithm of cryoSPARC. The particles were then subjected to several rounds of 2D classification. These runs yielded particles for analysis that were subjected to ab initio reconstructions. After homogeneous refinement and 3D non-uniform refinement, global (per-group) CTF refinement, and local (per-particle) CTF refinement were performed. The resolution was estimated using the gold standard Fourier shell correlation (FSC) of the 0.143 criteria in cryoSPARC. The overall resolutions of the maps of C_c_, C_c_S, C_c_-K^+^, C_e_, and C_i_ were 2.8, 2.52, 3.18, 2.66, and 3.29 Å, respectively. Details of the cryo-EM data processing and associated parameters are provided in Appendix Table S1, Appendix Figs. S6, S8, S10, S13, and S15.

### Model building and refinement

AlphaFold2 was initially used to predict DddT structure. It was then docked into the resolution cryo-EM map using UCSF Chimera (Pettersen et al, 2004). The DddT model in the C_c_ state was manually rebuilt based on the cryo-EM density using COOT (Casañal et al, 2020) and then refined using Phenix 1.16.3549 (Liebschner et al, 2019). The atomic model of DddT in the C_c_S, C_c_-K^+^, C_e_, and Ci states was built using a method similar to that of DddT, but using DddT as a reference starting model. The geometrical restraint of DMSP was generated from the Grade Web Server. MolProbity 4 (Williams et al, 2018) was used to evaluate the structure geometries. Images were rendered using Chimera and ChimeraX (Pettersen et al, 2021).

### Bioinformatics

Using the representative bacterial genomes obtained from GenBank, homologues of DddT, DddD, and DddX were first identified by setting the E-value to <1E^−50^ and similarity to >35% compared to functional DddT (*P*. sp. D2) (this study) and DddX (*P*. sp. D2) (Li et al, 2021), and DddD (*Marinomonas* sp. MWYL1) (Todd et al, 2007) using blastp. DddD homologues were further screened based on protein sequence length, retaining only those with sequences exceeding 600 amino acids. Subsequently, the genomic positions of genes corresponding to the selected protein homologues were analyzed. Only the protein sequences corresponding to *dddT* genes that were less than five genes away from *dddD*/*dddX* genes were retained. Finally, 61 putative DddT proteins clustered with DMSP cleavage enzymes DddD or DddX were obtained.

For metagenomic analysis, a hidden Markov model (HMM) was constructed using two functional DddT sequences obtained from *P*. sp. D2 and *H*. sp. HTNK1. This model was subsequently employed to search for DddT homologues from sediment samples (https://img.jgi.doe.gov/cgi-bin/m/main.cgi) and polar ocean samples (NCBI BioProject accession no. PRJNA588686), and Tara Ocean samples (https://tara-oceans.mio.osupytheas.fr/ocean-gene-atlas/). Hmmsearch (http://hmmer.org) was performed with a cut-off value of <1E^-50^, a percentage identity >35%, and a sequence criterion of >400 amino acids. Phylogenetic trees were constructed using the maximum likelihood method in MEGA, version 7.0. Sequence alignment was performed using the BioEdit Sequence Alignment Editor with the ClustalW Multiple Alignment method. Visualization of the geographical and species distribution was performed using R software.

## Supplementary information

Peer Review File

Appendix

Source data Fig. 1

Source data Fig. 3

Source data Fig. 4

Source data Fig. 6

Figure EV3 Source Data

Figure EV4 Source Data

Appendix Figure Source Data

Expanded View Figures

## Data Availability

The atomic coordinates and cryo-EM maps in this study for the DddT in closed substrate-free conformation (PDB entry 21FF; EMD-67623), DddT in closed DMSP-bound conformation (PDB entry 21FI; EMD-67626), DddT in closed substrate-free conformation in the presence of potassium ions and dimethylsulfoniopropionate (PDB entry 21FJ; EMD-67627), DddT G101D in substrate-free outward open conformation (PDB entry 21FH; EMD-67625), and DddT G101D in substrate-free inward open conformation (PDB entry 21FK; EMD-67628) have been deposited in the Protein Data Bank (http://www.rcsb.org) and the Electron Microscopy Data Bank (https://www.ebi.ac.uk/pdbe/emdb/). The source data of this paper are collected in the following database record: biostudies:S-SCDT-10_1038-S44318-026-00798-w.
