# Supplementary material for: Structural insights into bacterial dimethylsulfoniopropionate import by BCCT-family transporters
Source: EMBO J. 2026 May 8;45(12):4299–320. doi: 10.1038/s44318-026-00798-w (PMC13270143; doi:10.1038/s44318-026-00798-w)
Supplement: Supplementary file 2 — Appendix [file 44318_2026_798_MOESM2_ESM.pdf]

Structural insights into bacterial dimethylsulfoniopropionate import by BCCT-family transporters

Yu-Zhong Zhang[1,2,3#*], Wen-Jing Zhu[1,2,3#], Kang Li[1,3#], Hai-Tao Ding[4], Motoyuki Hattori[5], Shuaimeng Liu[5], Chang Ge[2], Qi-Long Qin[2,3], Zhao-Jie Teng[2], Ning-Hua Liu[2], Hai-Yan Cao[1,3], Chun-Yang Li[1,3], Xiu-Lan Chen[2,3], Qing-Tao Shen[6], Jonathan D Todd[1,7], Lu-Ning Liu[1,8*], Peng Wang[1,3*]

Table of Contents Appendix Figures and Tables:

Appendix Figures:

Appendix Figure S1……………………………………………………………………………2
Appendix Figure S2……………………………………………………………………………3
Appendix Figure S3……………………………………………………………………………4
Appendix Figure S4……………………………………………………………………………5
Appendix Figure S5……………………………………………………………………………6
Appendix Figure S6……………………………………………………………………………7
Appendix Figure S7……………………………………………………………………………8
Appendix Figure S8……………………………………………………………………………9
Appendix Figure S9……………………………………………………………………………10
Appendix Figure S10……………………………………………………………………………11
Appendix Figure S11……………………………………………………………………………12
Appendix Figure S12……………………………………………………………………………13
Appendix Figure S13……………………………………………………………………………14
Appendix Figure S14……………………………………………………………………………15
Appendix Figure S15……………………………………………………………………………16
Appendix Figure S16……………………………………………………………………………17
Appendix Figure S17……………………………………………………………………………18
Appendix Figure S18……………………………………………………………………………19
Appendix Figure S19……………………………………………………………………………20

Appendix Tables:

Appendix Table S1………………………………………………………………………………21
Appendix Table S2………………………………………………………………………………22
Appendix Table S3………………………………………………………………………………23
Appendix Table S4………………………………………………………………………………24

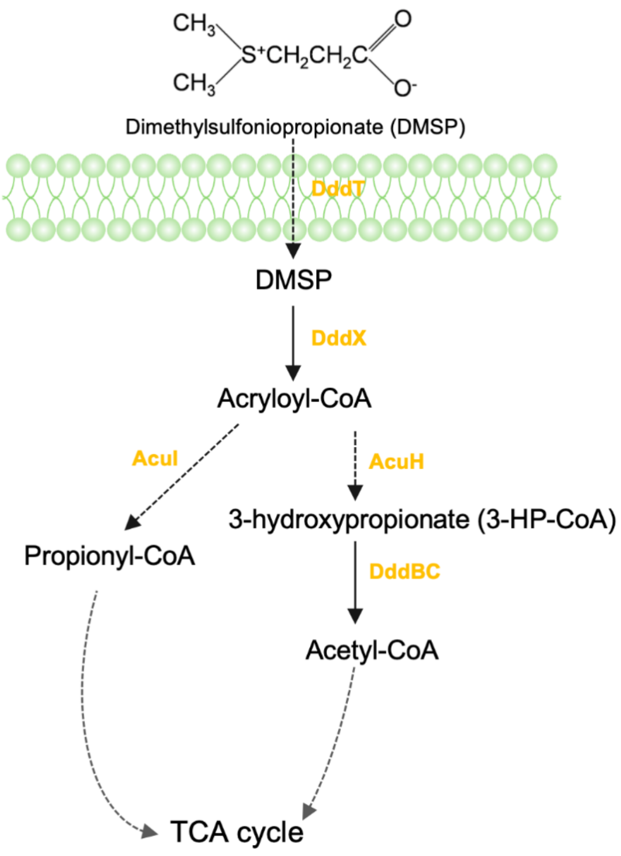

Appendix Figure S1: Metabolic pathway of DMSP in *Psychrobacter* sp. D2. Enzymes involved in DMSP metabolism are highlighted in orange.

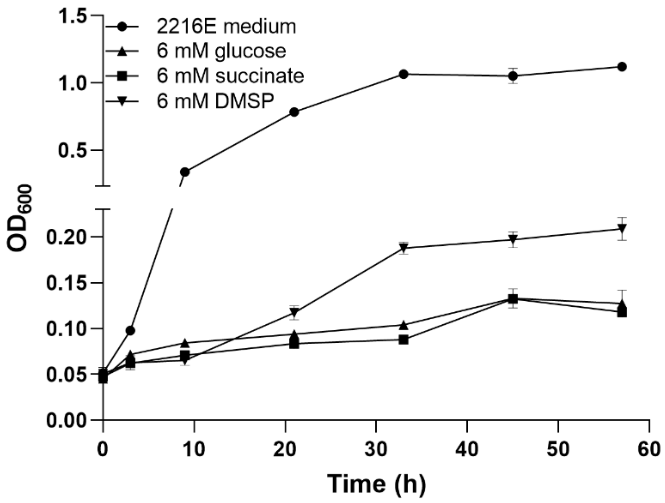

Appendix Figure S2: Growth of strain D2 on different carbon sources. Data are presented as the mean ± SD of triplicate determinations.

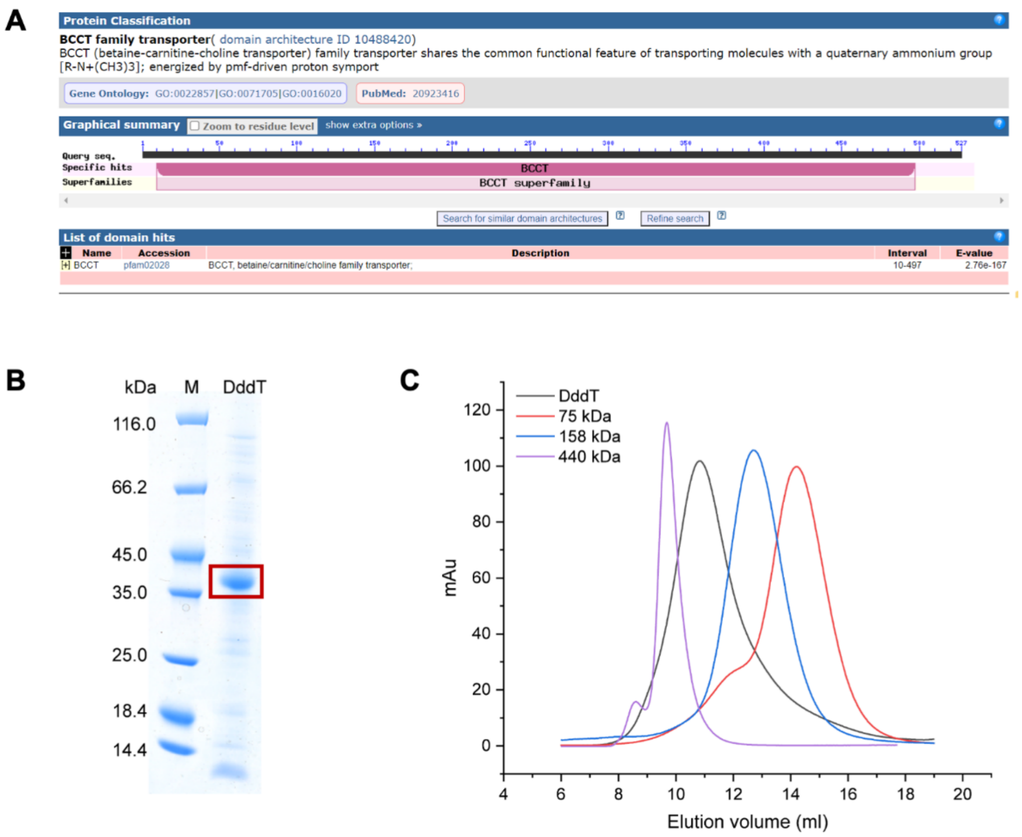

Appendix Figure S3: Protein classification, SDS-PAGE, and gel filtration analysis of DddT. A, Protein classification of DddT was performed using the Conserved Domain Search Service (CD Search) on the National Center for Biotechnology Information website (https://www.ncbi.nlm.nih.gov/Structure/cdd/wrpsb.cgi). **B**, SDS-PAGE analysis of the recombinant DddT. Protein standard size markers are indicated. **C**, Analysis of the association state of DddT in solution by gel filtration.

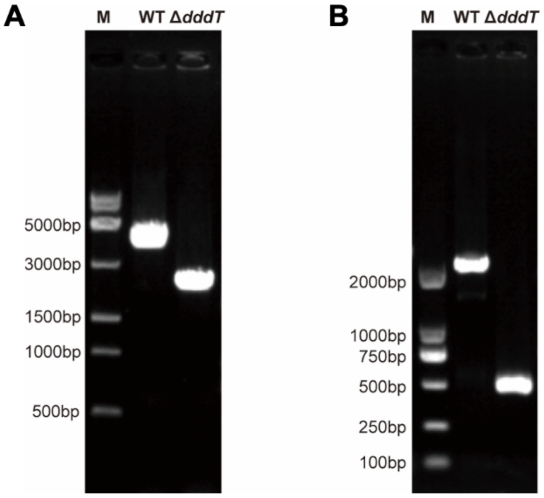

Appendix Figure S4: Construction of the *dddT* knockout mutant. **A**, PCR result using the *dddT*-LF/*dddT*-LR primers. The Δ*dddT* mutant generated a 2369 bp PCR product, while the product length was 3953 bp for the wild-type strain. **B**, PCR result using the *dddT*-SF/*dddT*-SR primers. The Δ*dddT* mutant generated a 667 bp PCR product, while the product length was 2251 bp for the wild-type strain.

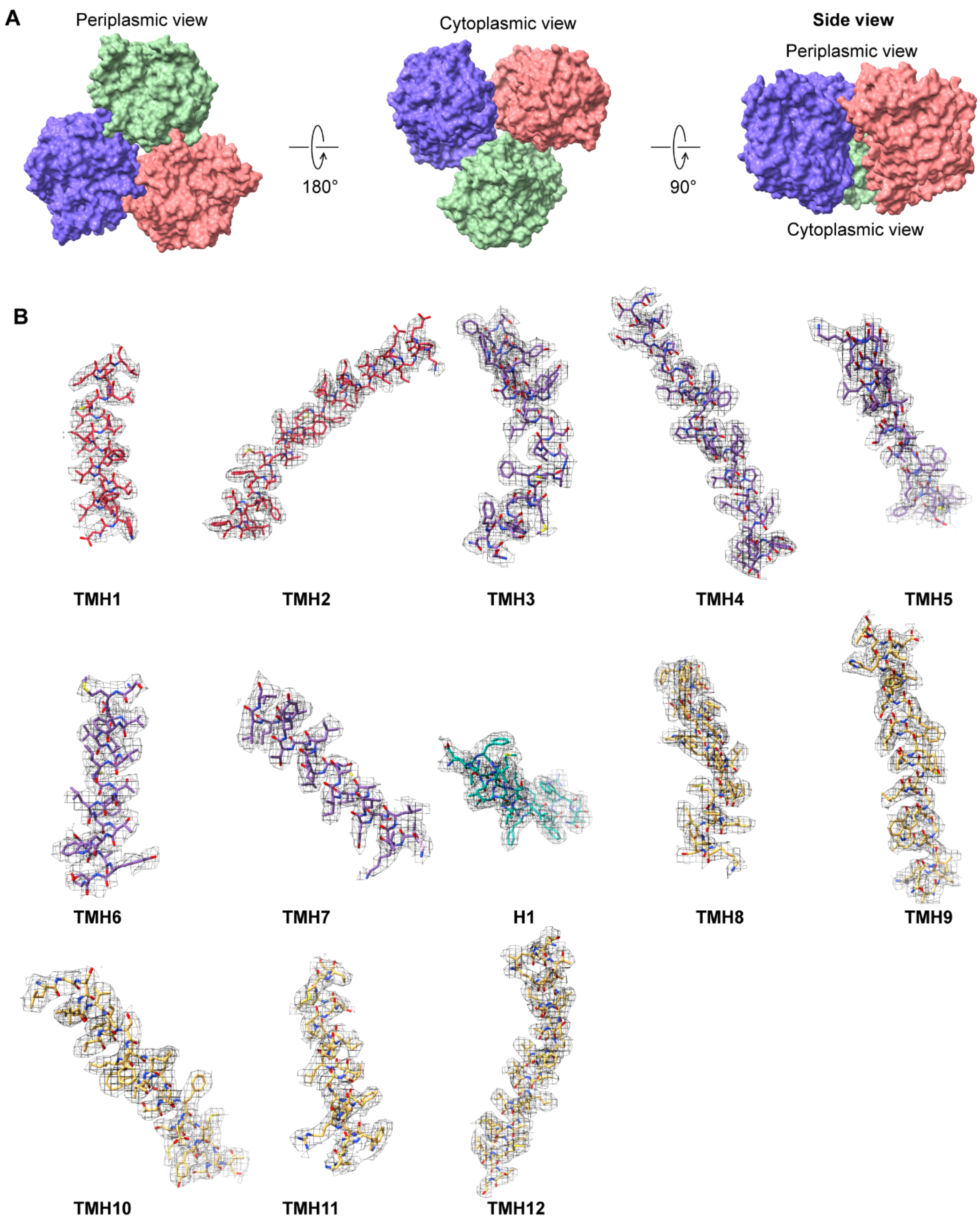

Appendix Figure S5: The trimer structure of DddT and the visualization of the α-helices density. A, A surface representation of a 2.80 Å cryo-EM map of the DddT trimer is shown. The three colors respectively represent three DddT monomers. Left, view from the periplasmic side; middle, view from the cytoplasmic side; right, side view in the membrane plane. **B**, Visualization of 12 transmembrane α-helices (TMH1-TMH12) and a bent α-helix (H1) are shown separately. The black mesh represents the cryo-EM density contoured at 3 RMSD.

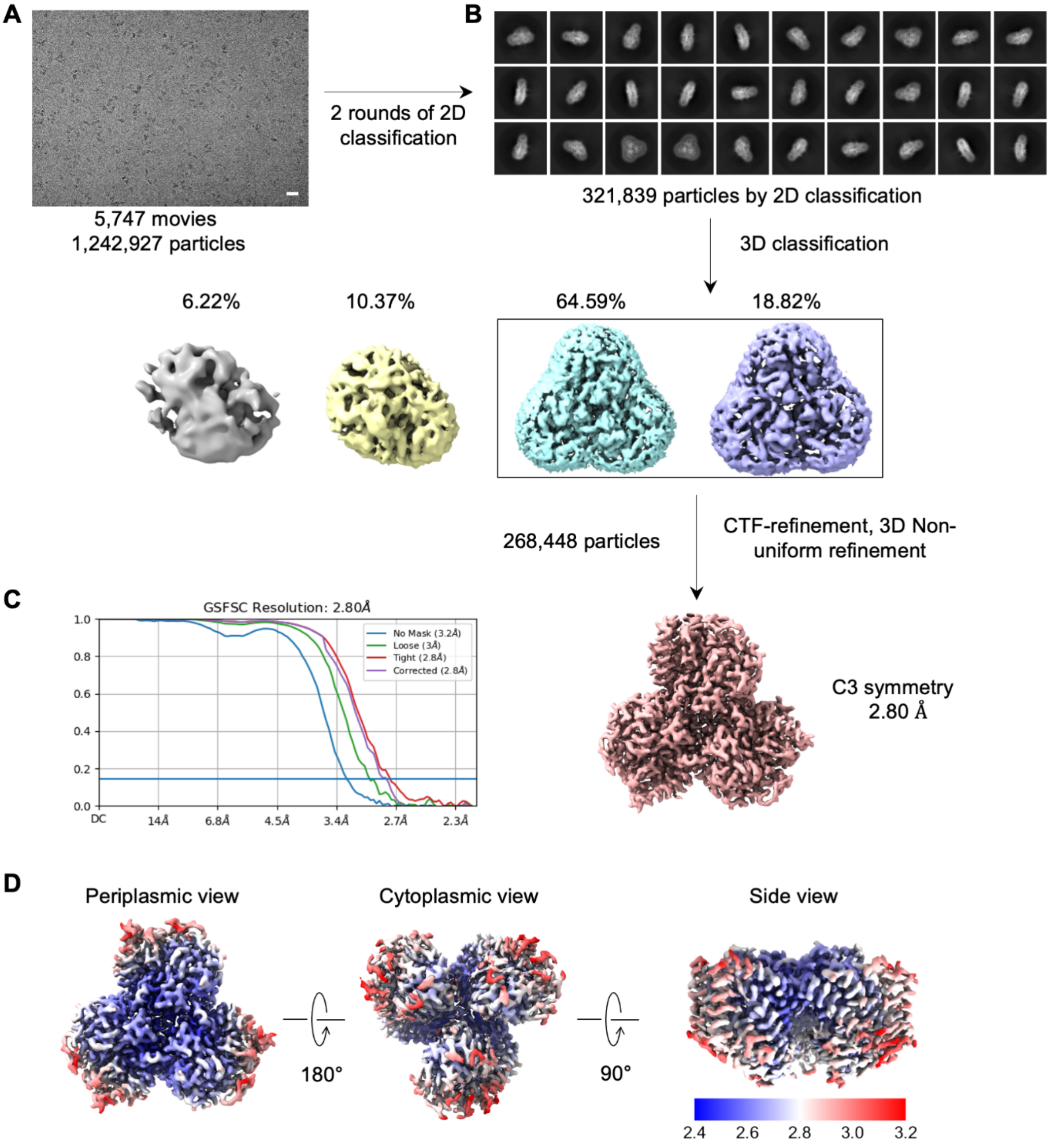

Appendix Figure S6: Cryo-EM data processing and determination of the structures of DddT in C_c state. **A**, Cryo-EM micrographs of C_c, 5,747 movies and 1,242,927 particles were collected. The scale bar represents 20 nm. **B**, After two rounds of 2D classification, the selected particles undergo 3D classification using C3 symmetry. CTF-refinement and 3D Non-uniform Refinement are performed to obtain a map with a resolution of 2.8 Å. **C**, Fourier shell correlation (FSC) of the refined map. **D**, Periplasmic view, cytoplasmic view, and side view of the electron density map of the DddT C_c state.

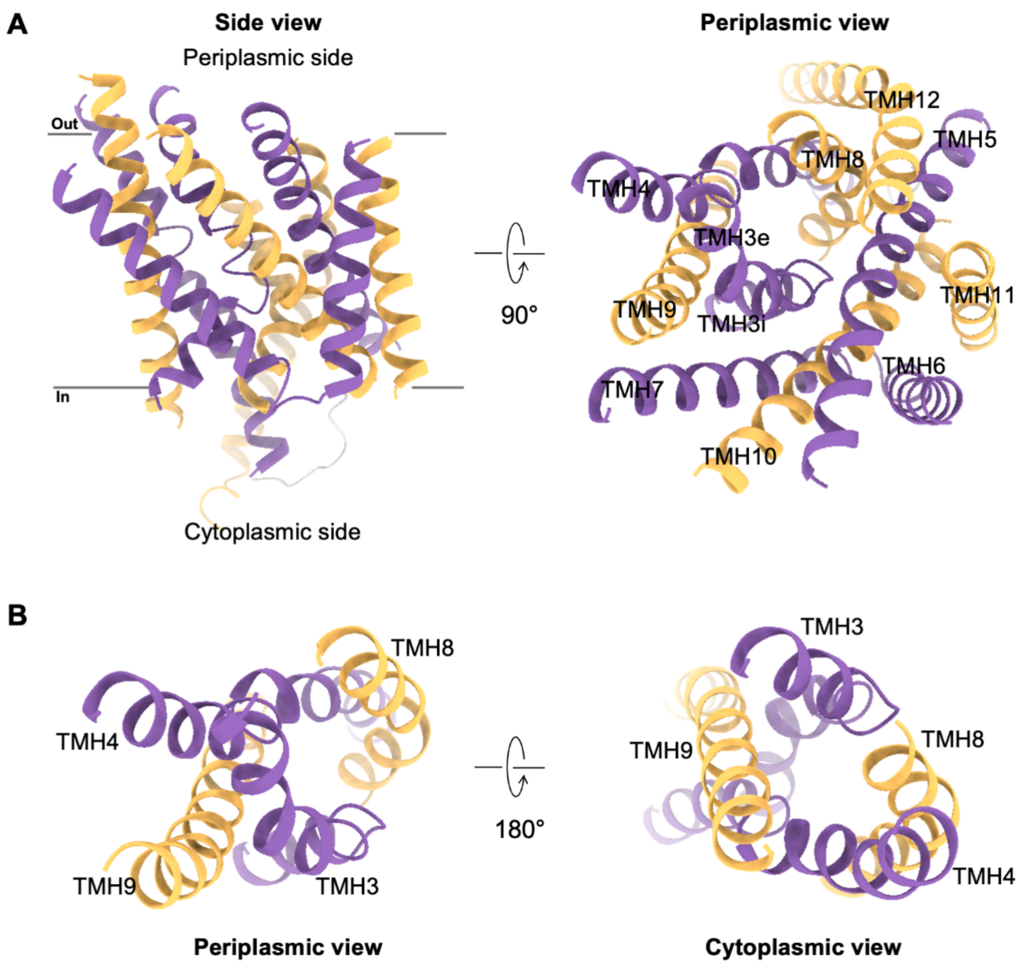

Appendix Figure S7: The FIRL-fold and V-shape helix pair of DddT structure. A, The top view and side view of two inverted structural repeats, repeat 1 (TM3-TM7) and repeat 2 (TM8-TM12), are colored purple and yellow. **B,** Periplasmic view and cytoplasmic view of V-shaped helix pair.

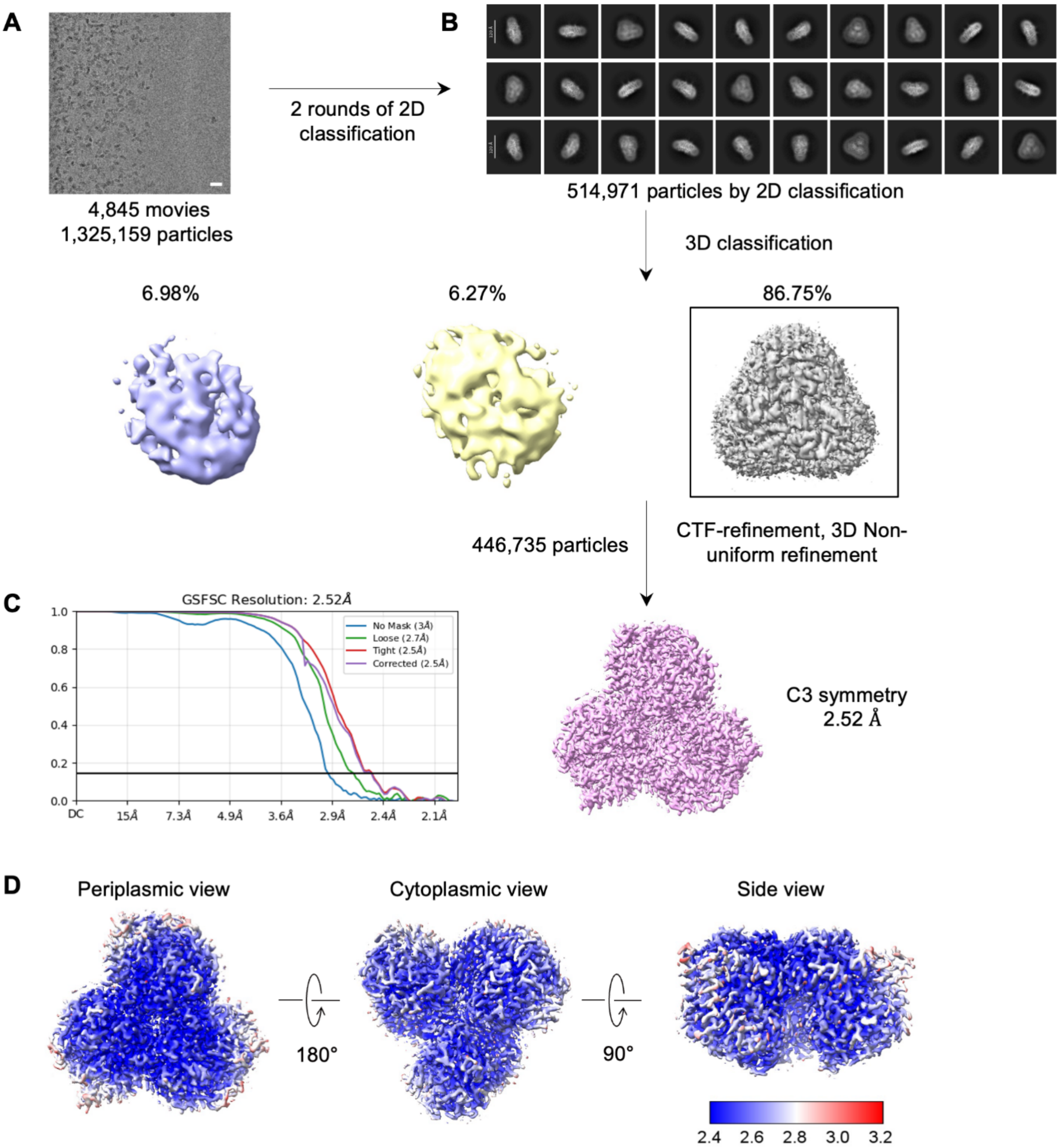

Appendix Figure S8: Cryo-EM data processing and determination of the structures of DddT in C_eS state. A, Cryo-EM micrographs of C_e, 4,845 movies and 1,325,159 particles were collected. The scale bar represents 20 nm. **B**, After two rounds of 2D classification, the selected particles undergo 3D classification using C3 symmetry. CTF-refinement and 3D Non-uniform Refinement are performed to obtain a map with a resolution of 2.52 Å. **C**, Fourier shell correlation (FSC) of the refined map. **D**, Periplasmic view, cytoplasmic view, and side view of the electron density map of the C_e state.

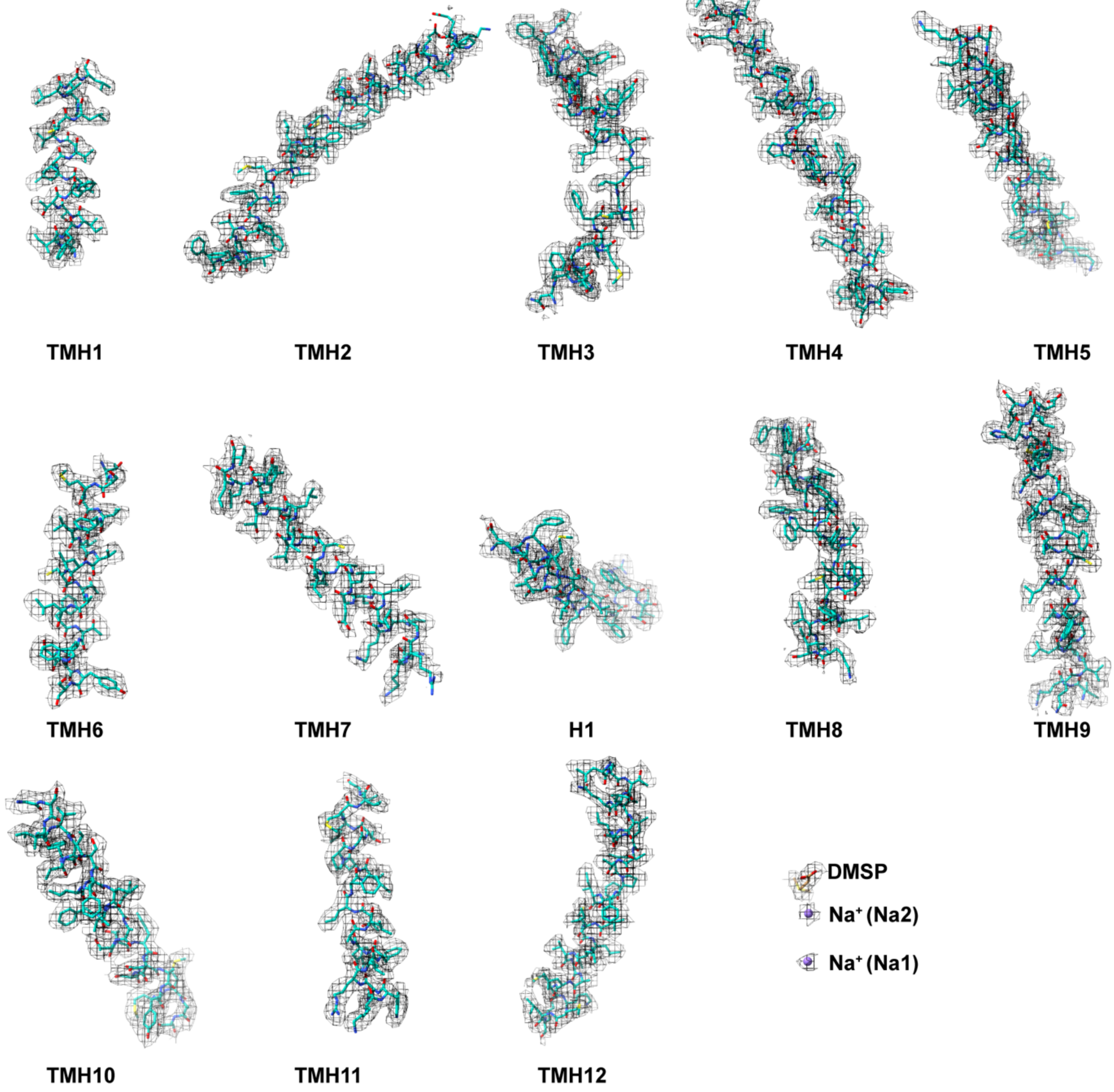

Appendix Figure S9: The visualization of the α-helices, the bound substrate and ions density of DddT in C_cS state. Visualization of the 12 transmembrane α-helices (TMH1–TMH12), the bent α-helix (H1), and the bound substrate and sodium ions are shown separately. The DMSP molecule and sodium ions are shown as brown stick and purple spheres, respectively. The black mesh represents the cryo-EM density contoured at 3 RMSD.

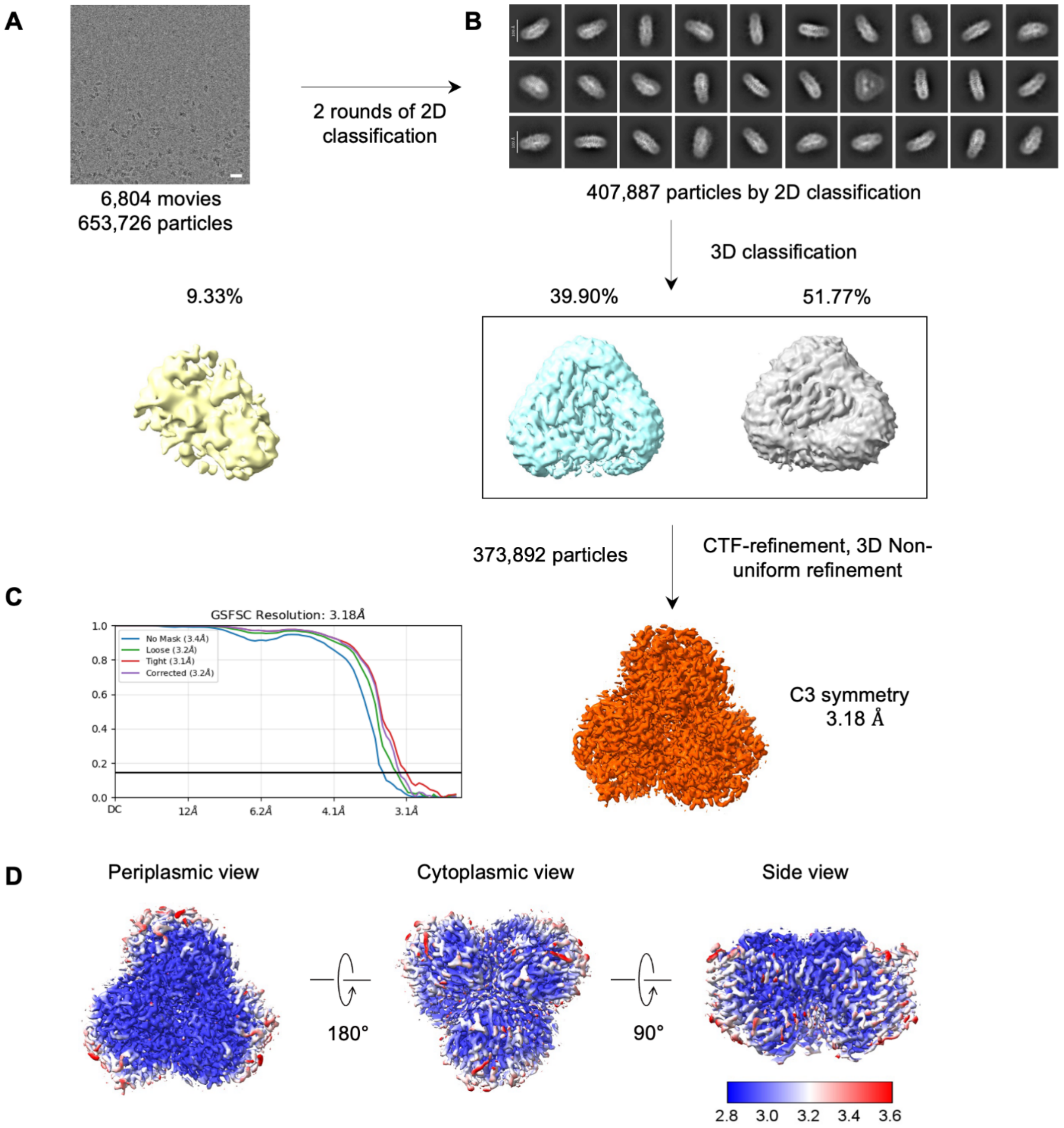

Appendix Figure S10: Cryo-EM data processing and determination of the structures of DddT in C_c-K^+ state. A, Cryo-EM micrographs of C_c-K^+, 6,804 movies and 653,726 particles were collected. The scale bar represents 20 nm. **B,** After two rounds of 2D classification, the selected particles undergo 3D classification using C3 symmetry. CTF-refinement and 3D Non-uniform Refinement are performed to obtain a map with a resolution of 3.18 Å. **C,** Fourier shell correlation (FSC) of the refined map. **D,** Periplasmic view, cytoplasmic view, and side view of the electron density map of the C_c-K^+ state.

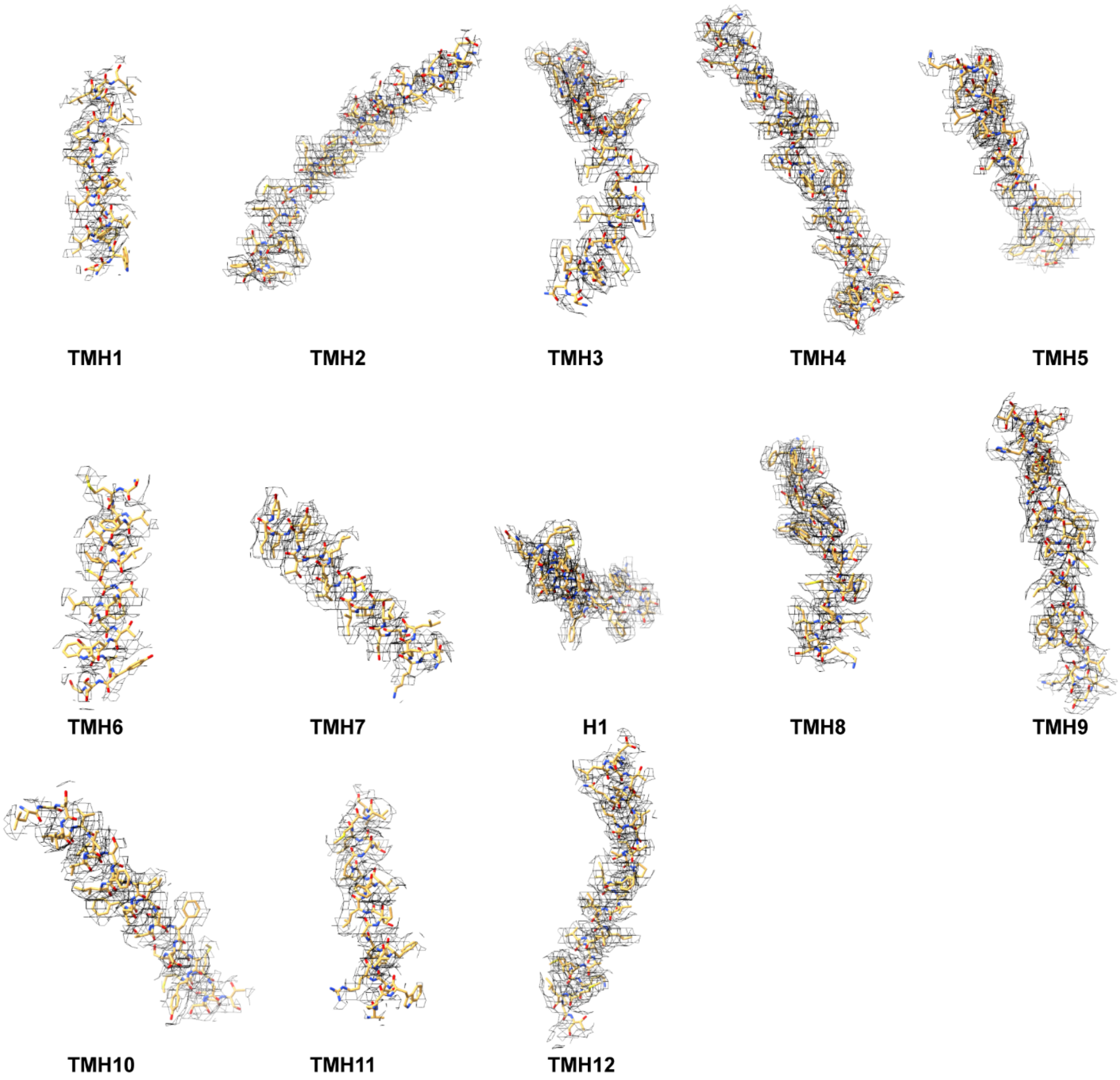

Appendix Figure S11: The visualization of the α-helices density of DddT in C_c-K^+ state. Visualization of the 12 transmembrane α-helices (TMH1–TMH12) and the bent α-helix (H1) are shown separately. The black mesh represents the cryo-EM density contoured at 3 RMSD.

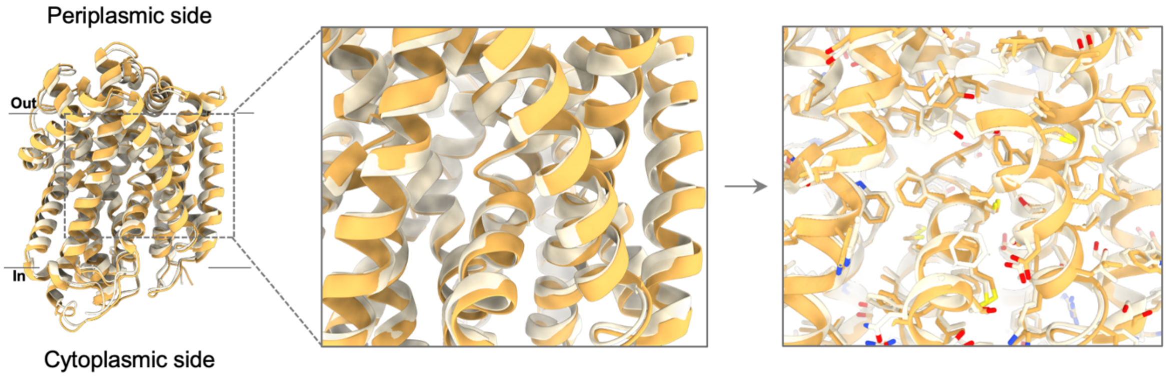

Appendix Figure S12: Comparison of the structures of the Cc state C_c-K^+ states of DddT. The C_c-K^+ state is colored yellow, while the Cc state is colored beige. The two structures were superimposed using the MatchMaker tool in UCSF ChimeraX. The alignment was performed based on backbone atoms. The backbone RMSD between the two structures is 1.03 Å over 497 pruned atom pairs (1.044 Å across all 500 atom pairs).

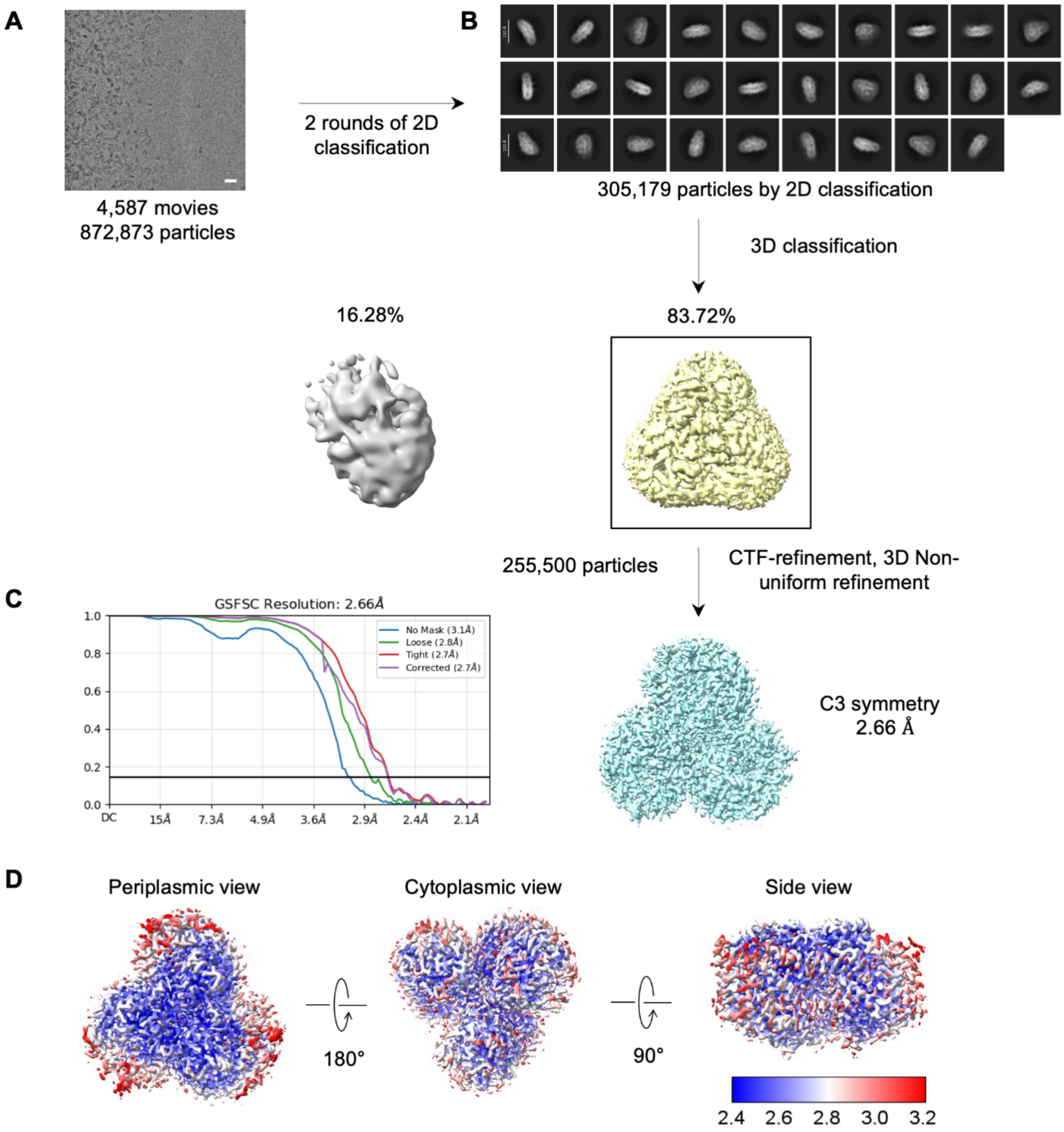

Appendix Figure S13: Cryo-EM data processing and determination of the structures of DddT in C_e state. **A**, Cryo-EM micrographs of C_e, 4,587 movies and 872,873 particles were collected. The scale bar represents 20 nm. **B**, After two rounds of 2D classification, the selected particles undergo 3D classification using C3 symmetry. CTF-refinement and 3D Non-uniform Refinement are performed to obtain a map with a resolution of 2.66 Å. **C**, Fourier shell correlation (FSC) of the refined map. **D**, Periplasmic view, cytoplasmic view, and side view of the electron density map of the C_e state.

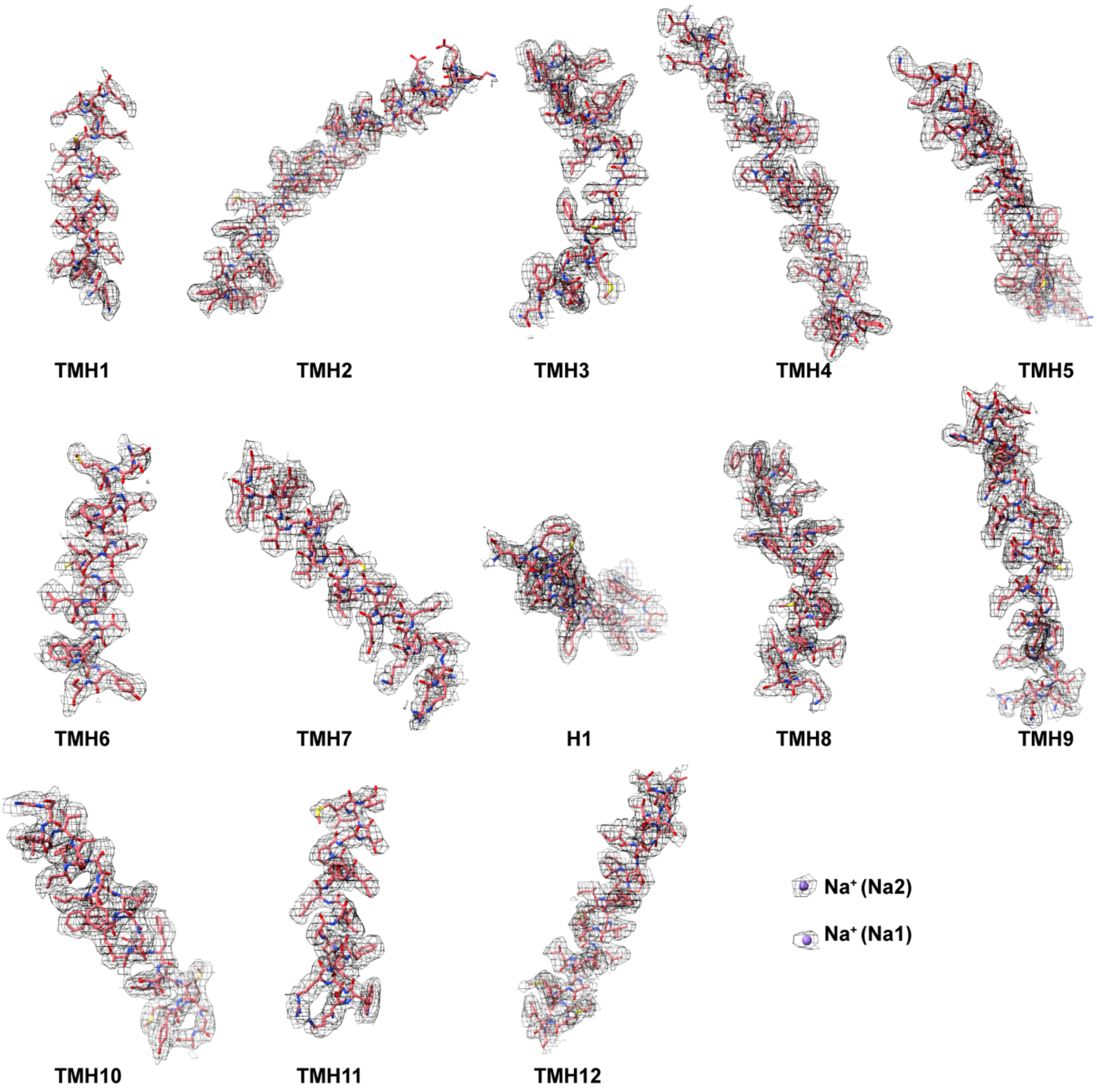

Appendix Figure S14: The visualization of the α-helices and the bound ion density of DddT in C_e state. Visualization of the 12 transmembrane α-helices (TMH1–TMH12), the bent α-helix (H1), and the bound sodium ion are shown separately. The sodium ion is shown as purple spheres. The black mesh represents the cryo-EM density contoured at 3 RMSD.

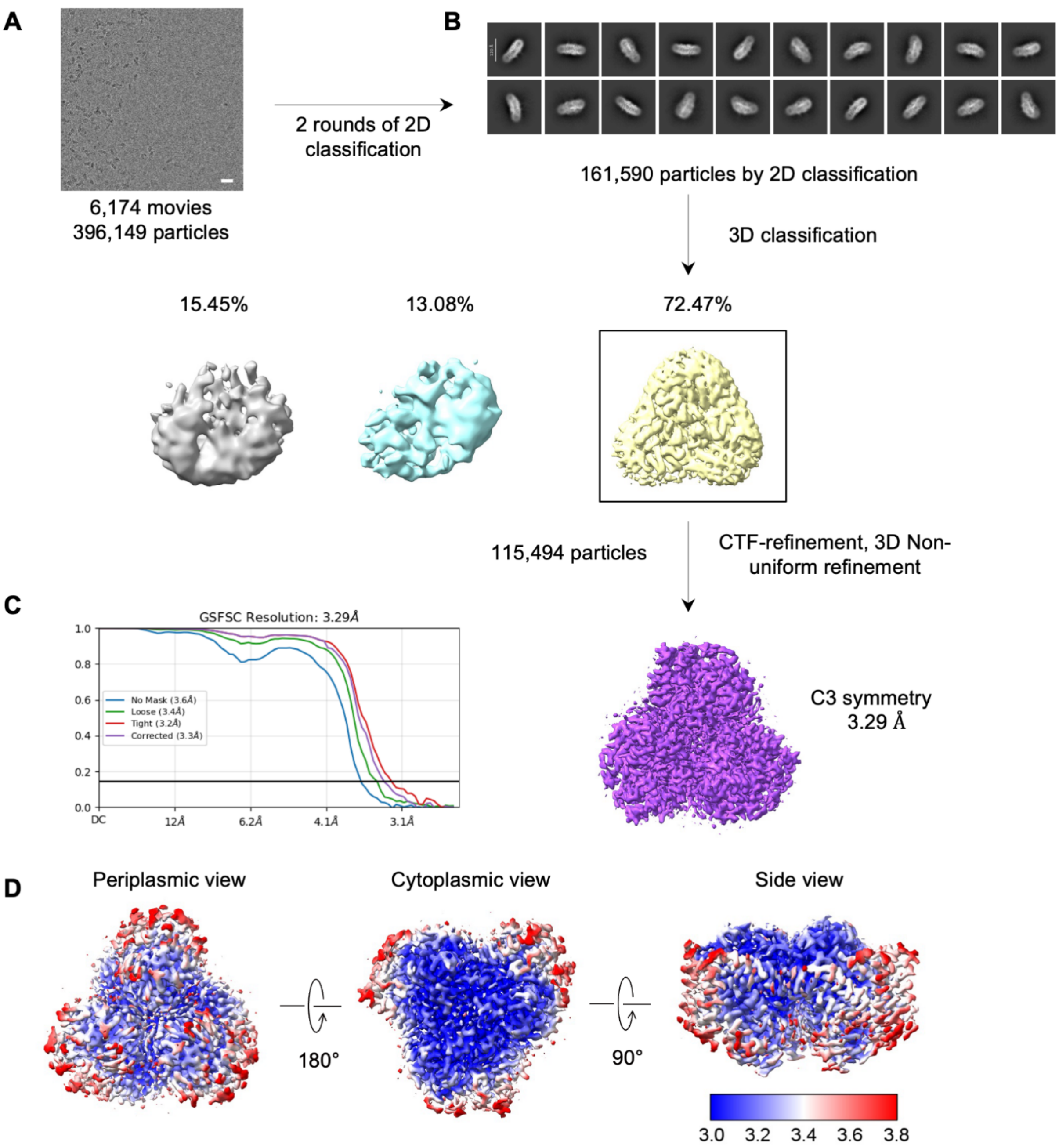

Appendix Figure S15: Cryo-EM data processing and determination of the structures of DddT in C_i state. **a**, Cryo-EM micrographs of C_i, 6,174 movies and 396,149 particles were collected. The scale bar represents 20 nm. **B**, After two rounds of 2D classification, the selected particles undergo 3D classification using C3 symmetry. CTF-refinement and 3D Non-uniform Refinement are performed to obtain a map with a resolution of 3.29 Å. **C**, Fourier shell correlation (FSC) of the refined map. **D**, Periplasmic view, cytoplasmic view, and side view of the electron density map of the C_i state.

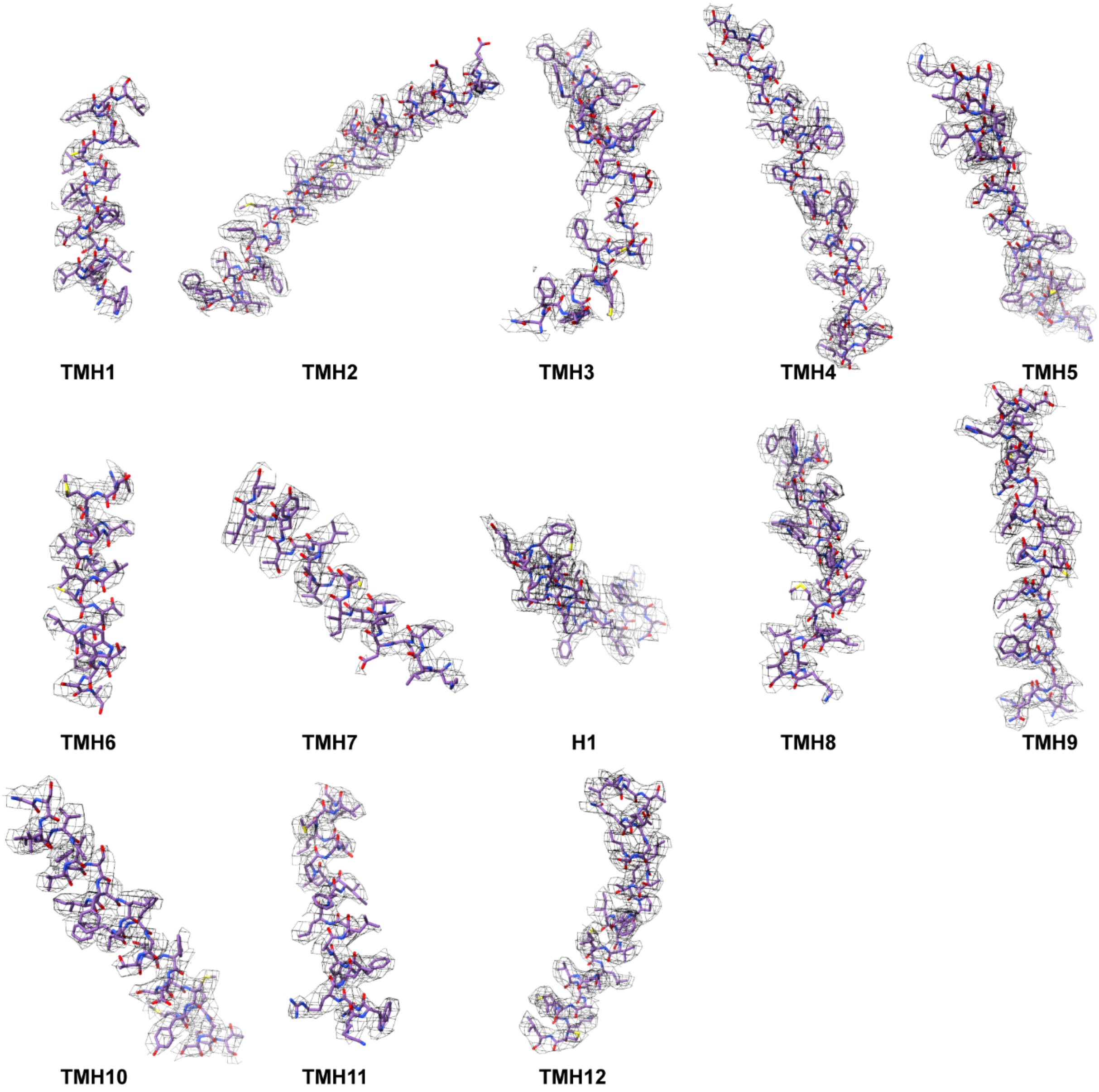

Appendix Figure S16: The visualization of the α-helices and the bound ion density of DddT in C_i state. Visualization of the 12 transmembrane α-helices (TMH1–TMH12), the bent α-helix (H1), and the bound sodium ion are shown separately. The sodium ion is shown as a purple sphere. The black mesh represents the cryo-EM density contoured at 3 RMSD.

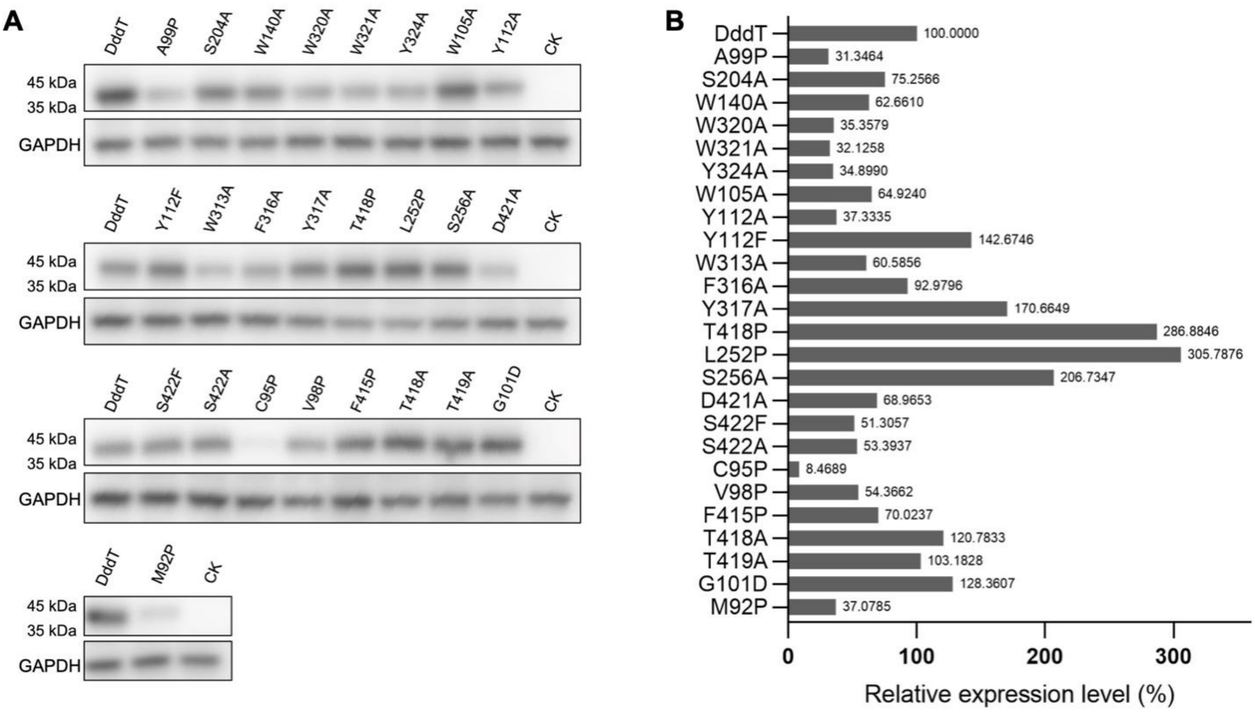

Appendix Figure S17: Protein expression levels of DddT and its mutants. A, Western blot analysis of DddT and its mutant variants performed in four independent experimental batches. All batches were performed under identical experimental conditions; however, unavoidable operational variability among batches (including incubation time, and other small procedural fluctuations) can lead to differences in signal amplitude. To address this, each batch included its own internal control, and the expression levels of mutant proteins were normalized accordingly. **B,** Quantification of normalized expression levels based on Western blot results, with the expression level of wild-type (WT) DddT set to 100%.

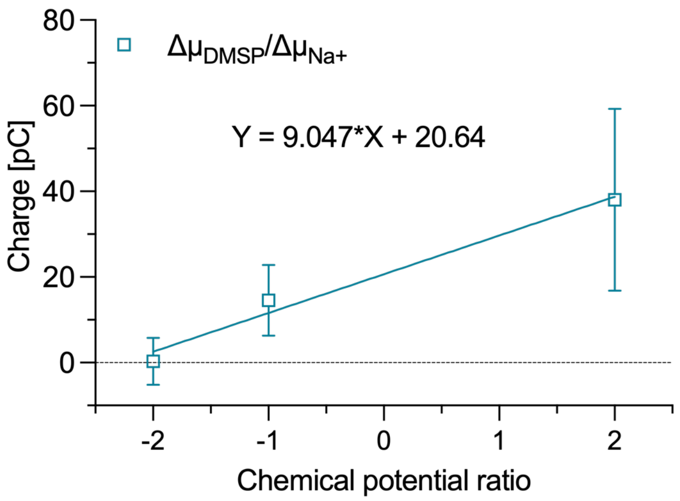

Appendix Figure S18: Determination of the Na^+/DMSP stoichiometry of DddT. Transport-associated charge (pC) was plotted as a function of the chemical potential ratio of DMSP to Na^+. The Na^+/DMSP stoichiometry was determined from the x-axis intercept at zero net charge. The estimated stoichiometric ratio of Na^+ to DMSP during transport is approximately 2. Data are presented as the mean ± SD of triplicate determinations.

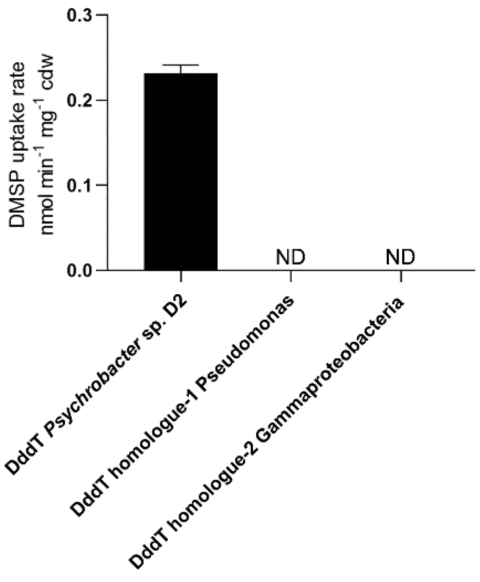

Appendix Figure S19: The DMSP uptake rates of DddT and randomly selected DddT homologues. The error bar represents the standard deviation of the data obtained from three biological replicates. Data are presented as mean ± SD (n = 3).

Appendix Table S1. Cryo-EM data collection, phasing, and refinement statistics.

**Data Collection and Processing**	C_c	C_cS	C_c-K^+	C_e	C_i
Microscope	Titan	Titan	Titan	Titan	Titan
Voltage (kV)	300kV	300kV	300kV	300kV	300kV
Magnification (nominal)	81,000	130,000	130,000	130,000	130,000
Electron Dose (e^- /$Å^2$)	64	60	60	60	60
Camera	Gatan K3	Thermo Scientific Falcon 4	Thermo Scientific Falcon 4	Thermo Scientific Falcon 4	Thermo Scientific Falcon 4
Defocus range (um)	−1.6~ −2.2	−0.8~−1.6	−0.8~−1.6	−0.8~−1.6	−0.8~−1.6
Pixel size (Å)	0.53	0.97	0.97	0.97	0.97
Movies collected	5,747	4,845	6,804	4,587	6,174
Symmetry	C3	C3	C3	C3	C3
Final particle images (no.)	268,448	446,735	373,892	255,500	115,494
Map resolution (Å)	2.8	2.52	3.18	2.66	3.29
Sharpening B-factor ($Å^2$)	-140.9	-95.1	-126.2	-95.3	-103.8
Software used to process data	cryoSPARC v3.3.2	cryoSPARC v4.5.1	cryoSPARC v4.5.1	cryoSPARC v4.5.1	cryoSPARC v4.5.1
**Refinement statistics**					
Number of protein atoms (non-H)	3864	3893	3863	3870	3858
R.m.s. deviations					
Bonds (Å)	0.006	0.007	0.006	0.004	0.007
Bond angles (º)	1.032	1.180	1.080	1.017	1.181
Validation					
MolProbity score	1.72	0.94	1.74	1.07	1.49
Clash score	7.29	1.78	3.97	2.30	4.36
Poor rotamers (%)	0.00	0.49	3.41	1.22	1.46
Ramachandran plot					
Favored (%)	95.38	98.60	97.19	98.19	97.18
Allowed (%)	4.62	1.40	2.81	1.81	2.82
Disallowed (%)	0.00	0.00	0.00	0.00	0.00
EMDB access code	EMD-67623	EMD-67626	EMD-67627	EMD-67625	EMD-67628
PDB access code	21FF	21FI	21FJ	21FH	21FK

Appendix Table S2. Kinetic parameters for apparent sodium affinity measured from betaine uptake of WT DddT and mutants.

	Site	K_m (mM)	V_{max} (nmol min^{-1} mg^{-1} cdw)
DddT		24.52 ± 2.45	0.33 ± 0.01
M92P/L252P/D421A	Na1	—	—
S256A	Na1	63.54 ± 7.17	0.10 ± 0.005
V98P	Na2	18.71 ± 1.03	0.18 ± 0.003
T418A	Na2	53.91 ± 10.1	0.39 ± 0.03
T419A	Na2	56.64 ± 10.44	0.10 ± 0.008

Appendix Table S3. Primers used in this study.

Primers	Sequence (5'-3')	Purpose
dddT-Up-F	GTAAAACGACGGCCAGTGCCAAGCTTAAATATAAAGAGTTTTTAGA	Upstream homologous fragment of the *dddT* gene
dddT-Up-R	AAGTCCTTAAATCATCTGATTTTAATTACTCCTTCCTTAG	Upstream homologous fragment of the *dddT* gene
dddT-Down-F	CTAAGGAAGGAGTAATTAAAATCAGATGATTTAAGGACTT	Downstream homologous fragment of the *dddT* gene
dddT-Down-R	GTCATAAGATTAGTCACTGGGGATCCTTCTCATTTGAAAAATGAAA	Downstream homologous fragment of the *dddT* gene
dddT-LF	GGATGCTGATATTGGAATCATAACC	Confirmation of Δ*dddT*
dddT-LR	AGTGGTTGATGCGAGATGATGACA	Confirmation of Δ*dddT*
dddT-SF	CCAGCATTAGACTTAGAAGCCCTCA	Confirmation of Δ*dddT*
dddT-SR	CTGCTTGATCCAATGCTTCCCATAT	Confirmation of Δ*dddT*
dddT-pBBR-F	CGACGGTATCGATAAGCTTGAAGCGTTTATACATTATATA	Complementation of Δ*dddT*
dddT-pBBR-R	CTCTAGAACTAGTGGATCCCCCTAAGCAGCTGGTTTTTGC	Complementation of Δ*dddT*
*ddd*T-F	AAGAAGGAGATATACATATGTTGACTGGTCAGATAATTGAG	Amplification of the *dddT* gene
dddT-R	TGGTGGTGGTGGTGCTCGAGCACCAGCTCACTACATTTTTG	Amplification of the *dddT* gene
22b-Bba-*dddT*-F	ACTTTAAGAAGGAGATATACATTTGACGGCTAGCTCAGTCCTAGGTATAGTGCTAGCATGAAAGAGAATAAAATTGAC	Construct of the constitutive plasmid
22b-Bba-*dddT*-R	CTCAGTGGTGGTGGTGGTGGTGCTCGAGCTAAGCAGCTGGTTTTTGCCG	Construct of the constitutive plasmid

Appendix Table S4. Composition of the basal medium (lacking the carbon source).

Solution	Components
1	0.05% (w/v) NH_4Cl, 3% (w/v) NaCl, 0.3% (w/v) $MgCl_2 \cdot 6H_2O$, 0.2% (w/v) K_2SO_4, 0.02% (w/v) K_2HPO_4, 0.001% (w/v) $CaCl_2$, 0.0006% (w/v) $FeCl_3 \cdot 6H_2O$, 0.0005% (w/v) $Na_2MoO_4 \cdot 7H_2O$, 0.0004% (w/v) $CuCl_2 \cdot 2H_2O$, 0.6% (w/v) Tris.
2	0.001% (w/v) thiamine·HCl, 0.002% (w/v) nicotinic acid, 0.002% (w/v) pyridoxine·HCl, 0.002% (w/v) riboflavin, 0.0001% (w/v) biotin, 0.0001% (w/v) cyanocobalamin, 0.001% (w/v) *p*-aminobenzoic acid, 0.002% (w/v) calcium pantothenate.

Solution 1 was autoclaved at 121°C for 20 min. Solution 2 was filter-sterilized before it was combined with Solution 1.
