# Supplementary material for: Structural insights into bacterial dimethylsulfoniopropionate import by BCCT-family transporters
Source: EMBO J. 2026 May 8;45(12):4299–320. doi: 10.1038/s44318-026-00798-w (PMC13270143; doi:10.1038/s44318-026-00798-w)
Supplement: Supplementary file 10 — Expanded View Figures [file 44318_2026_798_MOESM10_ESM.pdf]

Expanded View Figures

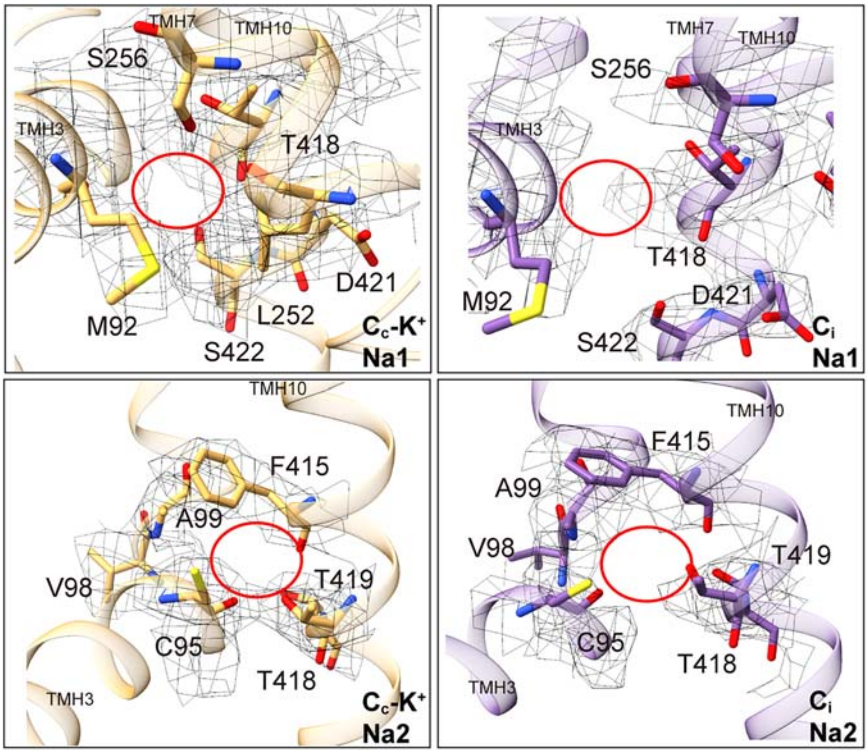

Figure EV1. Loss of cryo-EM densities at the putative Na1 and Na2 sites in the C_c-K^+ and C_i structures determined in Na^+-free buffer.

The gray mesh shows the local cryo-EM density of the residues involved in sodium binding, contoured at 4 RMSD. The densities corresponding to Na1 and Na2, which are clearly observed in the C_e and C_cS states, are absent under Na^+-free conditions.

 © The Author(s)

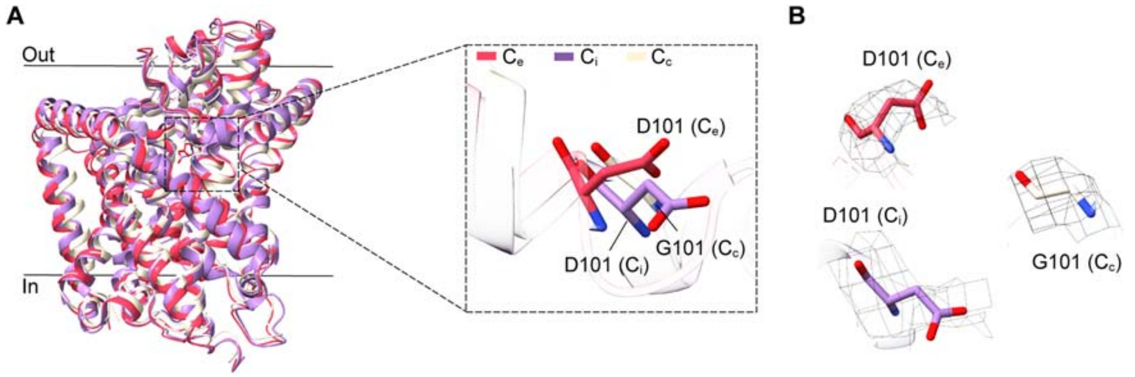

Figure EV2. Structural comparison of residue 101 in DddT among the C_c, C_e, and C_i states.

(**A**) Structural differences of residue 101 between the C_c, C_e, and C_i states. The C_c, C_e, and C_i states are colored in beige, red, and purple, respectively. (**B**) Cryo-EM densities surrounding Asp101 (C_e), Asp101 (C_i), and Gly101 (C_c), contoured at 6 RMSD, 4 RMSD, and 8 RMSD, respectively.

© The Author(s)

		Na1 binding site DddT		Na2 binding site DddT		Na1 binding site BetP
		252 256	418 421 422	95 98 99	415 418 419	197 201
★	DddT *Psychrobacter* sp. D2	GLKLLSD	ATTFDSA	FCAGVAS	IFLATTF	GILGAAG
★	DddT *Halomonas* sp. HTNK1	GIRILSD	ATTLDSS	FAGGIGI	IFLATTL	GIVGGVG
★	BccT5 *V. vulnificus* YJ016	GIKVLSN	ATTFDSI	FCGGIGA	IFTATTF	GLLGGAA
	DddT *Marinomonas* sp. MWYL1	GIKILSE	VTSADSA	FSAGIGI	TYFVTSA	GTVFGIA
	DddT *Pseudomonas* sp. J465	GIRMLSE	VTSADSA	FGAGIGI	TYFVTSA	GTVFGIA
	DddT *Psychrobacter* sp. J466	GVKWLSN	VTTLDSA	FGAGIGV	TYLVTTL	ATILGVA
★	BccT2 *V. parahaemolyticus* RIMD2210633	GVKRLSE	ITSSDSG	FAAGMGI	VFFITSS	ATVFGLA
★	BccT3 *V. cholerae* N16961	GVKVISN	ITSSDSG	FAAGMGI	VFFITSS	ATLFGLA
	WP_142056479.1 *Pseudonocardia kunmingensis*	GIQWLSN	VSGADAA	FSAGMGI	IFFVSGA	ATLFGSA
	WP_285937307.1 *Actinomadura xylanilytica*	GIQWLSN	VSGADAA	FSAGMGI	IFFVSGA	ATLFGSA
	WP_092224259.1 *Desulforhopalus singaporensis*	GIRILSL	VTTSDSA	FSAGFGL	VFGVTTS	STLGGNG
	WP_066084478.1 *Hydrogenophaga crassostreae*	GIKILSD	ATTLDSA	FCGGIGI	IFLATTL	GIVGGVA
	WP_088916717.1 *Granulosicoccus antarcticus*	GIRILSD	ATTLDSA	FCGGIGI	IFLATTL	GVIGGVG
	WP_095133227.1 *Anaeromicrobium sediminis*	GVKLLSE	VTSSDSG	FSAGIGI	LFFVTSS	SVLFGLA
	WP_108133289.1 *Halomonas*	GIKILSD	ATTLDSS	FAGGIGI	IFLATTL	GIVGGVG
	WP_110674477.1 *Salinicola*	GIRILSD	ATTLDSS	FAGGIGI	IFLATTL	GIVGGVG
	WP_118711689.1 *Enterocloster aldenensis*	GKKTISN	ASSLDSA	ALAALAS	GFVASSL	CIIGAAG
	WP_191237274.1 *Cobetia*	GIKILSD	ATTLDSS	FTCGMGI	IFLATTL	GIVGGVG
	WP_191237276.1 *Cobetia pacifica*	GIKILSD	ATTLDSS	FAGGIGI	IFLATTL	GIVGGVG
	WP_169641146.1 *Roseobacter ponti*	GIKILSD	ATTLDSA	FCGGIGI	IFLATTL	GIVGGVG
	WP_186674364.1 *Pseudomonas oryzicola*	GIKILSD	ATTLDSA	FCGGIGI	VFLATTL	GIVGGVG
	WP_215375185.1 *Pseudomonas boanensis*	GIKILSD	ATTLDSA	FCGGIGI	VFLATTL	GIVGGVG
	WP_225346819.1 *Cobetia amphilecti*	GIKILSD	ATTLDSS	FAGGIGI	IFLATTL	GIVGGVG
	WP_279833044.1 *Cobetia litoralis*	GIKILSD	ATTLDSS	FAGGIGI	IFLATTL	GIVGGVG
	WP_074750954.1 *Pseudomonas abietaniphila*	GIKLLSD	ATTLDSA	FCGGIGI	VFLATTL	GIVGGVG
	WP_085879181.1 *Roseisalinus antarcticus*	GIKILSD	ATTLDSA	FCGGIGI	IFLATTL	GVVGGVG
	WP_013794832.1 *Marinomonas posidonica*	GIKILSE	VTSADSA	FSAGIGI	TYFVTSA	GTVFGIA
	WP_016855432.1 *Halomonas smyrnensis*	GIKWLSN	VTSADSA	FATGMGI	LFFVTSA	STVIGLS
	WP_002538432.1 *Grimontia indica*	GIRILSE	VTSADSA	FGAGIGI	TYFVTSA	GTVFGIA
	WP_016417475.1 *Halomonas anticariensis*	GIRILSE	VTSADSA	FGAGIGI	TYFVTSA	GTVFGIA
	WP_022951303.1 *Leucothrix mucor*	GIKWLSN	VTTADSA	FGAGIGI	TYLVTTA	ATVLGVA
	WP_027258061.1 *Leisingera aquimarina*	GVKYLSN	VTSADSG	FGAGLGV	TFLVTSA	ATILGVS
	WP_024089211.1 *Leisingera methylohalidivorans*	GVKYLSN	VTSADSG	FGAGLGV	TFLVTSA	ATILGVS
	WP_028024020.1 *Enterovibrio calviensis*	GIRILSE	VTSADSA	FGAGIGI	TYFVTSA	GTVFGIA
	WP_055463442.1 *Marinomonas fungiae*	GINMLSQ	VTAADSG	FGCGTGA	VFFVTAA	GTIFGIA
	WP_067385204.1 *Marinobacterium aestuarii*	GIRILSE	VTSADSA	FGAGIGI	TYFVTSA	GTVFGIA
	WP_071472804.1 *Oceanisphaera psychrotolerans*	GIKVISE	VTSSDSS	FSAGIGI	SWFVTSS	GCVFGVA
	WP_094199895.1 *Oceanimonas*	GVKRLSE	VTSSDSG	FAAGMGI	VFFVTSS	ATLFGLA
	WP_108385793.1 *Yoonia sediminilitoris*	GIKWLSN	VTTADSA	FGAGIGI	TYLVTTA	ATILGVA
	WP_116473943.1 *Zobellella maritima*	GIKWLSN	VTSADSA	FATGMGI	LFFVTSA	STVIGLS
	WP_109822491.1 *Leucothrix arctica*	GIKWLSN	VTTADSA	FGAGIGV	TYLVTTA	ATILGVA
	WP_097805652.1 *Pelagimonas varians*	GIKWLSN	VTTADSA	FGAGIGI	TYLVTTA	ATILGVA
	WP_066598641.1 *Celeribacter halophilus*	GIKWLSN	VTTADSA	FGAGIGI	TYLVTTA	ATILGVA
	WP_113872885.1 *Marinomonas aquiplantarum*	GIKILSE	VTSADSA	FSAGIGI	TYFVTSA	GTVFGIA
	WP_115467766.1 *Marinomonas piezotolerans*	GINMLSQ	VTAADSG	FGCGTGA	VFFVTAA	GTIFGIA
	WP_113918092.1 *Marinomonas rhizomae*	GIKILSE	VTSADSA	FSAGIGI	TYFVTSA	GTVFGIA
	WP_129495376.1 *Enterovibrio baiacu*	GIRILSE	VTSADSA	FGAGIGI	TYFVTSA	GTVFGIA
	WP_136464757.1 *Aliishimia ponticola*	GIKWLSN	VTTADSA	FGAGIGI	TYLVTTA	ATILGVA
	WP_136665970.1 *Pseudomonas leptonychotis*	GIRMLSE	VTSADSA	FGAGIGI	TYFVTSA	GTVFGIA
	WP_146786251.1 *Colwellia demingiae*	GIKILSE	VTSADSA	FGAGIGI	TYFVTSA	GTVFGIA
	WP_153718230.1 *Spiribacter salilacus*	GLKWLSL	ITSSDSG	FSAGMGI	TYFITSS	GTLFGLA
	WP_159963523.1 *Profundibacterium mesophilum*	GVKVLSE	VTSSDSG	FAAGMGI	VFFVTSS	ATLFGLA
	WP_183475125.1 *Limimaricola variabilis*	GIKWLSN	VTTADSA	FGAGIGV	TFLVTTA	ATVLGVS
	WP_189641528.1 *Amylibacter ulvae*	GIKWLSN	VTTADSA	FGAGIGI	TYLVTTA	ATILGVA
	WP_191161141.1 *Bacillus sp. IB182487*	GIKYLSN	VTSADSA	FSAGMGI	TFFVTSA	ATIFGVA
	WP_191600050.1 *Marinomonas algicola*	GINMLSQ	VTAADSG	FGCGTGA	VFFVTAA	GTIFGIA
	WP_213639020.1 *Pseudomonas lalucatii*	GVKWLSN	VTSADSA	FGAGIGV	TFLVTSA	ATLLGIA
	WP_213641632.1 *Pseudomonas lalucatii*	GIKIISE	VTSSDSG	FSAGIGI	SWFVTSS	GCVFGIA
	WP_218451483.1 Roseobacteraceae	GIKWLSN	VTTADSA	FGAGIGI	TYLVTTA	ATILGVA
	WP_222223896.1 *Marinobacterium arenosum*	GIRILSE	VTSADSA	FGAGIGI	TYFVTSA	GTVFGIA
	WP_253963426.1 *Aurantimonas marianensis*	GVKRLSE	VTSSDSG	FAAGMGI	VFFVTSS	ATLFGLA
	WP_264210529.1 *Leisingera sp. BMJM1*	GVKYLSN	VTSADSG	FGAGLGV	TFLVTSA	ATILGVS
	WP_282351393.1 *Pontibacterium granulatum*	GIRILSE	VTSADSA	FGAGIGI	TYFVTSA	GTVFGIA
	WP_067033200.1 *Marinomonas gallaica*	GINMLSQ	VTAADSG	FGCGTGA	VFFVTAA	GTIFGIA
	WP_090240933.1 *Pseudomonas guineae*	GIRMLSE	VTSADSA	FGAGIGI	TYFVTSA	GTVFGIA
	WP_092082295.1 *Poseidonocella sedimentorum*	GIKWLSN	VTTADSA	FGAGIGI	TYLVTTA	ATILGVA
	WP_093992248.1 *Flavimaricola marinus*	GIKWLSN	VTTADSA	FGAGIGI	TYLVTTA	ATILGVA
	WP_163390697.1 *Enterovibrio norvegicus*	GIRILSE	VTSADSA	FGAGIGI	TYFVTSA	GTVFGIA
	WP_189582206.1 *Psychrobacter glaciei*	GVKWLSN	VTTLDSA	FGAGIGV	TYLVTTL	ATILGVA

 © The Author(s)

Figure EV3. Multi-sequence alignment of the experimentally ratified DddT proteins and 61 identified DddT homologues whose genes are linked to the DMSP lyase genes DddD/DddX.

Key residues around the sodium-binding site are marked out. Based on the hydrophilicity and hydrophobicity of key residues at the Na1-binding site of BetP, sequences are divided into three categories. In the gray box, there is one hydrophilic residue and one hydrophobic residue. In the blue box, both residues are hydrophilic, and in the pink box, both residues are hydrophobic. The functional DddTs are marked with ★. Hydrophilic residues are colored green, and hydrophobic residues are colored orange. Source data are available online for this figure.

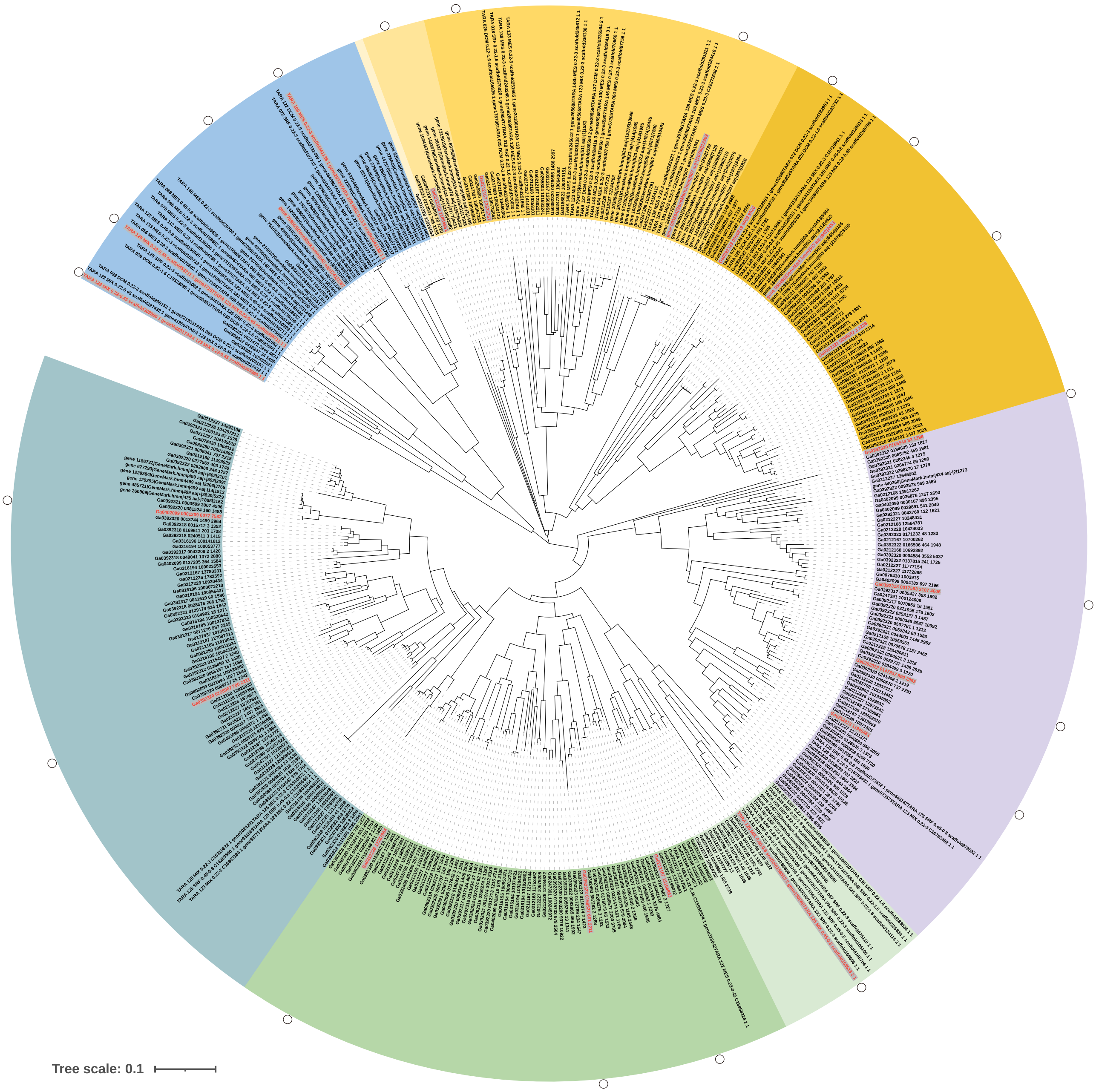

Figure EV4. Phylogenetic analysis of selected 411 DddT homologues from metagenome data.

Branches with different colors represent distinct clades in the phylogenetic tree. The sequences highlighted by shading and shown in red within the circle correspond to 20 randomly selected sequences from different branches of the phylogenetic tree, which were used for the multiple sequence alignment in Fig. 6C. Source data are available online for this figure.

 © The Author(s)
