# Supplementary material for: Structural insights into bacterial dimethylsulfoniopropionate import by BCCT-family transporters
Source: EMBO J. 2026 May 8;45(12):4299–320. doi: 10.1038/s44318-026-00798-w (PMC13270143; doi:10.1038/s44318-026-00798-w)
Supplement: Supplementary file 1 — Peer Review File [file 44318_2026_798_MOESM1_ESM.pdf]

THE EMBO JOURNAL

Structural insights into bacterial dimethylsulfoniopropionate import by BCCT-family transporters

Yu-Zhong Zhang, Wen-Jing Zhu, Kang Li, Hai-Tao Ding, Motoyuki Hattori, Shuaimeng Liu, Chang Ge, Qi-Long Qin, Zhao-Jie Teng, Ning-Hua Liu, Hai-Yan Cao, Chun-Yang Li, Xiu-Lan Chen, Qingtao Shen, Jonathan Todd, Lu-Ning Liu, and Peng Wang

Corresponding author(s): Yu-Zhong Zhang (zhangyz@sdu.edu.cn) , Lu-Ning Liu (Luning.Liu@liverpool.ac.uk), Peng Wang (wangpeng3331@ouc.edu.cn)

Review Timeline:

Submission Date:	28th Jul 25
Editorial Decision:	9th Sep 25
Revision Received:	10th Jan 26
Editorial Decision:	10th Apr 26
Revision Received:	11th Apr 26
Accepted:	21st Apr 26

Editor: Yehu Moran

Transaction Report:

(Note: With the exception of the correction of typographical or spelling errors that could be a source of ambiguity, letters and reports are not edited. Depending on transfer agreements, referee reports obtained elsewhere may or may not be included in this compilation. Referee reports are anonymous unless the Referee chooses to sign their reports.)

1st Editorial Decision 9th Sep 2025

Dear Prof. Zhang,

Thank you for submitting your manuscript for consideration by the EMBO Journal. It has now been seen by two referees whose comments are shown below.

Given the referees' recommendations, I would like to invite you to submit a revised version of the manuscript, addressing the comments of both reviewers. I should add that it is EMBO Journal policy to allow only a single round of revision, and acceptance of your manuscript will therefore depend on the completeness of your responses in this revised version.

One of the reviewers suspected some irregularities with one of Appendix Figure 17A and reported it to us. This triggered a more rigorous check from our side performed by our Editorial Assistance Team that we usually perform only on a latter stage of the review process. Please see more details below and make sure to address those details in detail in addition to the addressing the referees' comments.

When preparing your letter of response to the referees' comments, please bear in mind that this will form part of the Review Process File, and will therefore be available online to the community. For more details on our Transparent Editorial Process, please visit our website: https://www.embopress.org/page/journal/14602075/authorguide#transparentprocess

We generally allow three months as standard revision time. As a matter of policy, competing manuscripts published during this period will not negatively impact on our assessment of the conceptual advance presented by your study. However, we request that you contact the editor as soon as possible upon publication of any related work, to discuss how to proceed. Should you foresee a problem in meeting this three-month deadline, please let us know in advance and we may be able to grant an extension.

Thank you for the opportunity to consider your work for publication. I look forward to your revision.

Yours sincerely,

Yehu Moran
Academic Editor
The EMBO Journal

Instructions for preparing your revised manuscript:

Please make sure you upload a letter of response to the referees' comments together with the revised manuscript.

Please also check that the title and abstract of the manuscript are brief, yet explicit, even to non-specialists.

When assembling figures, please refer to our figure preparation guideline in order to ensure proper formatting and readability in print as well as on screen:
https://bit.ly/EMBOPressFigurePreparationGuideline
See also guidelines for figure legends: https://www.embopress.org/page/journal/14602075/authorguide#figureformat

At EMBO Press we ask authors to provide source data for the main manuscript figures. You will receive a separate email with instructions for providing source data with your revised manuscript, including how to upload and organize the files.

IMPORTANT: When you send the revision we will require
- a point-by-point response to the referees' comments, with a detailed description of the changes made (as a word file).
- a word file of the manuscript text.
- individual production quality figure files (one file per figure)
- a complete author checklist, which you can download from our author guidelines (https://www.embopress.org/page/journal/14602075/authorguide).
- Expanded View files (replacing Supplementary Information)
Please see out instructions to authors
https://www.embopress.org/page/journal/14602075/authorguide#expandedview
- a Reagents and Tools Table as part of the Methods section, which can be downloaded from our author guidelines (https://www.embopress.org/page/journal/14602075/authorguide#structuredmethods)

Please remember: Digital image enhancement is acceptable practice, as long as it accurately represents the original data and conforms to community standards. If a figure has been subjected to significant electronic manipulation, this must be noted in the figure legend or in the 'Materials and Methods' section. The editors reserve the right to request original versions of figures and the original images that were used to assemble the figure.

Further information is available in our Guide For Authors: https://www.embopress.org/page/journal/14602075/authorguide

We realize that it is difficult to revise to a specific deadline. In the interest of protecting the conceptual advance provided by the work, we recommend a revision within 3 months (8th Dec 2025). Please discuss the revision progress ahead of this time with the editor if you require more time to complete the revisions.

Specific comments by Editorial Assistance Team

*AUTHOR CHECKLIST: missing, please provide.
*PBP: missing, please provide.
*FIGURES IN SEPARATE FILES: at the revision please remove the figures from the manuscript text and upload individual, high resolution figure files. The legends should be placed at the end of the manuscript text.
*Keywords: missing, please provide.
*AUTHORS: email bounced for Ke Fang (fangk8@mail.sysu.edu.cn). Please correct.
*FUNDING: Please add The National Natural Science Foundation of China 32070109, 92251303, Program of Shandong for Taishan Scholars tspd20240806 and tsqn202408064 and the SKLMT Frontiers and Challenges Project (SKLMTFCP-2023-06) to the list of funding sources in our system.
*Author Contributions: Please remove them from the manuscript text and keep them only in our system.
*DisclCIS: Please rename the section "Disclosure and competing interests statement".
*REFERENCE FORMAT: Please correct to alphabetical order and 10 author names listed before et al.
*DATASET EV LEGENDS: Please rename the dataset "Dataset EV1" and add its legend directly to the excel file, in a separate tab/worksheet.
*APPENDIX 1 FILE WITH Table of Contents: Please add page numbers to the table of contents in the file with the supporting information. Please correct the nomenclature to "Appendix Figure S1" and "Appendix Table S1" etc. throughout, and remove the dataset legend as well as the "Other supporting materials for this manuscript include the following: Dataset S1" lines from the file. Please upload the final version of this file as PDF and label it "Appendix".
*REAGENT TABLE: Please upload as a separate file using to template we provide in the Author Guidelines page of the journal.
*SYNOPSIS IMAGE: not provided. Please provide.
*SYNOPSIS TEXT: not provided. Please provide.
*FIGURE CALLOUTS: Appendix Table S5 is called out in the manuscript before Appendix Table S4, and Appendix Figures S9 and 10 are called out before Appendix Figures S7 and 8; Appendix Figure S15 is called out before Appendix Figure S11. Please correct.

*FIGURE CHECK:

Appendix Fig. 17A - GAPDH figure has pixelation, which might have triggered concerns from the referee (see below).
Please provide source data for this figure. This is extremely important at this point.

A referee mentioned that - "the images of the 10 lanes have been squeezed to fit to the size of the image of the 9 lanes. This is obviously not meaningful, and clearly a breach against any good scientific practice."
While we are unable to confirm this concern, if this has been done, the authors need to clearly show this with demarcation lines and provide explanation for why something like this was done. Please address this point in detail in your response to us.

Notes:
- Please remove the section "Additional information" from the manuscript text and update the order and headings of the manuscript sections to the following:
Abstract / Keywords / Introduction / Results / Discussion / Methods / Data Availability / Acknowledgements / Disclosure and Competing Interests Statement / References / Main Figure Legends / Tables / Expanded View Figure Legends

DATA CHECK: Problematic
- Data Availability Statement:
1. Please note that the specific URLs for 8ZGJ, EMD-60080, 8ZGK, EMD-60081, 8ZGL, EMD-60082, 8ZGN, and EMD-60084 datasets are not provided in the data availability statement.
2. Please note that reviewer access codes for 8ZGJ, EMD-60080, 8ZGK, EMD-60081, 8ZGL, EMD-60082, 8ZGN, and EMD-60084 datasets are not provided in the data availability statement.
- Figure legends:
1. Please note that the exact p values are not provided in the legends of figures 1E, F; 3E, 4D, E

2. Please note that the measure of center for the error bars needs to be defined in the legends of figures 1B, C, E, F, G; 3E, 4D, E

Comments by Referees:

Referee #1:

The authors identified and systematically characterized the transporter DddT from the marine gamma-proteobacterium Psychrobacter sp. D2 structurally and functionally. DddT belongs to the BCCT-family of transporters, which contains the well characterized prototypic member BetP. The study shows that DddT transports Dimethylsulfoniopropionate (DMSP) in a sodium-dependent manner, suggesting a function as Na+-coupled symporter for DMSP uptake. Understanding DddT mechanism is of broader interest, as the microbial catabolic product of DMSP, dimethylsulfide, is important in the global sulfur cycle and as climate-active gas. The authors determined cryo-EM structures of the recombinant trimeric DddT in closed substrate-free state in presence of sodium ions, in closed DMSP-bound state, in closed state in presence of potassium ions, and of variant G101D (a substrate-free outward open state) at overall resolution of 2.8 Å, 2.98 Å, 2.94 Å, and 2.98 Å respectively. DMSP and sodium binding sites were described, and probed by mutational analysis combined with in vivo functional assays using heterologous expression in E.coli. Combined with molecular dynamics simulations to analyse conformational changes between states, a model for the transport mechanism was derived. A phylogenetic analysis of DddT genes clustered with DMSP lyase genes analysed the distribution of homologous transporters in bacterial genomes. In summary, it is a very comprehensive study with extensive structure/function analysis. Overall, the experimental data are sound, yet, the interpretation does not fully justify the conclusions drawn. Especially, the experimental evidence for the derived mechanism, which the authors describe as different to other members of the BCCT-familiy, is to large extent based on a proposed sodium binding site, for which experimental evidence is not convincing. Another major concern about the study lies in the functional characterization of the dddT transporter. If DMSP concentration in seawater is 10 - 200 nM, the reported slow growth and transport rates and high Km values would mean very poor uptake of DMSP, so the biological relevance is not clear.
Minor comments:
Line 63 - information regarding the typical concentration of DMPS in seawater should be added
Line 107/F1b,c - the P. sp. D2 growth is very slow with 6 mM DMSP as sole carbon source. Information about growth on other carbon sources should be provided.
Line 114/Fig. 1d - in the text, produced higher levels of DMS (not DMSP); in the figure, the intensity read-out of the detector is given. What is the concentration of DMS in the culture medium samples?
Line 120 - would be good to indicate strength of promoter, strong promoter?
Line 122 Fig. 1e/f - the uptake rates are very low as compared for instance to five order of magnitude higher rates of BetP in similar experiment. Information on comparable transporters should be provided and low rates discussed.
Line 126/Fig. 1g - estimated apparent Km for in vivo uptake rates in E. coli is not justified, there are no data points below the derived Km value
Line 129 ff, description of overall structure of DddT based on closed substrate-free state at an overall resolution of 2.8A. Include information in text whether C3 symmetry was enforced. If not imposed, are there any differences between protomers?
Line 168 - dynamics were not analyzed, so statement that G101D structure "enabled examination of opening and closing dynamics" should be deleted. The structure of the variant resolved a substrate-free outward open state.
Line 174ff /S14 - to be able to judge ligand binding, cryo-EM map in supplementary figure should be also shown for environment of DMSP. The same holds true for the tentatively assigned sodium ions. Coordinating side chains of ligand and ions should be analyzed and shown.
Line 176 - sentence not complete
Line 203 - Fig. S17 - There is a gross error in this figure. There are 10 lanes in the GADPH control, but only 9 lanes for DddT, for all three sections. In addition, the variation in DddT signal between experimental batches is substantial. It is not clear from the legend, which states: "independent batches, each of which may exhibit variability due to differences in sample loading, incubation time, and other experimental conditions", whether experimental conditions in one batch are the same to justify the chosen type of analysis. Minor point: Number of digits are not appropriate, and ordering the data along amino acid sequence would make it much easier to find a data point and compare to data from main text/figures.
Line 206 - it is great that all the mutants were generated and analysed. I would be much easier for the reader if the mutants would be ordered according to sequence, and also to integrate information regarding expression level (S17), otherwise one has to jump back and forth to find out whether low uptake rate is connected to lack of expression. Asp99 for instance, seems to have substantially lower expression level than WT in that experiment.
Line - 216 Figure 3e (not 3h)
Fig 3f - The density assigned to DMSP is not unambiguous for the assignment. Contour level of the map should be given in R.M.S.D., and all distances of hydrogen bonds should be included. What are the distances of the modelled DSMP to W321 and to W320?
Line 226 - Fig. 4a the sodium binding site Na2, which is conserved with BetP appears to have an appropriate coordination including distances. The coordination of the second potential sodium binding site (Na1) with given geometry and distances mainly above 3 A up to 5.9 A appears very unlikely.
Line 236 - good to explain the rational for proline and alanine substitutions, but it would be helpful to refer precisely to mutation in the text (e.g. lines 241/242), as it is really difficult to derive from the figures whether coordination is from main chain or side

chain atom (e.g. T419, S422, ...)
Line 238 ff - experiments in 4d have not been performed under various sodium gradients, these are DMSP uptake assays under saturated sodium concentration. As TM7 and TM10 were both described to adopt altered conformation in different states (line 160 ff), the reduced transport activity could also derive from hampering the conformational change.
Line 239 - have the expression levels of these mutants been analysed?
Line 240 - is the stoichiometry of DMSP/Na symport for DddT known?
Line 252 ff / Fig. 5 - from the description of the text and the Fig 5 it is not fully clear, which steps of the proposed transport mechanism are supported by the cryo-EM structures or assigned to them , or derived also from MD simulations performed in this study.
Line 283 ff, as outlined above, the Na1 binding site is questioned. Computational approaches/molecular dynamic simulations would be important to support the analysis. In the following paragraph on the DMSP transport mechanism (line 306ff), MD simulation were performed. Binding of sodium ions binding to Na1 is described in the text (line 318), but data are not shown.
Fig. 6: Line 809: Which algorithm was used to randomly select the 20 sequences for multiple sequence alignment?
Fig. S4 (the same for S7, S9): The authors described that sharpening were performed to obtain a map with a resolution of 2.8 Å. The resolution should be calculated with unsharpened maps. See also line 513: sharpening should not be part of the refinement scheme.
Fig. S10: From the figure, it is not clear where the DMSP molecule is located, as the molecule is quite small as compared to the protein part shown and the color used is identical to the surrounding polypeptide.
Fig. S11: cryoEM map enclosing D101 should be shown with contour level.
Fig. S14: The contour level of the map enclosing DMSP should be given (with the unit R.M.S.D.). The map enclosing Na1 and Na2 should be shown at the same contour level as that for DMSP. Provide a detailed view of DMSP with map, and show its interaction (hydrogen bond, hydrophobic interactions ...etc) partners of the protein with corresponding distances.
Fig. S15. The unit of RMSD should be given and also information about comparison (main-chain, all atom, ..).
Fig. S16: Rational of assigning sodium ions and not water molecules to cryo-EM maps should be given. How was the assignment of sodium ions justified? The map feature of Na1 in the CsS state appears to be very weak. At this resolution (about 3 Å) and without the coordination sphere it is not convincing that it can be assigned to a sodium ion. It is not clear if the contour level indicated in the figure is arbitrary. The description of the contour level should be denoted using the unit of R.M.S.D..
Fig. S19: The definition of grey box, blue box and pink box is unclear. It is also unclear which exact residues are described as hydrophilic and hydrophobic in these boxes therefore this figure is not comprehensible.
Fig. S20. The name of the species are illegible. The color code of the circle should be noted.
Line 550: the hidden Markov model is typically constructed from a multiple sequence alignment to sample the diverse sequence variability. Here only two sequences were used. Explain why.

Referee #2:

In this report by Zhang, Zhu, Li and colleagues, the authors report structures of the Na-coupled DMSP transporter from a marine gamma-proteobacterium in various states. DddT adopts a trimeric architecture with each of the three protomers resembling BetP, the prototypical BCCT transporter. The structures of BCCT revealed the substrate-binding site as well as two Na binding sites, one of which is distinct from the Na-binding sites observed in other BCCT transporters. The role of the proposed binding sites in DMSP transport are interrogated using mutagenesis. Combing the structural data with mutagenesis and molecular dynamics simulations, the authors propose a transport mechanism for DddT. While the data appears to be of high quality, several of the interpretations are not well supported by the data and should be revised prior to publication.

Comments.
1. Have any measurements been made characterizing the stoichiometry of symport for DddT or any other related DMSP transporters?
2. A more detailed description of the DMSP binding site should be included in the text. This description should be accompanied by a detailed presentation in Figure 3. The small inset in panel h is hard to interpret and it is unclear how specific residues interact with the substrate. These are key features of the transporter and should be clearly presented.
3. The modelled bond distances for several residues that are assigned as interacting with the proposed Na1 site are too far to directly interact with a bound Na. The geometry of the binding site should be carefully evaluated. This is particularly important for the CcS structure in which the density of the proposed ion binding site is rather weak.
4. Proline substitutions can have effects beyond altering the amino group of a peptide bond and thus care must be taken in interpreting their effects, particularly for residues near substrate-binding sites.
5. The resolution of the reported structures is too low to support the claim that "Chemically and structurally, the interaction of the DMSP molecule with the amino group of the peptide bond can affect the electron cloud distribution of the peptide bond, thereby enhancing the ability of oxygen to bind metal ions and stabilize the conformation of the peptide bond." If this statement is supported by other reports, they should be cited, and the context should be clarified.
6. Several statements are similarly supported only weakly by the data and need to be revised: "Similarly, Na2 binding probably enhanced the interaction between the amino group of the peptide bond and the carboxyl group of DMSP, thereby stabilizing DMSP binding. Our results for the Cc-K+ state confirm this point, showing that in the absence of Na+, the addition of DMSP only results in the formation of the Cc state conformation without achieving the CcS state conformation." Structural evidence should

be presented to support an enhanced interaction between the amino group and the carboxyl of DMSP. The absence of a state in a cryo-EM analysis (in this case the Cc state) cannot inform on the effect of ligands on the conformational landscape when ~90% of the particles are excluded during data processing.
7. Affinities cannot be assessed from the bond lengths determined from cryo-EM reconstructions. Please revise or remove the statements "Meanwhile, the binding affinity of the Na1 site for sodium ions appears to be reduced, as evidenced by the increased length of hydrogen bonds at the Na1-binding site in the CcS state." "During the transport process, the binding affinity for Na1 appears to decrease, potentially leading to the release of Na1 in the CcS state."
8. Please clearly define in the discussion the evidence that the transport of DMSP and Na are cooperative.
9. A single molecular dynamics simulation is insufficient to presented as evidence supporting a transport model. The authors should be repeat the simulations multiple times and use statistical models to ensure that their interpretations are supported by the results.

Minor comments.
1. It would be helpful to have the full name (Dimethylsulfoniopropionate) in the title of the report to help the broad and general audience of EMBO Journal.
2. The concentration of Na used to measure the Km in Figure 1g should be reported in the figure legend to aid interpretation.

Responses to Reviewers

Line numbers refer to the PDF file generated by the submission system. In addition, a version of the manuscript with revisions highlighted is provided.

Reviewer 1:

1. The authors identified and systematically characterized the transporter DddT from the marine gamma-proteobacterium *Psychrobacter* sp. D2 structurally and functionally. DddT belongs to the BCCT-family of transporters, which contains the well characterized prototypic member BetP. The study shows that DddT transports Dimethylsulfoniopropionate (DMSP) in a sodium-dependent manner, suggesting a function as Na^+-coupled symporter for DMSP uptake. Understanding DddT mechanism is of broader interest, as the microbial catabolic product of DMSP, dimethylsulfide, is important in the global sulfur cycle and as climate-active gas. The authors determined cryo-EM structures of the recombinant trimeric DddT in closed substrate-free state in presence of sodium ions, in closed DMSP-bound state, in closed state in presence of potassium ions, and of variant G101D (a substrate-free outward open state) at overall resolution of 2.8 Å, 2.98 Å, 2.94 Å, and 2.98 Å respectively. DMSP and sodium binding sites were described, and probed by mutational analysis combined with in vivo functional assays using heterologous expression in *E. coli*. Combined with molecular dynamics simulations to analyse conformational changes between states, a model for the transport mechanism was derived. A phylogenetic analysis of DddT genes clustered with DMSP lyase genes analysed the distribution of homologous transporters in bacterial genomes. In summary, it is a very comprehensive study with extensive structure/function analysis.

Response: Thank you very much for the highly positive comments. We have carefully revised the manuscript according to your suggestions. We believe that these revisions have substantially improved the quality of the manuscript. Our detailed responses to your concerns are provided below.

2. Overall, the experimental data are sound, yet, the interpretation does not fully justify the conclusions drawn. Especially, the experimental evidence for the derived mechanism, which the authors describe as different to other members of the BCCT-familiy, is to large extent based on a proposed sodium binding site, for which experimental evidence is not convincing.

Response: Thank you for this valuable comment. We agree that stronger experimental evidence is required for the proposed transport mechanism, particularly the identification and functional relevance of the newly proposed sodium-binding site (Na1). To address this, we have performed additional structural and functional experiments, as detailed below.

1) Improved cryo-EM maps

We re-collected and reprocessed the cryo-EM datasets for both the C_cS and C_c states, improving their overall resolutions to 2.52 Å and 2.69 Å, respectively. In the refined maps, the density corresponding to the Na1 site is more clearly defined, and an additional coordinating water molecule can now be resolved (Figure 4B). This improvement also allowed correction of the previously measured coordination distances (Figure 4B). Although the Na1–ligand distances in DddT remain slightly longer than those typically observed for tightly bound Na^+ ions, we consider them reasonable and consistent with a relatively labile, transient binding site. Na^+-binding sites in transporters can exhibit non-canonical coordination. For example, in the

outward-occluded structure of LeuT, the identified Na1 site also exhibits longer coordination distances (Nature. 2012, 481:469-74), and in the BetP transporter, the proposed Na1 site displays a coordination pattern distinct from that of the stable Na2 site (Nat Commun, 2014, 5:4231). We consider this feature to be functionally relevant. Specifically, the identification of Na1 in our cryo-EM structures is likely facilitated by the high Na^+ concentrations present on both the periplasmic and cytoplasmic sides during sample preparation, which favors Na1 occupancy. Under physiological conditions, the lower cytoplasmic Na^+ concentration may instead promote Na1 dissociation, potentially influencing Na2 binding and the progression of the transport cycle. The clarification has been added to the revised manuscript (Lines 284-296, 323-331).

2) Structural validation under Na^+-free conditions

To further validate the assignment of the Na1 density, we collected cryo-EM data for the G101D mutant in a Na^+-free buffer. Under these conditions, the density corresponding to the Na1 site completely disappeared (Figure EV1 and Appendix FigureS15). Consistently, in the C_c-K^+ state structure of DddT, the corresponding Na1 density was also absent when the protein was prepared in a Na^+-free buffer (Figure EV1 and Appendix Fiure S10). Together, these observations strongly support the conclusion that the previously observed density at the Na1 site represents a specifically bound Na^+ ion rather than a water molecule or another cation.

Notably, under Na^+-free conditions, the G101D mutant adopts an inward-facing (C_i) conformation of DddT. This newly obtained structure fills a previous gap in our structural understanding of the inward-open state and suggests a possible allosteric coupling between Na^+ binding/release and the inward–outward conformational transitions of DddT. We have discussed this in the revised manuscript (Lines 230-238).

3) Transport stoichiometry

We further quantified Na^+ and DMSP fluxes to determine the transport stoichiometry of DddT. These measurements reveal a coupling ratio of approximately 2 Na^+ ions per DMSP molecule (Appendix Figure S18, Lines 344-347). This result supports the presence of two functional Na^+ binding sites (Na1 and Na2) and contradicts a transport mechanism mediated solely by a single Na2 site.

4) Comparison with other transporter families

We note that a Na^+ binding site equivalent to Na1 has not been previously resolved in available BCCT family structures. However, a Na^+-binding site located in a comparable region has been reported in members of the LeuT superfamily, although the coordinating residues differ (Nat Commun, 2018, 9:1753), providing independent precedent for the existence of such a site.

Together with our previously reported mutational analyses, these new structural and functional data provide substantially strengthened evidence supporting the presence and mechanistic relevance of the Na1 site. We have revised the manuscript accordingly (Lines 230-238, 284-296, 323-331, and 344-347, Figures 4B and EV1). Moreover, we have toned down the wording regarding differences between the transport mechanisms of DddT and BetP. Rather than proposing distinct transport mechanisms, we now state that DddT and BetP possess different Na1-binding sites, which may lead to differences in their transport processes. In contrast, the Na2 site, which is essential for transport, is conserved and plays a central

functional role in both systems (Lines 364-365).

3. Another major concern about the study lies in the functional characterization of the *dddT* transporter. If DMSP concentration in seawater is 10 - 200 nM, the reported slow growth and transport rates and high K_m values would mean very poor uptake of DMSP, so the biological relevance is not clear.

Response: Thank you for this important comment. We agree that the relatively high K_m values and slow apparent transport rates raise questions regarding the physiological relevance of DddT under average seawater DMSP concentrations. As suggested, we re-measured the K_m value of DddT, which indeed remains higher than typical bulk oceanic DMSP concentrations (Figure 1G). Nevertheless, multiple lines of evidence indicate that DddT is biologically relevant under its native environmental conditions.

1) Although DddT exhibits relatively high apparent K_m values compared with bulk seawater DMSP concentrations, localized microenvironments such as phytoplankton phycospheres and decaying algal particles can reach much higher DMSP levels, representing one of the common habitats for *Psychrobacter* species (Nat Commun, 2020, 11:1942; ISME J, 2024, 18:ware130; Syst Appl Microbiol, 2004, 27:399-406; Nat Rev Earth Environ, 2023, 4:361-376; Biosci Biotechnol Biochem, 2024, 88:1487-1495; Geophys Res Lett, 2019, 46:12279-12288; Sci China Life Sci, 2019, 62:1296-1319). Under such conditions, the local DMSP concentrations are likely sufficient to support DddT-mediated uptake, despite its relatively high K_m.

2) Transcriptomic analyses show that *dddT* expression is strongly induced in the presence of DMSP (eLife, 2021, 10:e64045), indicating that DddT is not constitutively expressed but is specifically deployed when DMSP is available. This inducible expression feature supports a physiological role for DddT in DMSP acquisition and metabolism under relevant environmental conditions.

3) Although the strain D2 exhibited relatively slow growth when DMSP was provided as the sole carbon source, this phenotype cannot be directly attributed to inefficient DMSP uptake. Indeed, we examined the growth of strain D2 on several defined media containing commonly used carbon sources as suggested. The results showed even slower growth rates were observed on other typical single carbon sources (Appendix Figure S2). Together, these results suggest that the limited growth rate is more likely attributed to the lower utilization efficiency of a single organic substrate, rather than being solely attributed to poor DMSP uptake efficiency per se.

4) To confirm that DddT functions as a major DMSP transporter, rather than to quantitatively reflect in situ uptake rates, we performed DMSP transport assays in strain D2 using cells grown to mid-log phase in nutrient-rich 2216E medium. As growth experiments revealed that strain D2 does not preferentially utilize DMSP in the nutrient-rich 2216E medium (Appendix Figure S2), the measured transport rates are therefore likely to underestimate the physiological activity of DddT under natural conditions.

We have revised the manuscript (Lines 119-121, 150-161) to clarify the ecological context of DddT function and to better explain the biological relevance.

Minor comments:

4. Line 63 - information regarding the typical concentration of DMSP in seawater should be added

Response: The typical concentration of DMSP in seawater and references have now been added. In bulk seawater, DMSP concentrations are typically in the range of 10–200 nM; however, substantially higher concentrations, often reaching the micromolar range, can be found in localized microenvironments such as phytoplankton phycospheres and decaying algal particles (Lines 55-57).

5. Line 107/F1b,c - the *P*. sp. D2 growth is very slow with 6 mM DMSP as sole carbon source. Information about growth on other carbon sources should be provided.

Response: Information about growth on other carbon sources have been provided (Appendix Figure S2).

6. Line 114/Fig. 1d - in the text, produced higher levels of DMS (not DMSP); in the figure, the intensity read-out of the detector is given. What is the concentration of DMS in the culture medium samples?

Response: Thanks for this comment. DMS in our assay originates exclusively from the cleavage of DMSP by bacterial DMSP lyases. Once cells take up DMSP, a fraction is transformed into DMS, so strains that import DMSP more rapidly emit proportionally greater amounts of DMS. This parameter thus provides an indirect measure supporting the transport activity of DddT. In our experiment, any minor background DMS generated by spontaneous DMSP degradation was measured in DMSP-only controls and subtracted as the baseline. DMS is volatile, it was completely purged from the liquid phase and trapped for quantification, ensuring that the measured signal represents the total DMS released from the culture. The concentration of DMS has now been quantified and added to Figure 1D.

7. Line 120 - would be good to indicate strength of promoter, strong promoter?

Response: Thank you for the suggestion. We have now indicated the strength and characteristics of the promoter in the revised manuscript. The sentence has been updated to read: "To further study the activity of DddT, *P.* sp. D2 *dddT* was cloned into the pET-22b vector, with its promoter replaced by the constitutive promoter BBa-J23111, a moderate-strength promoter widely used for stable gene expression in *E. coli.*" (lines 140-142).

8. Line 122 Fig. 1e/f - the uptake rates are very low as compared for instance to five order of magnitude higher rates of BetP in similar experiment. Information on comparable transporters should be provided and low rates discussed.

Response: Thank you for this insightful comment. We assessed DddT-mediated DMSP uptake using two approaches. First, we measured the uptake rate directly in strain D2 cells (~0.05 nmol min^{-1} mg^{-1} cdw, Figure 1E). Cells were grown to mid-log phase in nutrient-rich 2216E medium,

and the assays were designed primarily to confirm that DddT functions as a DMSP transporter, rather than to quantitatively reflect in situ uptake rates. In rich media such as 2216E, strain D2 did not preferentially utilize DMSP; therefore, the measured transport rates likely underestimate the physiological activity of DddT under natural conditions. Second, we performed uptake assays in *E. coli*, using a setup similar to that for BetP, mainly to examine the effect of different sodium gradients on transport (Figure 1F). The measured uptake rate was ~0.25 nmol min^{-1} mg^{-1} cdw, approximately 1/300 of the reported rate for BetP transporting betaine (~83 nmol min^{-1} mg^{-1} cdw; Nat Commun, 2014, 5:4231). While numerically lower, it is important to note that uptake rates normalized per mg bacterial dry weight can be remarkably influenced by differences in expression level, folding efficiency, and membrane insertion of the transporter in the heterologous *E. coli* system. Therefore, the observed numerical difference may not necessarily indicate a biologically relevant disparity. We have discussed this point in the revised manuscript (Lines 154–158).

9. Line 126/Fig. 1g - estimated apparent K_m for in vivo uptake rates in *E. coli* is not justified, there are no data points below the derived K_m value

Response: Thanks for this important comment. We have re-measured K_m and updated the manuscript with the new values (Line 149, Figure 1G).

10. Line 129 ff, description of overall structure of DddT based on closed substrate-free state at an overall resolution of 2.8A. Include information in text whether C3 symmetry was enforced. If not imposed, are there any differences between protomers?

Response: Thank you for this helpful suggestion. We have clarified this point in the revised manuscript (Lines 174-177). Briefly, C3 symmetry was not imposed during the initial refinement. The map was first reconstructed in C1, and the three protomers were compared, revealing no notable conformational differences. Based on this observation, C3 symmetry was subsequently applied to improve map quality and final resolution.

11. Line 168 - dynamics were not analyzed, so statement that G101D structure "enabled examination of opening and closing dynamics" should be deleted. The structure of the variant resolved a substrate-free outward open state.

Response: We agree with you and have deleted the statement that the G101D structure “enabled examination of opening and closing dynamics” (Lines 209-210).

12. Line 174ff /S14 - to be able to judge ligand binding, cryo-EM map in supplementary figure should be also shown for environment of DMSP. The same holds true for the tentatively assigned sodium ions. Coordinating side chains of ligand and ions should be analyzed and shown.

Response: Thanks for this suggestion. We have now provided cryo-EM density maps for the environment of DMSP and the tentatively assigned sodium ions in the revised Figures 3E and

4B. In addition, the coordinating side chains for both the ligand and ions have been analyzed and shown.

13. Line 176 - sentence not complete

Response: The sentence has been revised to ensure clarity and completeness. It now reads: "There is a change in the transmembrane helix orientation when DMSP and Na^+ are bound." (Line 216).

14. Line 203 - Fig. S17 - There is a gross error in this figure. There are 10 lanes in the GAPDH control, but only 9 lanes for DddT, for all three sections. In addition, the variation in DddT signal between experimental batches is substantial. It is not clear from the legend, which states: "independent batches, each of which may exhibit variability due to differences in sample loading, incubation time, and other experimental conditions", whether experimental conditions in one batch are the same to justify the chosen type of analysis. Minor point: Number of digits are not appropriate, and ordering the data along amino acid sequence would make it much easier to find a data point and compare to data from main text/figures.

Response: Thank you for carefully examining this figure and for these constructive comments. We sincerely apologize for the confusion caused by the earlier version. The mismatch in lane numbers (10 Anti-GAPDH lanes versus 9 Anti-His lanes) resulted from an inadvertent omission of the empty control lane during figure assembly. We have now corrected Appendix Figure S17 (formerly Fig. S17) to ensure a one-to-one correspondence between Anti-His and Anti-GAPDH lanes, and the updated figure together with its full source data have been provided (Source Data Appendix Figure S17A, Figure R1 below).

Regarding the variation in DddT signal intensity among experimental batches, we agree that our previous legend text was unclear. We have revised the legend to explicitly state that all batches were performed under identical experimental conditions; however, unavoidable operational variability among batches (including incubation time, and other small procedural fluctuations) can lead to differences in signal amplitude. To address this, each batch included its own internal control.

Regarding the ordering of data along the amino acid sequence, we agree that this would improve visual accessibility. However, because the current results are based on blots in which the sample arrangement was predetermined, reordering is not feasible without repeating the entire experiment, which we consider not crucial for supporting the conclusion. Consequently, we have not reordered the data in the present version. Should you consider this point critical, we would be happy to repeat the experiment and revise the ordering accordingly.

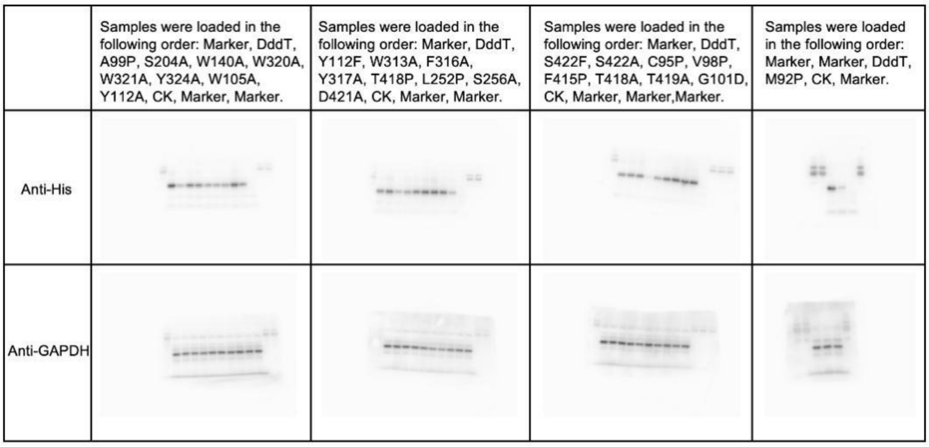

Figure R1. Western blot data of empty control, DddT, and its mutants. The sample loading order is indicated in the figure.

15. Line 206 - it is great that all the mutants were generated and analysed. I would be much easier for the reader if the mutants would be ordered according to sequence, and also to integrate information regarding expression level (S17), otherwise one has to jump back and forth to find out whether low uptake rate is connected to lack of expression. Asp99 for instance, seems to have substantially lower expression level than WT in that experiment.

Response: Thank you very much for this helpful suggestion. We apologize for any confusion caused by the previous figure presentation. The uptake activities of all DddT mutants shown in the main figures have already been normalized to their respective expression levels, as determined by western blots. We have now clarified this explicitly in the updated figure and legend to ensure that readers understand that differences in uptake reflect functional effects rather than variations in protein abundance (Figures 3F, 4D and E).

The order of mutants in the main figures currently corresponds to the western blot sample layout to maintain consistency. Please see our response above regarding the ordering of data along the amino acid sequence.

16. Line - 216 Figure 3e (not 3h)

Response: Thank you for pointing this out. We have carefully checked and corrected all figure references in the revised manuscript to ensure that they accurately correspond to the appropriate panels.

17. Fig 3f - The density assigned to DMSP is not unambiguous for the assignment. Contour level of the map should be given in R.M.S.D., and all distances of hydrogen bonds should be included. What are the distances of the modelled DSMP to W321 and to W320?

Response: Thank you for this comment. In the revised manuscript, we have specified the contour level of the cryo-EM map in RMSD (Lines 852-855). We have also measured and included all relevant hydrogen-bond distances and cation–π interaction distances between the modeled DMSP and surrounding residues, including those from W321 and W320 to DMSP in Figure 3E (previously Figure 3f). This result has been added to the revised manuscript (Lines 258-261).

18. Line 226 - Fig. 4a the sodium binding site Na2, which is conserved with BetP appears to have an appropriate coordination including distances. The coordination of the second potential sodium binding site (Na1) with given geometry and distances mainly above 3 A up to 5.9 A appears very unlikely.

Response: Thank you for this insightful comment. In the revised manuscript, we have provided additional supporting data for the potential Na1 site, as responded to Comment 2 above, and have corrected the reported coordination distances; all distances are now within 4 Å. Although these distances are slightly longer than those observed in canonical sodium-binding sites, we consider Na1 not to be a high-affinity, canonical binding site, but rather a site that functions transiently during the transport cycle. Na^+-binding sites in transporters can exhibit non-canonical coordination. For example, in the outward-occluded structure of LeuT, the identified Na1 site also exhibits longer coordination distances (Nature, 2012, 481:469-74), and in the BetP transporter, the proposed Na1 site displays a coordination pattern distinct from that of the stable Na2 site (Nat Commun, 2014, 5:4231). The identification of Na1 in our cryo-EM structures is likely facilitated by the high Na^+ concentrations on both the periplasmic and cytoplasmic sides during sample preparation, which favor Na1 occupancy. Under physiological conditions, the lower cytoplasmic Na^+ concentration may instead promote Na1 dissociation, potentially influencing Na2 binding and the progression of the transport cycle.

19. Line 236 - good to explain the rational for proline and alanine substitutions, but it would be helpful to refer precisely to mutation in the text (e.g. lines 241/242), as it is really difficult to derive from the figures whether coordination is from main chain or side chain atom (e.g. T419, S422, ...)

Response: Thank you for this great comment. We have revised the text to explicitly refer to the specific mutations, including the corresponding lines in the manuscript (Lines 284-289).

20. Line 238 ff - experiments in 4d have not been performed under various sodium gradients, these are DMSP uptake assays under saturated sodium concentration. As TM7 and TM10 were both described to adopt altered conformation in different states (line 160 ff), the reduced transport activity could also derive from hampering the conformational change.

Response: We would like to clarify that DMSP uptake assays under varying sodium concentrations have already been performed, as shown in the new Figure 4D (previously Figure 4e).

21. Line 239 - have the expression levels of these mutants been analysed?

Response: Yes, the expression levels of these mutants have been analyzed, and the results are included in Figures 4C, D and Appendix Figure S17.

22. Line 240 - is the stoichiometry of DMSP/Na symport for DddT known?

Response: The stoichiometry of DMSP/Na^+ symport for DddT was previously unknown. We have now measured it using solid-supported membrane (SSM) electrophysiology, and the results indicate a stoichiometry of 2 Na^+ ions per DMSP molecule. These results have been incorporated into the revised manuscript (Lines 344-347, Appendix Figure S18).

23. Line 252 ff / Fig. 5 - from the description of the text and the Fig 5 it is not fully clear, which steps of the proposed transport mechanism are supported by the cryo-EM structures or assigned to them, or derived also from MD simulations performed in this study.

Response: Building on our existing results, we have now obtained a new structure in the inward-facing conformation. Consequently, the MD simulation results that were previously used to model this conformation have been removed. The legend of Figure 5 has been revised to clearly indicate that all steps of the proposed transport mechanism are directly supported by the cryo-EM structures.

24. Line 283 ff, as outlined above, the Na1 binding site is questioned. Computational approaches/molecular dynamic simulations would be important to support the analysis. In the following paragraph on the DMSP transport mechanism (line 306ff), MD simulation were performed. Binding of sodium ions binding to Na1 is described in the text (line 318), but data are not shown.

Response: Thank you for this important comment. We have now provided additional experimental evidence to support the assignment of the Na1-binding site in our responses to Comment 2 above.

25. Fig. 6: Line 809: Which algorithm was used to randomly select the 20 sequences for multiple sequence alignment?

Response: Thank you for the question. After taxonomic classification of all homologous DddT sequences, we performed random sampling within each clade to ensure balanced representation. This has been clarified in the revised manuscript (Lines 897-898).

26. Fig. S4 (the same for S7, S9): The authors described that sharpening were performed to obtain a map with a resolution of 2.8 Å. The resolution should be calculated with unsharpened maps. See also line 513: sharpening should not be part of the refinement scheme.

Response: We apologize for the inaccurate descriptions. Sharpening was only applied after refinement, and the resolution values were calculated from unsharpened maps. The manuscript and workflow diagram have been corrected accordingly (Appendix Figures S6, S8, S10, S13, and S15, Lines 576-579).

27. Fig. S10: From the figure, it is not clear where the DMSP molecule is located, as the molecule is quite small as compared to the protein part shown and the color used is identical to the surrounding polypeptide.

Response: In the revised figure, we have enlarged the DMSP molecule and recolored it using a distinct color scheme to differentiate it clearly from the surrounding protein. The revised figure has been incorporated into Figure 3E.

28. Fig. S11: cryoEM map enclosing D101 should be shown with contour level.

Response: We have now added the contour level information to the cryo-EM map enclosing D101 (Figure EV2).

29. Fig. S14: The contour level of the map enclosing DMSP should be given (with the unit R.M.S.D.). The map enclosing Na1 and Na2 should be shown at the same contour level as that for DMSP. Provide a detailed view of DMSP with map, and show its interaction (hydrogen bond, hydrophobic interactions ...etc) partners of the protein with corresponding distances.

Response: As suggested, the figure has been revised to report the contour level (R.M.S.D.) for the DMSP map, and Na1/Na2 are now shown at the same contour level. In addition, a zoomed-in view of DMSP with interacting residues and distances has been added, and the revised figure has been incorporated into Figure 3E.

30. Fig. S15. The unit of RMSD should be given and also information about comparison (main-chain, all atom, ..).

Response: Thank you for the comment. The unit of RMSD and the type of comparison have now been clearly indicated in Appendix Figure S12 (previously Fig. S15).

31. Fig. S16: Rational of assigning sodium ions and not water molecules to cryo-EM maps should be given. How was the assignment of sodium ions justified? The map feature of Na1 in the CsS state appears to be very weak. At this resolution (about 3 Å) and without the coordination sphere it is not convincing that it can be assigned to a sodium ion. It is not clear if the contour level indicated in the figure is arbitrary. The description of the contour level should be denoted using the unit of R.M.S.D..

Response: Thank you for this important comment. As responded to Comment 2 above, we have provided additional experimental evidence to support the assignment of the Na1-binding site. In particular, we have re-collected and reprocessed the cryo-EM datasets for both the C_cS and C_e states, improving the map resolutions to 2.52 Å and 2.69 Å, respectively. In the refined maps, the density at the Na1 site is more clearly resolved, and a weak additional density attributable to a coordinating water molecule can now be observed (Figure 4B). Moreover, in newly determined Na^+-free structures (C_i and C_c-K^+), the corresponding density at this position is absent, further supporting its assignment as Na^+ rather than water (Figure EV1). The contour levels in the revised Figure 4B are now consistently reported in R.M.S.D. units and set to 8 σ.

32. Fig. S19: The definition of grey box, blue box and pink box is unclear. It is also unclear which exact residues are described as hydrophilic and hydrophobic in these boxes therefore this figure is not comprehensible.

Response: Thank you for the valuable suggestion. To improve clarity, we have revised the legend and figure to explicitly define the color-coded boxes and identify the hydrophilic and hydrophobic residues. Hydrophilic residues are now shown in green and hydrophobic residues in orange. These changes have been incorporated into Figure EV3 (previously Fig. S19) to enhance the comprehensibility of the alignment.

33. Fig. S20. The name of the species are illegible. The color code of the circle should be noted.

Response: In the revised Figure EV4 (previously Fig. S20), the resolution has been substantially improved to enhance legibility, and the color code of the circles is now indicated in the figure legend.

34. Line 550: the hidden Markov model is typically constructed from a multiple sequence alignment to sample the diverse sequence variability. Here only two sequences were used. Explain why.

Response: Thank you for this comment. Only these two sequences were selected to ensure the rigor and reliability of the analysis. These sequences are the only experimentally validated ones that are both active and sufficiently diverse, while belonging to the same phylogenetic branch and containing conserved Na1 and Na2 binding sites. Therefore, they are the most representative DddT homologues. Although this is fewer than the number of sequences typically used for HMM construction, these two sequences capture the essential conserved features relevant for our intended analysis.

Reviewer 2:

1. In this report by Zhang, Zhu, Li and colleagues, the authors report structures of the Na-coupled DMSP transporter from a marine gamma-proteobacterium in various states. DddT adopts a trimeric architecture with each of the three protomers resembling BetP, the prototypical BCCT transporter. The structures of BCCT revealed the substrate-binding site as well as two Na binding sites, one of which is distinct from the Na-binding sites observed in other BCCT transporters. The role of the proposed binding sites in DMSP transport are interrogated using mutagenesis. Combing the structural data with mutagenesis and molecular dynamics simulations, the authors propose a transport mechanism for DddT. While the data appears to be of high quality, several of the interpretations are not well supported by the data and should be revised prior to publication.

Response: We sincerely thank you for your careful evaluation and constructive comments. We have thoroughly revised the manuscript in response to your comments and believe that these changes have substantially improved its clarity and quality. Our detailed responses are provided below.

2. Have any measurements been made characterizing the stoichiometry of symport for DddT or any other related DMSP transporters?

Response: Thank you for this question. The stoichiometry of Na^+/DMSP symport for DddT was previously unknown. We have now measured it using solid-supported membrane (SSM) electrophysiology, and the results indicate a stoichiometry of 2 Na^+ ions per DMSP molecule (Appendix Figure S18). These results have been incorporated into the revised manuscript (Lines 344-347).

3. A more detailed description of the DMSP binding site should be included in the text. This description should be accompanied by a detailed presentation in Figure 3. The small inset in panel h is hard to interpret and it is unclear how specific residues interact with the substrate. These are key features of the transporter and should be clearly presented.

Response: Thank you for this important comment. We have now expanded the description of the DMSP binding site in the main text and revised Figure 3E to provide a more detailed presentation. The key residues interacting with DMSP are clearly highlighted, with their corresponding interactions to facilitate interpretation. The revised text is shown in Lines 254–261.

4. The modelled bond distances for several residues that are assigned as interacting with the proposed Na1 site are too far to directly interact with a bound Na. The geometry of the binding site should be carefully evaluated. This is particularly important for the C_cS structure in which the density of the proposed ion binding site is rather weak.

Response: Thank you for this valuable comment. We agree that the assignment of Na1 requires careful consideration. To address this, we have performed additional structural and functional experiments, as detailed below.

1) Improved cryo-EM maps

We re-collected and reprocessed the cryo-EM datasets for both the C_cS and C_e states, improving their overall resolutions to 2.52 Å and 2.69 Å, respectively. In the refined maps, the density corresponding to the Na1 site is more clearly defined, and an additional coordinating water molecule can now be resolved (Figure 4B). This improvement also allowed correction of the previously measured coordination distances (Figure 4B). Although the Na1–ligand distances in DddT remain slightly longer than those typically observed for tightly bound Na^+ ions, we consider them reasonable and consistent with a relatively labile, transient binding site. Na^+-binding sites in transporters can exhibit non-canonical coordination. For example, in the outward-occluded structure of LeuT, the identified Na1 site also exhibits longer coordination distances (Nature. 2012, 481:469-74), and in the BetP transporter, the proposed Na1 site displays a coordination pattern distinct from that of the stable Na2 site (Nat Commun, 2014, 5:4231). We consider this feature to be functionally relevant. Specifically, the identification of Na1 in our cryo-EM structures is likely facilitated by the high Na^+ concentrations present on both the periplasmic and cytoplasmic sides during sample preparation, which favors Na1 occupancy. Under physiological conditions, the lower cytoplasmic Na^+ concentration may instead promote Na1 dissociation, potentially influencing Na2 binding and the progression of the transport cycle. The clarification has been added to the revised manuscript (Lines 284-296, 323-331).

2) Structural validation under Na^+-free conditions

To further validate the assignment of the Na1 density, we collected cryo-EM data for the G101D mutant in a Na^+-free buffer. Under these conditions, the density corresponding to the Na1 site completely disappeared (Figure EV1 and Appendix FigureS15). Consistently, in the C_c-K^+ state structure of DddT, the corresponding Na1 density was also absent when the protein was prepared in a Na^+-free buffer (Figure EV1 and Appendix Fiure S10). Together, these observations strongly support the conclusion that the previously observed density at the Na1 site represents a specifically bound Na^+ ion rather than a water molecule or another cation.

Notably, under Na^+-free conditions, the G101D mutant adopts an inward-facing (C_i) conformation of DddT. This newly obtained structure fills a previous gap in our structural understanding of the inward-open state and suggests a possible allosteric coupling between Na^+ binding/release and the inward–outward conformational transitions of DddT. We have discussed this in the revised manuscript (Lines 230-238).

3) Transport stoichiometry

We further quantified Na^+ and DMSP fluxes to determine the transport stoichiometry of DddT. These measurements reveal a coupling ratio of approximately 2 Na^+ ions per DMSP molecule (Appendix Figure S18, Lines 344-347). This result supports the presence of two functional Na^+ binding sites (Na1 and Na2) and contradicts a transport mechanism mediated solely by a single Na2 site.

4) Comparison with other transporter families

We note that a Na^+ binding site equivalent to Na1 has not been previously resolved in available BCCT family structures. However, a Na^+-binding site located in a comparable region has been reported in members of the LeuT superfamily, although the coordinating residues

differ (Nat Commun, 2018, 9:1753), providing independent precedent for the existence of such a site.

Together with our previously reported mutational analyses, these new structural and functional data provide substantially strengthened evidence supporting the presence and mechanistic relevance of the Na1 site. We have revised the manuscript accordingly (Lines 230-238, 284-296, 323-331, and 344-347, Figures 4B and EV1). Moreover, we have toned down the wording regarding differences between the transport mechanisms of DddT and BetP. Rather than proposing distinct transport mechanisms, we now state that DddT and BetP possess different Na1-binding sites, which may lead to differences in their transport processes. In contrast, the Na2 site, which is essential for transport, is conserved and plays a central functional role in both systems (Lines 364-365).

5. Proline substitutions can have effects beyond altering the amino group of a peptide bond and thus care must be taken in interpreting their effects, particularly for residues near substrate-binding sites.

Response: Thank you for this comment. We have fully described the limitations of proline substitutions in the manuscript to avoid overinterpretation (Lines 303-306).

6. The resolution of the reported structures is too low to support the claim that "Chemically and structurally, the interaction of the DMSP molecule with the amino group of the peptide bond can affect the electron cloud distribution of the peptide bond, thereby enhancing the ability of oxygen to bind metal ions and stabilize the conformation of the peptide bond." If this statement is supported by other reports, they should be cited, and the context should be clarified.

Response: We agree that the original statement was not fully supported by the reported structures. Although the updated resolution has been improved (2.52 Å and 2.69 Å), it is still insufficient to substantiate this claim. Therefore, we have removed this statement to enhance the rigor and accuracy of the manuscript.

7. Several statements are similarly supported only weakly by the data and need to be revised: "Similarly, Na2 binding probably enhanced the interaction between the amino group of the peptide bond and the carboxyl group of DMSP, thereby stabilizing DMSP binding. Our results for the C_c-K^+ state confirm this point, showing that in the absence of Na^+, the addition of DMSP only results in the formation of the C_c state conformation without achieving the C_cS state conformation." Structural evidence should be presented to support an enhanced interaction between the amino group and the carboxyl of DMSP. The absence of a state in a cryo-EM analysis (in this case the C_c state) cannot inform on the effect of ligands on the conformational landscape when ~90% of the particles are excluded during data processing.

Response: Thank you for this important comment. To address this, we have removed the overinterpreted content, re-collected the cryo-EM data for the C_c-K^+ state to maximize the

number of particles used in the final reconstructions, and have rewritten the relevant section to ensure the rigor of our analysis.

"In the C_c-K^+ state, although DMSP was added, no electron density corresponding to DMSP was observed, consistent with the C_c state in the presence of NaCl without DMSP (Appendix Figure S12). Owing to the limitations of single-particle cryo-EM, we cannot completely exclude a minor population of DMSP-bound particles in the KCl sample. However, particle classification and reconstruction indicate that a huge number of particles adopt a DMSP-free conformation. In addition, only this population could be aligned and refined to high resolution, suggesting that the remaining particles likely represent conformationally unstable states. This contrasts sharply with the NaCl condition with DMSP, under which only the DMSP-bound conformation could be aligned and refined to high resolution. Taken together with data of the C_c, C_cS, C_i and C_e states, these findings suggest that stable DMSP binding requires Na^+, whereas stable Na^+ binding in turn depends on either DMSP or mutations in TMH3 (e.g., G101) that perturb the hinge region (Figures 3A and EV2). Thus, DMSP binding, Na^+ binding, and hinge conformation change are tightly coupled."

We have clarified these points in the manuscript (Lines 239-250) and moderated the original speculative statements accordingly.

8. Affinities cannot be assessed from the bond lengths determined from cryo-EM reconstructions. Please revise or remove the statements "Meanwhile, the binding affinity of the Na1 site for sodium ions appears to be reduced, as evidenced by the increased length of hydrogen bonds at the Na1-binding site in the C_cS state." "During the transport process, the binding affinity for Na1 appears to decrease, potentially leading to the release of Na1 in the C_cS state."

Response: Thank you for this comment. The statements have been removed from the manuscript.

9. Please clearly define in the discussion the evidence that the transport of DMSP and Na are cooperative.

Response: Thank you for this comment. We have revised the text to clearly define the evidence for cooperative transport of DMSP and Na^+. Structural comparisons of the different transport intermediate states indicate that stable DMSP binding requires the presence of Na^+, whereas stable Na^+ binding depends on either DMSP or mutations in TMH3 affecting the hinge region (Lines 218-220, 226-229, 236-238, 239-250).

10. A single molecular dynamics simulation is insufficient to presented as evidence supporting a transport model. The authors should be repeat the simulations multiple times and use statistical models to ensure that their interpretations are supported by the results.

Response: Thank you for this comment. We have now obtained a cryo-EM structure in the inward-facing conformation (Figure 3A, Appendix Figure S15), and accordingly, the previously included molecular dynamics simulation results, which were intended to model the inward-facing state, have been removed from the manuscript.

Minor comments.

11. It would be helpful to have the full name (Dimethylsulfoniopropionate) in the title of the report to help the broad and general audience of EMBO Journal.

Response: Thank you for this suggestion. Following your recommendation and the title length requirements of The EMBO Journal, we have revised the manuscript title to "Structural insights into bacterial dimethylsulfoniopropionate import by BCCT-family transporters."

12. The concentration of Na used to measure the K_m in Figure 1g should be reported in the figure legend to aid interpretation.

Response: Thank you for this comment. We have added the Na^+ concentration used to determine K_m, which was in excess, to the Figure 1G legend for clarity.

1st Revision - Editorial Decision **10th Apr 2026**

Dear Prof. Zhang,

Thank you for submitting your revised manuscript for consideration by the EMBO Journal. It has now been seen by the original referees whose comments are enclosed. As you will see, they express interest in your manuscript and are broadly in favour of publication, yet Referee #1 still has some reservations that require your attention.

Given the referees' positive recommendations, I would like to invite you to submit a revised version of the manuscript, addressing the remaining comments.

When preparing your letter of response to the referees' comments, please bear in mind that this will form part of the Review Process File, and will therefore be available online to the community. For more details on our Transparent Editorial Process, please review our Editorial Policies page: https://link.springer.com/partners/embo-press/editorial-policies

We generally allow three months as standard revision time. As a matter of policy, competing manuscripts published during this period will not negatively impact on our assessment of the conceptual advance presented by your study. However, we request that you contact the editor as soon as possible upon publication of any related work, to discuss how to proceed.

Thank you for the opportunity to consider your work for publication. I look forward to your revision.

Yours sincerely,

Yehu Moran
Academic Editor
The EMBO Journal

Instructions for preparing your revised manuscript:

Read our guidance for manuscript revisions and related editorial policies: https://link.springer.com/journal/44318/submission-guidelines#cms-Revised-submissions

Please also check that the title and abstract of the manuscript are brief, yet explicit, even to non-specialists.

When assembling figures, please refer to our figure preparation guideline in order to ensure proper formatting and readability in print as well as on screen:
https://media.springernature.com/original/springer-cms/rest/v1/content/27825798/data/v1

IMPORTANT: When you send the revision we will require
- a point-by-point response to the referees' comments, with a detailed description of the changes made (as a word file).
- a word file of the manuscript text.
- individual production quality figure files (one file per figure)
- a complete author checklist
- Expanded View files (replacing Supplementary Information)
- a Reagents and Tools Table as part of the Methods section

Please remember: Digital image enhancement is acceptable practice, as long as it accurately represents the original data and conforms to community standards. If a figure has been subjected to significant electronic manipulation, this must be noted in the figure legend or in the 'Methods' section. The editors reserve the right to request original versions of figures and the original images that were used to assemble the figure.

We realize that it is difficult to revise to a specific deadline. In the interest of protecting the conceptual advance provided by the work, we recommend a revision within 3 months (9th Jul 2026). Please discuss the revision progress ahead of this time with the editor if you require more time to complete the revisions.

Referee #1:

The authors present a comprehensive structural and functional characterization of the transporter DddT from Psychrobacter sp. D2, a member of the BCCT family that includes the well-studied prototype BetP. The study identifies DddT as a sodium-dependent transporter for dimethylsulfoniopropionate (DMSP), suggesting its role as a Na+-coupled symporter. This work is of broader significance because DMSP catabolism leads to the production of dimethylsulfide, a key compound in the global sulfur cycle and a climate-active gas. By combining cryo-EM structures, mutational analysis, in vivo functional assays, and phylogenetic analysis, the authors provide an extensive dataset to support a mechanistic model of transport.
In the revised manuscript, the authors have addressed the major concerns raised in the initial review in a largely satisfactory manner. Notably, they have incorporated substantial new experimental data including additional cryo-EM structures with improved resolution, of the sodium-free transporter, and of the transport compromised G101D variant captured in an inward-facing conformation. These additions substantially strengthen the overall interpretation. Furthermore, the update of description of results and discussion provide an enhanced interpretation of the data.
The presence of cryo- EM density consistent with putative Na+ ions is now better supported, although the unusually long coordinating distances at the proposed sodium-binding site remain atypical. While this issue is not fully resolved, the expanded dataset and improved discussion render the interpretation more convincing than in the original submission. Overall, the revised manuscript represents a substantial improvement, and the study appears suitable for publication.
That said, a note of caution remains regarding the interpretation of the sodium-binding sites. The cryo-EM structures used as "sodium-free controls" (Cc-K and Ci states) are resolved at above 3.0 Å, which is notably lower than the 2.5-2.8 Å resolution of the structures used to assign putative sodium-binding sites. Thus, there is a risk that resolution is not sufficient to resolve ions bound. Resolving such questions will require further investigation in the field.
Before publication, the following points should be addressed:
1. The revised manuscript cites the outward-occluded LeuT structure (Nature 2012, 481: 469-474) to justify unusually long (2.7-3.9 Å) Na1-ligand distances of the Ce (outward-open) and CcS (outward-occluded) states. However, in the LeuT study, the sodium coordination distances of the outward-open state are in the statistically valid range (all are in the range between 2.4-2.5 Å, pdb 3tt1). The long distances shown in the LeuT study in Fig. 4f highlight the change of distances of the superimposed inward-open and outward-occluded state, not the actual experimental determined coordination in the outward-occluded state (pdb 3tu0). Hence, the LeuT structure does not exhibit these unusually long sodium coordination distances and this argument should be removed.
2. The rebuttal letter states that contour levels in cryo-EM maps are now reported in RMSD; however, the figure legends still refer to sigma levels. This should be corrected.
Minor point
Line 134: "...produced higher levels of DMSP than the ΔdddT strain." This should read DMS (the product) rather than DMSP (the substrate). This issue was raised previously but remained uncorrected.

Referee #2:

The authors have greatly improved the manuscript in this revised version. All of my concerns have been addressed and it is now suitable for publication.

Responses to Reviewers

Line numbers refer to the PDF file generated by the submission system. In addition, a version of the manuscript with revisions highlighted is provided.

Reviewer 1:

The authors present a comprehensive structural and functional characterization of the transporter DddT from Psychrobacter sp. D2, a member of the BCCT family that includes the well-studied prototype BetP. The study identifies DddT as a sodium-dependent transporter for dimethylsulfoniopropionate (DMSP), suggesting its role as a Na+-coupled symporter. This work is of broader significance because DMSP catabolism leads to the production of dimethylsulfide, a key compound in the global sulfur cycle and a climate-active gas. By combining cryo-EM structures, mutational analysis, in vivo functional assays, and phylogenetic analysis, the authors provide an extensive dataset to support a mechanistic model of transport.

In the revised manuscript, the authors have addressed the major concerns raised in the initial review in a largely satisfactory manner. Notably, they have incorporated substantial new experimental data including additional cryo-EM structures with improved resolution, of the sodium-free transporter, and of the transport compromised G101D variant captured in an inward-facing conformation. These additions substantially strengthen the overall interpretation. Furthermore, the update of description of results and discussion provide an enhanced interpretation of the data.

The presence of cryo- EM density consistent with putative Na+ ions is now better supported, although the unusually long coordinating distances at the proposed sodium-binding site remain atypical. While this issue is not fully resolved, the expanded dataset and improved discussion render the interpretation more convincing than in the original submission. Overall, the revised manuscript represents a substantial improvement, and the study appears suitable for publication.

That said, a note of caution remains regarding the interpretation of the sodium-binding sites. The cryo-EM structures used as "sodium-free controls" (Cc-K and Ci states) are resolved at above 3.0 Å, which is notably lower than the 2.5-2.8 Å resolution of the structures used to assign putative sodium-binding sites. Thus, there is a risk that resolution is not sufficient to resolve ions bound. Resolving such questions will require further investigation in the field.

Response: We greatly appreciate your positive and valuable comments. We have carefully revised the manuscript according to your suggestions, and our detailed responses to your concerns are provided below. We fully understand your concerns regarding the relatively lower resolution of the C_c-K and C_i states. This issue will be further investigated in future studies.

Before publication, the following points should be addressed:

1. The revised manuscript cites the outward-occluded LeuT structure (Nature 2012, 481: 469-474) to justify unusually long (2.7-3.9 Å) Na1-ligand distances of the Ce (outward-open) and CcS (outward-occluded) states. However, in the LeuT study, the sodium coordination distances of the outward-open state are in the statistically valid range (all are in the range between 2.4-2.5 Å, pdb 3tt1). The long distances shown in the LeuT study in Fig. 4f highlight the change of distances of the superimposed inward-open and outward-occluded state, not the actual experimental determined coordination in the

outward-occluded state (pdb 3tu0). Hence, the LeuT structure does not exhibit these unusually long sodium coordination distances and this argument should be removed.

Response: Thank you very much for pointing out this important issue. Upon re-evaluation, we concur that our previous description and interpretation of the LeuT structures were not accurate. We have revised the manuscript accordingly and removed this discussion (Lines 285-287).

2. The rebuttal letter states that contour levels in cryo-EM maps are now reported in RMSD; however, the figure legends still refer to sigma levels. This should be corrected.

Response: Thank you for identifying this error. The figure legends have been corrected accordingly.

3. Minor point

Line 134: "...produced higher levels of DMSP than the ΔdddT strain." This should read DMS (the product) rather than DMSP (the substrate). This issue was raised previously but remained uncorrected.

Response: We apologize for this typo. We have corrected the manuscript and replaced “DMSP” with “DMS” at Line 133.

<u>Reviewer 2:</u>

The authors have greatly improved the manuscript in this revised version. All of my concerns have been addressed and it is now suitable for publication.

Response: We sincerely appreciate the reviewer for the positive evaluation of our revised manuscript and for endorsing publication.

Dear Prof. Zhang,

I am pleased to inform you that your manuscript has been accepted for publication in the EMBO Journal.

Your manuscript will be processed for publication by EMBO Press. It will be copy edited and you will receive page proofs prior to publication. Please note that you will be contacted by Springer Nature Author Services to complete licensing and payment information.

You may qualify for financial assistance for your publication charges - either via a Springer Nature fully open access agreement or an EMBO initiative. Check your eligibility: https://link.springer.com/journal/44318/how-to-publish-with-us

Should you be planning a Press Release on your article, please get in contact with embo_production@springernature.com as early as possible in order to coordinate publication and release dates.

If you have any questions, please do not hesitate to contact the Editorial Office. Thank you for your contribution to The EMBO Journal.

Yours sincerely,

Yehu Moran
Academic Editor
The EMBO Journal

*****
Please note that it is The EMBO Journal policy for the transcript of the editorial process (containing referee reports and your response letters) to be published as an online supplement to each paper. If you should prefer removal of any referee-only figures included in the point-by-point response(s), e.g. because they may still be used for future publication or because they have been reproduced from published work by others, please do let us know immediately via response email.
More information is available here: https://link.springer.com/partners/embo-press/editorial-policies#Peer%20review

EMBO Press Author Checklist

Corresponding Author Name: Yu-Zhong Zhang, Lu-Ning Liu, Peng Wang
Journal Submitted to: The EMBO Journal
Manuscript Number: Manuscript EMBOJ-2025-121988R

USEFUL LINKS FOR COMPLETING THIS FORM
The EMBO Journal - Author Guidelines
EMBO Reports - Author Guidelines
Molecular Systems Biology - Author Guidelines
EMBO Molecular Medicine - Author Guidelines

Reporting Checklist for Life Science Articles (updated January
This checklist is adapted from Materials Design Analysis Reporting (MDAR) Checklist for Authors. MDAR establishes a minimum set of requirements in transparent reporting in the life sciences (see Statement of Task: 10.31222/osf.io/9sm4x). Please follow the journal's guidelines in preparing your
Please note that a copy of this checklist will be published alongside your article.

Abridged guidelines for figures
1. Data
The data shown in figures should satisfy the following conditions:
- the data were obtained and processed according to the field's best practice and are presented to reflect the results of the experiments in an accurate and unbiased manner.
- ideally, figure panels should include only measurements that are directly comparable to each other and obtained with the same assay.
- plots include clearly labeled error bars for independent experiments and sample sizes. Unless justified, error bars should not be shown for technical
- if n<5, the individual data points from each experiment should be plotted. Any statistical test employed should be justified.
- Source Data should be included to report the data underlying figures according to the guidelines set out in the authorship guidelines on Data

2. Captions
Each figure caption should contain the following information, for each panel where they are relevant:
- a specification of the experimental system investigated (eg cell line, species name).
- the assay(s) and method(s) used to carry out the reported observations and measurements.
- an explicit mention of the biological and chemical entity(ies) that are being measured.
- an explicit mention of the biological and chemical entity(ies) that are altered/varied/perturbed in a controlled manner.
- the exact sample size (n) for each experimental group/condition, given as a number, not a range;
- a description of the sample collection allowing the reader to understand whether the samples represent technical or biological replicates (including how many animals, litters, cultures, etc.).
- a statement of how many times the experiment shown was independently replicated in the laboratory.
- definitions of statistical methods and measures:
  - common tests, such as t-test (please specify whether paired vs. unpaired), simple χ2 tests, Wilcoxon and Mann-Whitney tests, can be unambiguously identified by name only, but more complex techniques should be described in the methods section;
  - are tests one-sided or two-sided?
  - are there adjustments for multiple comparisons?
  - exact statistical test results, e.g., P values = x but not P values < x;
  - definition of 'center values' as median or average;
  - definition of error bars as s.d. or s.e.m.

Please complete ALL of the questions below.
Select "Not Applicable" only when the requested information is not relevant for your study.

Materials

Newly Created Materials	Information included in the manuscript?	In which section is the information available? (Reagents and Tools Table, Materials and Methods, Figures, Data Availability Section)
New materials and reagents need to be available; do any restrictions apply?	Not Applicable	

Antibodies	Information included in the manuscript?	In which section is the information available? (Reagents and Tools Table, Materials and Methods, Figures, Data Availability Section)
For **antibodies** provide the following information:<br>- Commercial antibodies: RRID (if possible) or supplier name, catalogue number and or/clone number<br>- Non-commercial: RRID or citation	Not Applicable	

DNA and RNA sequences	Information included in the manuscript?	In which section is the information available? (Reagents and Tools Table, Materials and Methods, Figures, Data Availability Section)
**Short novel DNA or RNA including primers, probes**: provide the sequences.	Yes	Appendix Table S3

Cell materials	Information included in the manuscript?	In which section is the information available? (Reagents and Tools Table, Materials and Methods, Figures, Data Availability Section)
**Cell lines:** Provide species information, strain. Provide accession number in repository **OR** supplier name, catalog number, clone number, and/**OR** RRID.	Not Applicable	
**Primary cultures:** Provide species, strain, sex of origin, genetic modification status.	Not Applicable	
Report if the cell lines were recently **authenticated** (e.g., by STR profiling) and tested for mycoplasma contamination.	Not Applicable	

Experimental animals	Information included in the manuscript?	In which section is the information available? (Reagents and Tools Table, Materials and Methods, Figures, Data Availability Section)
**Laboratory animals or Model organisms:** Provide species, strain, sex, age, genetic modification status. Provide accession number in repository **OR** supplier name, catalog number, clone number, **OR** RRID.	Not Applicable	
**Animal observed in or captured from the field:** Provide species, sex, and age where possible.	Not Applicable	
Please detail **housing and husbandry conditions**.	Not Applicable	

Plants and microbes	Information included in the manuscript?	In which section is the information available? (Reagents and Tools Table, Materials and Methods, Figures, Data Availability Section)
**Plants:** provide species and strain, ecotype and cultivar where relevant, unique accession number if available, and source (including location for collected wild specimens).	Not Applicable	
**Microbes:** provide species and strain, unique accession number if available, and source.	Yes	Reagents and Tools Table

Human research participants	Information included in the manuscript?	In which section is the information available? (Reagents and Tools Table, Materials and Methods, Figures, Data Availability Section)
If collected and within the bounds of privacy constraints report on age, sex and gender or ethnicity for all study participants.	Not Applicable	

Core facilities	Information included in the manuscript?	In which section is the information available? (Reagents and Tools Table, Materials and Methods, Figures, Data Availability Section)
If your work benefited from core facilities, was their service mentioned in the acknowledgments section?	Not Applicable	

Design

Study protocol	Information included in the manuscript?	In which section is the information available? (Reagents and Tools Table, Materials and Methods, Figures, Data Availability Section)
If study protocol has been **pre-registered, provide DOI in the manuscript**. For clinical trials, provide the trial registration number **OR** cite DOI.	Not Applicable	
Report the **clinical trial registration number** (at ClinicalTrials.gov or equivalent), where applicable.	Not Applicable	

Laboratory protocol	Information included in the manuscript?	In which section is the information available? (Reagents and Tools Table, Materials and Methods, Figures, Data Availability Section)
Provide DOI OR other citation details if **external detailed step-by-step protocols** are available.	Not Applicable	

Experimental study design and statistics	Information included in the manuscript?	In which section is the information available? (Reagents and Tools Table, Materials and Methods, Figures, Data Availability Section)
Include a statement about **sample size** estimate even if no statistical methods were used.	Not Applicable	
Were any steps taken to minimize the effects of subjective bias when allocating animals/samples to treatment (e.g. **randomization procedure**)? If yes, have they been described?	Not Applicable	
Include a statement about **blinding** even if no blinding was done.	Not Applicable	
Describe **inclusion/exclusion criteria** if samples or animals were excluded from the analysis. Were the criteria pre-established? If sample or data points were omitted from analysis, report if this was due to attrition or intentional exclusion and provide justification.	Not Applicable	
For every figure, are **statistical tests** justified as appropriate? Do the data meet the assumptions of the tests (e.g., normal distribution)? Describe any methods used to assess it. Is there an estimate of variation within each group of data? Is the variance similar between the groups that are being statistically compared?	Yes	Figure legends of Figure 1, 3, 4

Sample definition and in-laboratory replication	Information included in the manuscript?	In which section is the information available? (Reagents and Tools Table, Materials and Methods, Figures, Data Availability Section)
In the figure legends: state number of times the experiment was **replicated** in laboratory.	Yes	Figure legends of Figure 1, 3, 4
In the figure legends: define whether data describe **technical or biological replicates**.	Yes	Figure legends of Figure 1, 3, 4

Ethics

Ethics	Information included in the manuscript?	In which section is the information available? (Reagents and Tools Table, Materials and Methods, Figures, Data Availability Section)
Studies involving **human participants**: State details of **authority granting ethics approval** (IRB or equivalent committee(s), provide reference number for approval.	Not Applicable	
Studies involving **human participants**: Include a statement confirming that **informed consent** was obtained from all subjects and that the experiments conformed to the principles set out in the WMA Declaration of Helsinki and the Department of Health and Human Services Belmont Report.	Not Applicable	
Studies involving **human participants: For** publication of **patient photos**, include a statement confirming that consent to publish was obtained.	Not Applicable	
Studies involving experimental **animals**: State details of **authority granting ethics approval** (IRB or equivalent committee(s), provide reference number for approval. Include a statement of compliance with ethical regulations.	Not Applicable	
Studies involving **specimen and field samples:** State if relevant **permits** obtained, provide details of authority approving study; if none were required, explain why.	Not Applicable	

Dual Use Research of Concern (DURC)	Information included in the manuscript?	In which section is the information available? (Reagents and Tools Table, Materials and Methods, Figures, Data Availability Section)
Could your study fall under dual use research restrictions? Please check biosecurity documents and list of **select agents and toxins** (CDC): https://www.selectagents.gov/sat/list.htm	Not Applicable	
If you used a select agent, is the security level of the lab appropriate and reported in the manuscript?	Not Applicable	
If a study is subject to dual use research of concern regulations, is the name of the **authority granting approval and reference number** for the regulatory approval provided in the manuscript?	Not Applicable	

Reporting

The MDAR framework recommends adoption of discipline-specific guidelines, established and endorsed through community initiatives. Journals have their own policy about requiring specific guidelines and recommendations to complement MDAR.

Adherence to community standards	Information included in the manuscript?	In which section is the information available? (Reagents and Tools Table, Materials and Methods, Figures, Data Availability Section)
State if relevant guidelines or checklists (e.g., **ICMJE, MIBBI, ARRIVE, PRISMA**) have been followed or provided.	Not Applicable	
For **tumor marker prognostic studies,** we recommend that you follow the **REMARK** reporting guidelines (see link list at top right). See author guidelines, under 'Reporting Guidelines'. Please confirm you have followed these guidelines.	Not Applicable	
For **phase II and III randomized controlled trials**, please refer to the **CONSORT** flow diagram (see link list at top right) and submit the CONSORT checklist (see link list at top right) with your submission. See author guidelines, under 'Reporting Guidelines'. Please confirm you have submitted this list.	Not Applicable	

Data Availability

Data availability	Information included in the manuscript?	In which section is the information available? (Reagents and Tools Table, Materials and Methods, Figures, Data Availability Section)
Have **primary datasets** been deposited according to the journal's guidelines (see 'Data Deposition' section) and the respective accession numbers provided in the Data Availability Section?	Yes	Data availability
Were **human clinical and genomic datasets** deposited in a public access-controlled repository in accordance to ethical obligations to the patients and to the applicable consent agreement?	Not Applicable	
Are **computational models** that are central and integral to a study available without restrictions in a machine-readable form? Were the relevant accession numbers or links provided?	Not Applicable	
If publicly available data were reused, provide the respective **data citations in the reference list**.	Yes	Materials and Methods
