# Supplementary material for: Structural insights into bacterial dimethylsulfoniopropionate import by BCCT-family transporters
Source: EMBO J. 2026 May 8;45(12):4299–320. doi: 10.1038/s44318-026-00798-w (PMC13270143; doi:10.1038/s44318-026-00798-w)
Supplement: Supplementary file 9 — Appendix Figure Source Data [file 44318_2026_798_MOESM9_ESM.zip › Appendix_Figure_S17.zip!/Appendix Figure S17/Appendix Figure S17A/README.rtf]

Western blot analysis of DddT and its mutant variants performed in four independent experimental batches.
In Anti-His 1 and Anti-GAPDH 1, samples were loaded in the following order: Marker, DddT, A99P, S204A, W140A, W320A, W321A, Y324A, W105A, Y112A, CK, Marker, Marker.
In Anti-His 2 and Anti-GAPDH 2, samples were loaded in the following order: Marker, DddT, Y112F, W313A, F316A, Y317A, T418P, L252P, S256A, D421A, CK, Marker, Marker.
In Anti-His 3 and Anti-GAPDH 3, samples were loaded in the following order: Marker, DddT, S422F, S422A, C95P, V98P, F415P, T418A, T419A, G101D, CK, Marker, Marker, Marker.
In Anti-His 4 and Anti-GAPDH 4, samples were loaded in the following order: Marker, Marker, DddT, M92P, CK, Marker.
